# Ultra-high THz-field-confinement at LaAlO_3_ twin walls

**DOI:** 10.1038/s41467-026-77689-5

**Published:** 2026-09-28

**Authors:** J. Wetzel, J. Taboada-Gutiérrez, M. Roeper, F. G. Kaps, G. L. Esposito, D. Marchese, R. Buschbeck, P. Lenz, J. M. Klopf, H. A. Bechtel, S. N. Gilbert Corder, J. Teyssier, S. C. Kehr, L. M. Eng, A. B. Kuzmenko, S. D. Seddon

**Affiliations:** 1 Institute of Applied Physics, TUD Dresden University of Technology, Dresden, Germany; 2 Würzburg-Dresden Cluster of Excellence (EXC2147) ctd.qmat – Complexity, Topology, and Dynamics in Quantum Matter, Dresden, Germany; 3 University of Geneva, Department of Quantum Matter Physics (DQMP), Geneva, Switzerland; 4 Institute of Radiation Physics, Helmholtz-Zentrum Dresden Rossendorf, Dresden, Germany; 5 Advanced Light Source (ALS), Lawrence Berkeley National Laboratory, Berkeley, CA USA

**Keywords:** Condensed-matter physics, Optical materials and structures

## Abstract

The control and steering of light at nanometre length scales is crucial for the development of both fundamental science and nanophotonic technologies. Recent advancements have been achieved by exploiting various crystalline anisotropies, allowing for subdiffractional and diffraction-less canalisation of energy. These studies in particular benefit from surface structuring as well as from stacking and twisting of 2D materials, whereas corresponding capabilities of anisotropic bulk crystals are rather unexplored. In this work, we show that ferroelastic twin walls – crystallographically perfect 2D-sheets that separate regions of differently oriented domains – in the distorted perovskite LaAlO_3_ provide a natural platform for broadband lateral confinement of light at the nanoscale. Without fabrication processes, the electromagnetic fields localised at such walls exhibit lateral optical sizes up to 260 times smaller than the free-space wavelength. Depending on the adjacent domain orientation and frequency, the twin wall pattern preferentially concentrates or repels the electromagnetic energy, constituting a natural building block towards broadband MIR and THz nanophotonics for polaritonic circuitry.

## Abstract (web summary)

Naturally occurring ferroelastic twin walls in LaAlO_3_ confine terahertz and mid-infrared light to 260 times below the free-space wavelength with no nanofabrication required. The walls form a natural, spanning network, which is tunable by frequency.

## Introduction

The control, manipulation and steering of light at nanometre length scales is a central goal of nano-optics^1–4^. Polar crystals provide an attractive platform because they support phonon polaritons (PhPs)^5^, hybrid light-matter modes formed by coupling electromagnetic waves to lattice vibrations in polar insulators. With reduced symmetry, such crystals enable field control via various types of PhPs, e.g., found in anisotropic vdW materials^6–8^. Specifically in hyperbolic regimes^9^ — where different components of the dielectric permittivity tensor have opposite signs — such PhPs can propagate highly directional with deeply sub-wavelength confinement^10–18^. A particularly promising application is the concentration of the PhP’s energy flow along specific directions even suppressing lateral diffraction^19–21^. In most current approaches, this phenomenon is engineered through the stacking and twisting of layers of polar materials^2,16,19–21^ and then adding nanopatterned elements (e.g., e-beam/FIB-defined launchers, reflectors, or resonators) to control PhP excitation. Propagation of light at the nanoscale with high directionality in pristine crystals^22^ is typically restricted to narrow spectral windows close to phonon resonances, suffering from limited propagation lengths and diffraction. Consequently, a platform that provides strong confinement and long-range propagation while avoiding complex fabrication addresses key limitations of existing state-of-the-art nano-optical devices and will be highly relevant for THz/MIR nano-optics and spectroscopy.

In this work, we introduce ferroelastic twin walls (TWs) as a suitable and effective platform for confining and guiding light at the nanoscale without the necessity of complex fabrication processes. These so-called ferroelastic materials undergo a spontaneous crystal symmetry breaking below a certain ordering temperature (on the order of hundreds of °C), resulting in several possible crystallographic domains, that co-exist and tessellate throughout the material^23^. These regions are delineated by TWs, which are mirror planes, that form a well-defined pattern through the material, constituting uncharged, non-polar interfaces aligned along specific crystallographic angles, separating two domains of the same material with distinct orientations of their anisotropy axis. Furthermore, as TWs form in certain symmetry-defined planes and represent a perfect boundary between two ferroelastic domains, they therefore can be thought of as atomically sharp, pristine interfaces. Ferroelastic twinning is a common phenomenon in nature, which is notably present in various minerals and materials such as calcite (CaCO_3_), quartz (SiO_2_), feldspar, or lanthanum aluminate (LaAlO_3_, LAO), as used here. Indeed feldspars alone make up 30% of the earth’s crust^24^.

LAO is one of the most versatile complex oxides for functional interfaces and heterostructures, as it can act both as a polar active layer and as epitaxial substrate. In the former case, it has been successfully employed for studying and controlling high mobility 2D electron gases^25–27^, and for the control of quantum phases and quantum circuitry^28,29^. In the latter case, LAO stabilises strained phases of oxide films such as BiFeO_3_^30,31^ or NdNiO_3_^32^, for enhancing their multiferroic behaviour or controlling edge-confined polaritons in the epitaxially grown thin film layer, respectively, among other applications. Furthermore, as part of the ferroelastic material class, it has been recently shown, that its crystal structure and domain arrangement can be controlled via applied strain^33^. Taken together, these properties make LAO a powerful platform for engineering functional devices, nanoscale control of ferroic order and enhanced light-matter coupling in thin films.

Using scattering scanning near-field optical microscopy (s-SNOM) at room temperature, analytical dipole modelling, and finite-element method (FEM) simulations, we demonstrate here ultra-high electromagnetic lateral field confinement at LAO TWs in the technologically relevant terahertz (THz) and mid-infrared (MIR) ranges. As a function of the excitation frequency, we demonstrate that TWs form a tuneable network across the crystal for the confinement of light at the nanoscale, with frequency-selective paths for hosting the enhanced near field.

## Results

### Optical anisotropy and twin-wall pattern

**Fig. 1:**
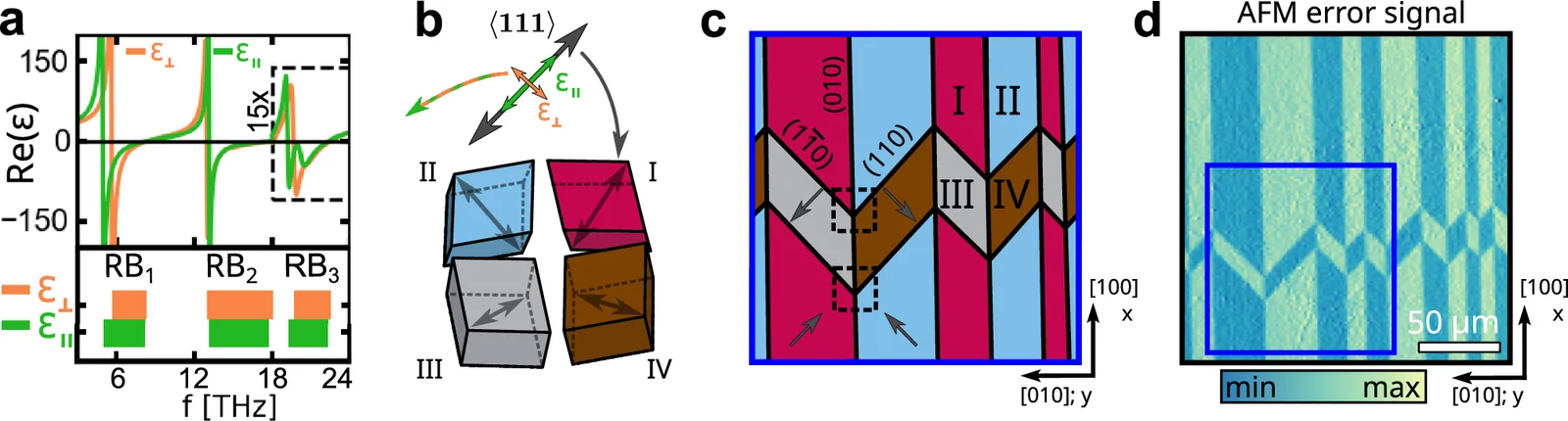
LaAlO_3_ properties and twin domains. **a** (upper) Anisotropy in the dielectric permittivity ε decomposed into perpendicular (orange, ${\varepsilon }_{\perp }$) and parallel (green, ${\varepsilon }_{\parallel }$) components as defined with respect to the LAO optical axis. (lower) The three Reststrahlen bands spectral positions shown for the same permittivity components. **b** (top) Schematic indicating the orientations of ${\varepsilon }_{\parallel }$ and ${\varepsilon }_{\perp }$ relative to the strain axis (grey arrow), coinciding with the pseudocubic [111] direction. (bottom) Sketch of the four pseudocubic crystallographic domains of LAO (I-IV), compressively distorted along the [111] direction. Domains II–IV are $90^\circ$ rotations of domain I. **c** The allowed tiling of a LAO(001) surface following the crystal twinning rules. The optical axes of the four domains are indicated by grey arrows pointing out of plane. Vertical and $45^\circ$ TWs are labelled according to their Miller indices and their possible TWJs are marked with dashed boxes. **d** Atomic force microscopy (AFM) error signal image indicating relative tilts of the sample surface and the twin domains. The blue box indicates the sketched area in (**c**).

In this study LAO, a rhombohedrally distorted pseudocubic perovskite (space group ${\mathrm{R}}{\bar{3}}{\mathrm{c}}$) is investigated^34^. LAO is optically uniaxial, here its permittivity can be decomposed into two components, ${\varepsilon }_{\parallel }$ and ${\varepsilon }_{\perp }$, defined parallel and perpendicular to the optical axis aligned with the pseudocubic [111] direction (coincident with the spontaneous compressive strain axis). The full tensor and its decomposition along the crystallographic axes can be found in the SI, section S1. In the MIR–THz range the dielectric function exhibits three high-reflectivity Reststrahlen bands (RBs)^35–37^, where $\mathrm{Re}(\varepsilon )$ becomes negative and PhPs can be excited. Figure 1a shows the real parts of the uniaxial dielectric permittivity components of LAO extracted from polarised Fourier transform infrared spectroscopy (FTIR) (see Suppl. Information S2). Notably, the three RBs for each of the permittivity components show a small frequency shift between their corresponding phonon resonances, introducing an elliptic or hyperbolic response within all RBs.

As all ferroelastic crystals, LAO forms multiple symmetry-related crystallographic domains. On the LAO(001) surface, four domain variants are allowed to form. These can be visualised by taking a cube distorted along its [111] direction (blue domain labelled “II” in Fig. 1b) and rotating it in-plane around the [001] axis three successive times by 90°. This generates domains III, IV, and I, with their respective optical axes oriented along [$\bar{1}11$], [$\bar{1}\bar{1}1$], and [$1\bar{1}1$].

These four domains are separated by four distinct TWs (Fig. 1c)^23,34^, that are determined by crystal symmetry and strain compatibility^38^, forming exact mirror planes for the adjacent domains’ crystal structures. The resulting domain tessellation forms the characteristic “chevron” tiling commonly observed in ferroelastic crystals^23,39–41^. The TW planes are all oriented strictly orthogonal to the (001) surface and extend through the full sample thickness; on the surface, however, they follow several crystal directions, revealing two distinct types of TWs: long vertical TWs aligned with the (010) plane, and 45° zig-zag TWs running along the [110] / $[1\bar{1}0]$ axes, shown in Fig. 1c. Consequently, adjacent domains arrange themselves in alternating pairs, namely, I-III and II-IV in vertical directions. The TWs meet at twin-wall junctions (TWJs), where all four domains and all four walls intersect (see dashed boxes in Fig. 1c).

This specific spatial configuration of domains in LAO(001) has two critical consequences for the 3D orientation of the optical axes: Firstly, the out-of-plane components of the optical axes are identical across all domains. In contrast, the in-plane projections of adjacent domains either orientate parallel or orthogonal to one another, depending on the TW type (zig-zag or vertical TWs, respectively). In Fig. 1c the projections of the optical axes onto the (001) surface are indicated as grey arrows, with arrowheads pointing out of the plane to illustrate their 3D orientations. Secondly, at each TW, the orientation of the optical axis of adjacent domains is mirrored at the TW-plane. Consequently, when crossing a vertical (010) TW (such as those between I and II, or III and IV) the optical axis rotates 90 degrees, but when crossing a diagonal, zig-zag wall (like those between I and III or II and IV) the optical axis is rotated 180 degrees, so the plane the optical axis operates along is unchanged in this latter case. Note that independent of their in-plane orientation, the inclination direction of the optical axis towards the sample surface flips at each of the TWs due to the mirror symmetry, resulting in the “head-to-head” and “tail-to-tail” configurations as indicated by the grey arrows in Fig. 1c. A more detailed description of the domain formation and its optical axis orientation along with a depiction of all possible TW configurations is given in the Suppl. Information S3.

The domain arrangement is directly observed by atomic force microscopy. The AFM error-signal image shown in Fig. 1d (effectively the lateral derivative of the height topography) highlights regions of constant surface tilt of about ±0.075°^23^ associated with the rhombohedral angles of the four domains, yielding a terraced surface morphology consistent with the schematic in Fig. 1c.

### THz nano-imaging of twin walls

**Fig. 2:**
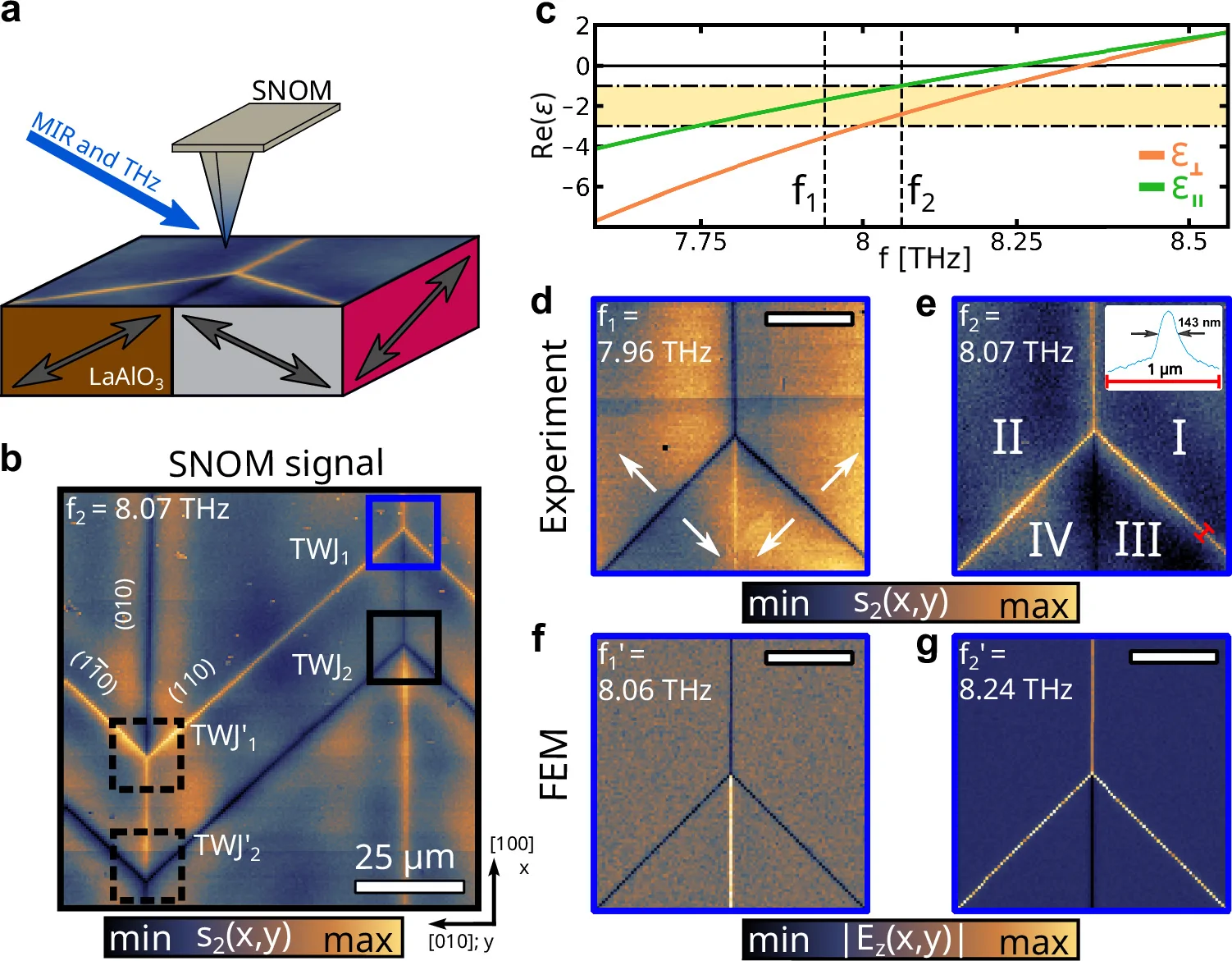
Frequency-dependent response of twin walls. **a** Free-electron-laser-illuminated s-SNOM measurements reveal sharp optical contrast emergent from TWs, with the optical axis projections of the twin domains indicated as grey arrows. Incoming light ranges from the MIR to THz spectral regime. **b** s-SNOM image of all four possible TW junctions (TWJs), taken at an illumination frequency of ${f}_{2}=8.07\,\mathrm{THz}$. **c** Real part of the dielectric permittivity ε in the relevant spectral regime, decomposed into perpendicular (orange, ${\varepsilon }_{\perp }$) and parallel (green, ${\varepsilon }_{\parallel }$) components, defined relative to the LAO optical axes. **d,** **e** s-SNOM images acquired at TWJ_1_ as a function of illumination frequencies ${f}_{1}$ and ${f}_{2}$ (as specified in (**d**, **e**) respectively and marked in (**c**). Inset in (**e**) shows the line profile across a bright (110) TW. **f**, **g** FEM simulations of the same TWJ_1_ at frequencies ${f}_{1}^{\prime}$ and ${f}_{2}^{\prime},$ which are spectrally slightly shifted to match the contrast observed in the experiments (**d**, **e**). Scale bars in (**d**–**g**) correspond to $10\,\upmu {\rm{m}}$.

To determine the near-field response at TWs, THz-s-SNOM measurements have been performed at room temperature under ambient conditions, utilising a tuneable free-electron laser (FEL) as narrowband light source^42^ (FEL-SNOM, see Fig. 2a and Methods). Figure 2b shows a FEL-SNOM image ${s}_{2}(x,\,y)$ (second-harmonic demodulation of the tip tapping frequency) at ${f}_{2}=8.07\,\mathrm{THz}$ (${\lambda }_{2}\approx 37.15\,\upmu {\rm{m}}$, ${\omega }_{2}\approx 269.2\,{{\rm{cm}}}^{-1}$) in a sample region containing several TWs and TWJs. This frequency lies within RB_1_ close to the LO phonon mode, where both ${\varepsilon }_{\parallel }$ and ${\varepsilon }_{\perp }$ are slightly negative and highly dispersive (Fig. 2c). The near-field map exhibits a set of bright and dark contrasts (relative to the bulk LAO signal) localised at TWs positions.

A striking feature is that the near-field contrast at the TWs depends on the orientation of the neighbouring LAO domains. Any vertical (010) TW experiences a contrast inversion when crossing a TWJ, whereas the zig-zag TWs remain consistently bright or consistently dark along their length, depending in all cases on the optical axis orientation in the adjacent domains. Considering the arrow representation of the optical axes as indicated in Fig. 1, “head-to-head” configurations appear dark and “tail-to-tail” TWs bright at this frequency. This repeatable and robust pattern defines two easily identifiable junction types, TWJ_1_ and TWJ_2_ (blue and black rectangles in Fig. 2b); two further junctions (TWJ_1_′, TWJ_2_′) are $180^\circ$-rotated counterparts and behave equivalently.

### Frequency-dependent contrast inversion and experimental confinement

In order to probe the spectral response and lateral field confinement, TWJ_1_ (blue square in Fig. 2b) was imaged at two nearby frequencies, ${f}_{1}=7.96\,\mathrm{THz}$ (${\lambda }_{1}\approx 37.66 \,\upmu {\rm{m}}$, ${\omega }_{1}\approx 265.5\,{{\rm{cm}}}^{-1}$) and ${f}_{2}=8.07\,\mathrm{THz}$ (vertical dashed lines in Fig. 2c), which were experimentally found to show the highest contrast. These frequencies are within the typically observed resonance regime in SNOM from $\mathrm{Re}(\varepsilon )=-3$ to $\mathrm{Re}(\varepsilon )=-1$, depending on tip-sample distance^43^. Strikingly, the bright–dark pattern of the junction inverts completely between these frequencies (Fig. 2d, e). Although only one RB is displayed here, a similar behaviour has been observed in all 3 RBs as shown in the Suppl. Information S4. A line profile extracted from a high-resolution scan of TWJ_1_ at ${f}_{2}$ (see inset in Fig. 2e) yields an optical ${FWHM}=143\,\mathrm{nm}$, corresponding to a lateral confinement factor of $\lambda /{FWHM}=260$ relative to the excitation wavelength (see Suppl. Information S4). This value is approximately one order of magnitude larger than previously reported lateral confinement factors in highly directional polaritonic systems, which are typically around 40^17,21^, while being realised in a bulk LAO crystal where the confinement is provided by naturally occurring ferroelastic twin walls rather than in twisted 2D materials. However, one should note that considering that the measured FWHM is a convolution of the field confinement and tip radius, this value is an upper bound, and the true confinement factor is likely to be even higher as confirmed below by numerical simulations.

### FEM simulations and origin of the contrast

To understand the contrast inversion and the highly confined fields, the tip–sample interaction was calculated through FEM simulations, as it has successfully been employed in previous works to explain the image contrast in s-SNOM experiments^44–47^ (see Methods). In these simulations, a vertically-oriented point dipole is scanned laterally above the sample at ${f}_{1}^{\prime} \approx 8.06\,\mathrm{THz}$ (${\lambda }_{1}^{\prime} \approx 37.17\,\upmu {\rm{m}}$, ${{\omega }_{1}}^{\prime}=\,269.0\,{{\rm{cm}}}^{-1}$) and ${f}_{2}^{\prime} \approx 8.24\,\mathrm{THz}$ (${\lambda }_{2}^{\prime} \approx 36.36\,\upmu {\rm{m}}$, ${{\omega }_{2}}^{\prime}=\,275.0\,{{\rm{cm}}}^{-1}$), selected to show highest contrast in the simulations. Notably, the simulations perfectly reproduce the bright/dark contrast of the measured ${s}_{2}(x,\,y)$ images at ${f}_{1}$ and ${f}_{2}$ (Fig. 2f, g versus Fig. 2d, e), including the strong field localisation at the walls. Note that the simulation surface is perfectly flat, demonstrating that the contrast is driven by the local in-plane rotation of the optical axis between the neighbouring domains and not by any topography-related effect, such as observed in ridge-like modes^48^. In fact, all s-SNOM measured samples have been polished for removing any such surface tilt. The spectral shift of the simulations towards slightly higher frequencies compared to the experimental results is consistent with the frequency shift previously reported for s-SNOM simulations utilising the point-dipole approximation^49^. Intriguingly, the simulations even capture the small but systematic brightness difference between the vertical (010) and the $45^\circ$ zig-zag walls (Fig. 2e, g), which follows from neighbouring optical axes meeting at an in-plane angle of $90^\circ$ (for (010) walls) or $180^\circ$ (for the $45^\circ$ zig-zag walls). Merely the weak experimental intensity gradients next to some walls are not reproduced by the simulations. These gradients are attributed to the convolution of 2 effects: surface-PhPs launched by the tip and weakly reflected by the TW, and illumination-related effects. In fact, in complementary synchrotron infrared nano-spectroscopy (SINS) measurements, the excitation of non-strongly confined SPhPs at the surface of bulk LAO(001) through an Au structure has been confirmed in this spectral regime (see Methods and Suppl. Information S5), demonstrating that propagating SPhPs travel through the LAO(001) surface crossing the ferroelastic TWs with very little impact on their propagation.

### Modified dipole model and optical contrast mechanism at the walls

**Fig. 3:**
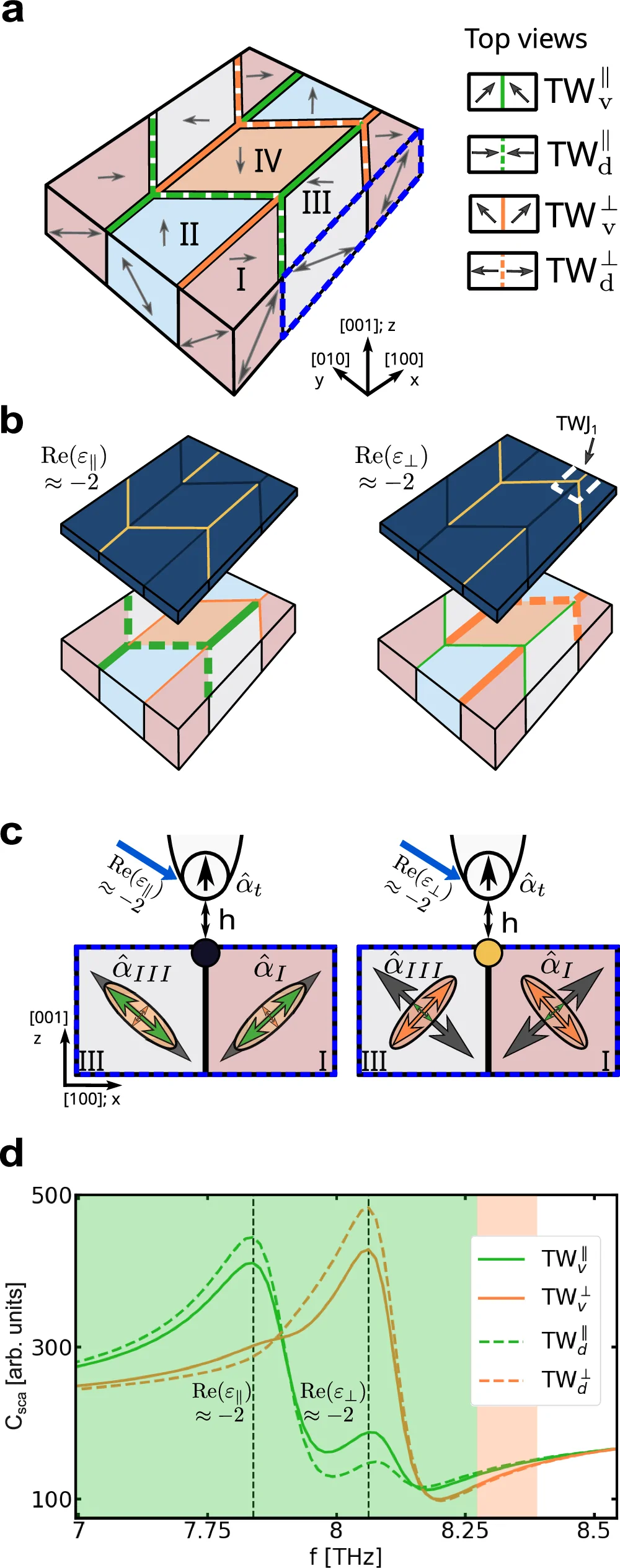
Contrast mechanism at the twin walls. **a** Schematic showing the four types of TWs present in the system, characterised by two groups marked orange and green. Orange (green) TWs show an enhanced response when the perpendicular (parallel) permittivity component is resonantly excited. Optical axis projections are marked as double arrows on the sides, while single arrows indicating their surface projection direction with an out-of-plane component (arrowhead pointing out of the surface, compare Fig. 1c). **b** lower row: TWs selected for enhanced fields at frequencies where $\mathrm{Re}\left({\varepsilon }_{\parallel }\right)$ and $\mathrm{Re}\left({\varepsilon }_{\perp }\right)$ match the near-field resonance condition $\mathrm{Re}\left(\varepsilon \right)=-3 \; \mathrm{to}-1$, respectively, and the subsequent TW network pattern above each (upper row). **c** Schematic indicating relevant parameters for the adapted dipole model assuming dipole excitation in the tip (${\alpha }_{t}$) as well as in the domains at either side of the TW (${\hat{\alpha }}_{I}$, ${\hat{\alpha }}_{{III}}$ in the example shown) according to their frequency-dependent permittivity tensor orientation (ellipsoids in I, III). **d** Calculated scattering signatures for the four types of TWs with maxima occurring for ${\mathrm{Re}}(\varepsilon_{\parallel /\perp })\approx -2$ (compare Fig. 2c) and contrast inversion apparent at $7.88\,\mathrm{THz}$.

In order to qualitatively discuss why different walls brighten or darken at different frequencies, the analytical point-dipole model commonly used to describe s-SNOM contrast was adapted^42–44,50^. Specifically, the model has been extended to treat a vertical interface (orthogonal to the surface) separating two adjacent LAO domains described by fully general permittivity tensors with arbitrarily oriented optical axes (for details see Suppl. Information S6). While an analytical treatment of an entire TWJ is highly non-trivial, modelling a single TW is found to be sufficient to explain the contrast of all TWs present in a single TWJ. We therefore apply the model to all TW types present on LAO(001) (Fig. 3a) and introduce the notation ${{TW}}_{v/d}^{\parallel /\perp }$, where v (vertical) and d (diagonal) denotes the wall direction. Moreover, the superscript ∥ (⊥) distinguishes the set of walls that appear bright at ${f}_{1}\,$ (${f}_{2}$) in the experiments as summarised in Fig. 3b, i.e. when the parallel (perpendicular) dielectric component, ${\varepsilon }_{\parallel }$ (${\varepsilon }_{\perp }$), matches the near-field resonance regime $\mathrm{Re}(\varepsilon )-3\; \mathrm{to}-1$^43,51^ (dashed lines in Fig. 2c and Supplementary Information S6). Since the (110) and $\left(1\bar{1}0\right)$ walls within one zig-zag set behave equivalently, we group them as ${{TW}}_{d}$. The grey arrows in Fig. 3a (optical-axis projections) then motivate two geometrical classes as illustrated by the top view representations: the ${\mathrm{TW}}^{\parallel }$ class (i.e. ${{TW}}_{v}^{\parallel }$ and ${{TW}}_{d}^{\parallel }$) with head-to-head configuration of the optical axes and the class of ${\mathrm{TW}}^{\perp }$ (i.e. ${{TW}}_{v}^{\perp }$ and ${{TW}}_{d}^{\perp }$) with tail-to-tail orientation (for details including side views and a 3D representation see SI S3). Consequently, TWJ_1_ consists of ${{TW}}_{v}^{\perp }$, two ${{TW}}_{d}^{\perp }$, and ${{TW}}_{v}^{\parallel }$ (the remaining junction compositions are listed in Suppl. Information S4). Moreover, Fig. 3b, c summarises which TW branches are bright at ${f}_{1}$ and ${f}_{2}$, respectively.

In the adapted analytical model, the near-field probe is represented by a small metallic sphere with isotropic polarizability ${\alpha }_{t}$, located at height h above the surface (exemplarily shown in Fig. 3c for ${{TW}}_{d}^{\perp }$ separating e.g. domains III and I). Moreover, each adjacent LAO domain is represented by an induced dipole, that is determined by the local anisotropic permittivity tensor and optical-axis orientation via its anisotropic polarizability tensor (e.g., ${\hat{\alpha }}_{I}$, ${\hat{\alpha }}_{{III}}$). For s-SNOM at the TW, it is the tip field that induces these dipoles in the two domains next to the TW–surface junction, resulting in a TW-type-dependent near-field coupling (see Suppl. Information S6 for details). Note that the polarizability tensors in the examined strongly dispersive regime of LAO are highly frequency dependent as indicated by the differently oriented ellipsoids in Fig. 3c. The resulting calculated scattering cross sections for all four TW types within RB_1_ consequently shows a highly frequency-dependent response (Fig. 3d), perfectly reproducing the experimentally and numerically observed contrast inversion: ${\mathrm{TW}}^{\parallel }$ exhibits a maximum at lower frequencies, while the ${\mathrm{TW}}^{\perp }$ signal is $\approx 25\%$ smaller, whereas at slightly higher frequencies the response reverses and ${\mathrm{TW}}^{\perp }$ becomes maximal, with the signal of ${{TW}}_{\parallel }$ being roughly 55% smaller. The spectral positions of these maxima in the model coincide with resonances of the tensorial domain polarizability components (${\alpha }_{\parallel }$, ${\alpha }_{\perp }$), resulting in a simple rule for observing an enhanced TW response: a wall appears bright when the dielectric component that points toward the TW-surface junction, i.e., $\mathrm{Re}\left({\varepsilon }_{\parallel }\right)$ or $\mathrm{Re}\left({\varepsilon }_{\perp }\right)$, matches the near-field resonance (Fig. 2c and Suppl. Information S6). Note that even though this simple analytical approximation is suitable to explain the spectral response of the TWs reasonably well, for a full theoretical description of the near-field images observed, including the TWJ and the adjacent domains, numerical simulations are required, such as the FEM-simulation carried out within this work. For more details on the adapted dipole model and its limitations, please see SI S6.

### Depth-resolved confinement and energy channelling

**Fig. 4:**
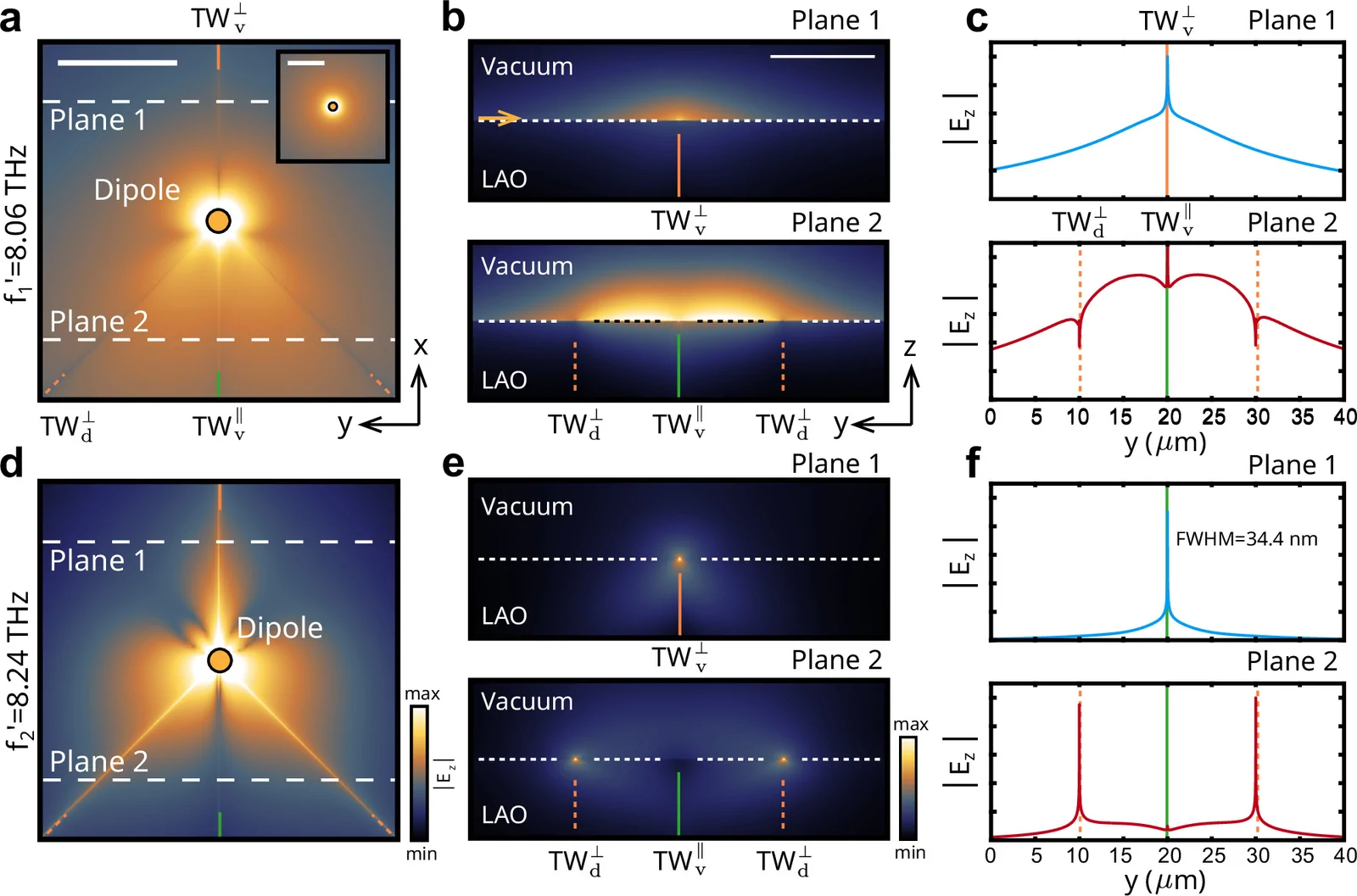
Numerical analysis of the optical confinement. **a**, **d** Electric field distribution at the interfaces of LAO TWs at (**a**) ${f}_{1}^{\prime} \approx 8.06\,\mathrm{THz}$ (${\lambda }_{1}^{\prime} \approx 37.17\,\upmu {\rm{m}}$, ${{\omega }_{1}}^{\prime}=\,269.0\,\mathrm{c}{\mathrm{m}}^{-1}$), and **d** ${f}_{2}^{\prime} \approx 8.24\,\mathrm{THz}$ (${\lambda }_{2}^{\prime} \approx 36.36\,\upmu {\rm{m}}$, ${{\omega }_{2}}^{\prime}=275.0\,\mathrm{c}{\mathrm{m}}^{-1}$). The images represent the el**e**ctric field distribution, $\left|{E}_{z}\right|$, in the XY plane, at $1\,\mathrm{nm}$ above the sample’s surface. Scale bar represents $10\,\upmu {\rm{m}}$. **b**, **e** Electric field distributions at ${f}_{1}^{\prime}$ (**b**) and ${f}_{2}^{\prime}$ (**e**) for YZ planes. Top panels represent a YZ cut taken along the top white dashed line in (**a**, **d**) (denoted as Plane 1) and bottom panels represent a YZ cut taken along the bottom white dashed line (denoted as Plane 2). Scale bar represents $10\,\upmu {\rm{m}}$. **c**, **f** Line profiles of the field distribution taken $1\,\mathrm{nm}$ above the sample’s surface, as denoted by the yellow arrow in (**b**) top panel, at ${f}_{1}^{\prime}$ (**c**) and ${f}_{2}^{\prime}$ (**f**). Top panels represent profiles taken along Plane 1 and bottom panels represent profiles taken along Plane 2. A clear effect of the twin boundary is observed on the electric field distribution yielding a theoretical field confinement of up to ${{\lambda }_{2}}^{\prime} / 1057$.

Finally, FEM using a fixed dipole position was applied to analyse how the near-field distribution evolves above and below the surface and to quantify the ultimate field confinement. At ${f}_{1}^{\prime}$ (Fig. 4a), the near-field distribution in the surface plane (XY plane) exhibits multi-lobe features within each domain due to the tilted and rotated optical axes, in clear contrast with an isotropic medium without domains (Fig. 4a inset), where no lobe features exist and the energy is isotropically radiated. Along ${\mathrm{TW}}_{d}^{\perp }$ the field concentrates near the junction but decays rapidly with distance, with $\left|{E}_{z}\right|$ values lower than those in the surrounding domains. Along ${\mathrm{TW}}_{v}^{\parallel }$ the field remains enhanced over larger distances, whereas ${\mathrm{TW}}_{v}^{\perp }$ shows suppression near the junction with only weak enhancement further away. Cross-sections in the YZ plane (Fig. 4b, indicated by the dashed lines termed Plane 1, for the upper panel, and Plane 2, for the lower panel, in Fig. 4a, taken $10\,\upmu {\rm{m}}$ away from the dipolar source) show that above the surface the field is broadly distributed with a localised hot spot at the TW position, while below the surface a similar pattern appears with much lower field penetration. Corresponding line profiles $1\,\mathrm{nm}$ above the surface (Fig. 4c, indicated by the yellow arrow in Fig. 4b upper panel) quantify the TW-bounded electric field confinement, manifested as peaks/dips at the TW positions superimposed on an otherwise broad background attributed to a surface PhP mode on the domains.

At ${f}_{2}^{\prime}$ (Fig. 4d), the behaviour changes qualitatively: the near field accumulates strongly along the $T{W}^{\perp }$ branches and remains highly localised along the walls for many tens of microns away from the excitation point, with negligible lateral spreading. YZ cross-sections (Fig. 4e, at Plane 1 and Plane 2, upper and lower panels, respectively) reveal intense and highly localised hot spots at the surface intersection of ${\mathrm{TW}}_{v}^{\perp }$ (upper panel) and at ${\mathrm{TW}}_{d}^{\perp }$, while ${\mathrm{TW}}_{v}^{\parallel }$ is suppressed (lower panel). Line profiles $1\,\mathrm{nm}$ above the surface (Fig. 4f) yield a FWHM of $34.4\,\mathrm{nm}$ (for the Plane 1 case, upper panel) at the intersection between ${\mathrm{TW}}_{v}^{\perp }$ and the bulk LAO surface, corresponding to an unprecedented lateral confinement of ${{\lambda }_{2}}^{\prime}/1057$ for ${{\lambda }_{2}}^{\prime}=\,36.36\,\upmu {\rm{m}}$, and similarly narrow maxima at ${\mathrm{TW}}_{d}^{\perp }$ (Plane 2, lower panel). Note that, this ultimate limit is inaccessible in the s-SNOM experiment, due to the limitation imposed by the finite size of the probe.

## Discussion

In summary, we demonstrate experimentally and theoretically that ferroelastic twin walls in LaAlO_3_ provide a pattern-free and intrinsically reconfigurable platform for concentrating and routing THz–MIR near fields at the nanometre length scale at room temperature. The broken translational symmetry at a given TW, combined with the intrinsic optical anisotropy of LAO enables selected walls to act as confinement channels of the near field. By tuning the illumination frequency, the TW contrast switches in a reproducible manner, effectively turning specific branches of the naturally occurring TW network “on” or “off” and directing highly confined near-field signals along predefined crystallographic directions.

Quantitatively, an experimental lateral confinement factor of 260 ($\mathrm{FWHM}\approx 143\,\mathrm{nm}$ at ${\lambda }_{2}=\,37.17\,\upmu {\rm{m}}$) was observed, while finite-element modelling predicts confinement down to $34.4\,\mathrm{nm}$, corresponding to ${{\lambda }_{2}}^{\prime}/1057$ at ${{\lambda }_{2}}^{\prime}=\,36.36\,\upmu {\rm{m}}$, at selected TWs. Crucially, the TW-bound near field remains highly localised along the walls over tens of micrometres ‒ i.e., over mesoscopic distances that exceed the lateral confinement by more than three orders of magnitude ‒ while exhibiting negligible lateral spreading. This combination bridges long-range, guided near-field transport with deep-subwavelength lateral modal confinement in an unpatterned bulk dielectric.

More broadly, ferroelastic twinning is widespread and offers multiple external control knobs (e.g., mechanical strain, temperature and, in ferroelectrics, electrical control), suggesting routes toward dynamically reconfigurable THz/MIR near-field routing. Integrating ferroelastic TW networks with low-loss hyperbolic polaritonic materials (e.g., hBN, a-MoO_3_ or V_2_O_5_) could extend the concept toward compact polaritonic circuitry and enhanced nanospectroscopy/nanochemistry based on strongly confined, directional near fields. Furthermore, LAO is compatible with established oxide heteroepitaxy, including growth on STO/Silicon platforms and integration with oxide functional overlayers, offering a realistic route to embed crystallographically defined twin-wall polaritonic channels into oxide-electronics and silicon-compatible nanophotonic devices

## Methods

### Sample description

The samples studied within this work are (001)-grown bulk LAO crystals by Crystal GmbH, Germany. On LAO(001), four domains are naturally formed with a rhombohedral angle between sub-facets of only $\approx 0.075^\circ$, so the surface is effectively flat. Additionally, the overall crystallographic angles can be suppressed by a further polishing process, which was performed on all samples measured in this study. The exact sample area investigated within this work was precharacterised by AFM and Raman-measurements, enabling the determination of the exact axis orientation and domain identification.

### Fourier-transform infrared spectroscopy

Polarised Fourier-transform infrared spectroscopy (FTIR) was employed to characterise the MIR-THz optical response of a (110)-grown bulk LAO substrate. FTIR spectra were collected by means of a bolometer detector (frequency range from 120 to $600\,\mathrm{c}{\mathrm{m}}^{-1}$) and a MCT detector (frequency range from 500 to $7000\,\mathrm{c}{\mathrm{m}}^{-1}$), employing a globar as a broadband light source. A layer of $100\,\mathrm{nm}$ of Au was deposited on part of the LAO substrate and used as reference for normalising the near-field signal. The angle of incidence of the incoming light on the sample was approximately $15^\circ$. For details on the permittivity extraction see Suppl. Information S2.

### Scattering-type scanning near-field optical microscopy

Scattering-type scanning near-field optical microscopy (s-SNOM) measurements were performed by means of commercially available neaSCOPE s-SNOM systems (Attocube Systems GmbH). The systems employed either the radiation from a synchrotron (SINS)^52^ or from a free-electron laser^42^ as excitation light sources in the spectral regimes of 40−2000 cm^−1^ or $5-250\,\upmu {\rm{m}}$, respectively.

The SINS measurements were carried out at the Advanced Light Source, ALS, at Lawrence Berkeley National Laboratory on a (001)-grown LAO crystal and were used to demonstrate surface PhP launching and to extract the dispersion (see Suppl. Information S5). SINS is based on the s-SNOM technique and provides nanoscale-resolved spectra with wavelength-independent spatial resolution. In this case, we have employed a lithograpically-defined Au bar as polaritonic launcher. We acquired third-harmonic SINS line scans perpendicular to the Au bar (see Suppl. Information Fig. S13), which reveal enhanced near-field signal and sets of interference fringes within RB1 and RB2 (RB3 fringes are not resolved due to higher intrinsic losses), with frequency-dependent fringe period. We attribute this oscillations to the excitation of non-strongly confined surface PhPs at the surface of bulk LAO(001) (see Supplementary Information S5 for a full discussion).

FEL-SNOM has been carried out using the pulsed quasi-cw free-electron laser FELBE at the Helmholtz-Zentrum Dresden Rossendorf (HZDR), covering the full frequency range from 1.2 to 60 THz with 13 MHz repetition rate, a pulse length between 1 and 25 ps and a bandwidth of 0.2 to 1%^42^. Metal coated (Pt-Ir) tips with a fundamental mechanical resonance frequency of approximately $250\,\mathrm{kHz}$ and tapping amplitude of approximately $100\,\mathrm{nm}$ were used. The detected signals were demodulated at the second harmonic of the tip tapping frequency to suppress far-field contributions. Backscattered signals were collected using either a LHe-cooled Ge:Ga photodetector or a LN-cooled MCT detector depending on the spectral regime. Given the comparatively low signal-to-noise ratio of the FEL light source due to intensity instabilities, a self-homodyne detection scheme was adopted.

### Finite Element Method Simulations

Finite element method (FEM) simulations of the near-field optical response of bulk LAO were performed using the Wave Optics module of COMSOL Multiphysics. The illuminated AFM tip is modelled as a vertically-oriented electric point dipole, placed $2\,\upmu {\rm{m}}$ above the LAO surface. To mimick the SNOM measurements, a point probe was placed $1\,\mathrm{nm}$ above the LAO surface and both dipole and probe points were raster-scanned over a $30\,\upmu {\rm{m}} \times 30\,\upmu {\rm{m}}$ lateral (x,y) area. The amplitude, $\left|{E}_{z}\right|$, and phase, $\varPhi \left({E}_{z}\right)$, of the out-of-plane component of the electric field were recorded as a function of the (x,y) dipole and probe positions. For the simulations with a fixed dipole position, a snapshot of the $\left|{E}_{z}\right|$ field distribution is taken $1\,\mathrm{nm}$ above the LAO sample’s surface. We use as an input the dielectric permittivity extracted from the FTIR measurements. In the simulated area, domain “II” has its optical axis aligned along [111], and domains III, IV, and I correspond to successive $90^\circ$ anticlockwise rotations (labels in Fig. 2e).

## Supplementary information

Supplementary Information

Transparent Peer Review file

## Data Availability

All data supporting the findings of this study are available in the Article and the Supplementary Information. Additional data can be obtained from the corresponding authors upon reasonable request.
