# Supplementary material for: Ultra-high THz-field-confinement at LaAlO3 twin walls
Source: Nat Commun. 2026 Sep 28;17:11212. doi: 10.1038/s41467-026-77689-5 (PMC13720113; doi:10.1038/s41467-026-77689-5)
Supplement: Supplementary file 1 [file 41467_2026_77689_MOESM1_ESM.pdf]

Supplementary Information for:

Ultra-high THz-field-confinement at LaAlO3 twin walls

J. Wetzel[1,2*], J. Taboada-Gutiérrez[3*a], M. Roeper[1], F.G. Kaps[1], G. Esposito[3], D. Marchese[3], R. Buschbeck[1], P. Lenz[1], J.M. Klopf[4], H.A. Bechtel[5], S.N. Gilbert Corder[5], J. Teyssier[3], S.C. Kehr[1], L.M. Eng[1,2], A.B. Kuzmenko[3], and S.D. Seddon[1a]

[1]Institute of Applied Physics, TUD Dresden University of Technology, Nöthnitzer Straße 61, 01187 Dresden, Germany
[2]Würzburg-Dresden Cluster of Excellence (EXC2147) ctd.qmat – Complexity, Topology, and Dynamics in Quantum Matter, Dresden, Germany
[3]University of Geneva, Department of Quantum Matter Physics (DQMP), Geneva 1211, Switzerland
[4]Institute of Radiation Physics, Helmholtz-Zentrum Dresden Rossendorf, 01328 Dresden, Germany
[5]Advanced Light Source (ALS), Lawrence Berkeley National Laboratory, Berkeley, CA 94720, USA

*These authors contributed equally
[a]email: samuel.seddon@tu-dresden.de, and javier.taboadagutierrez@unige.ch

Supplementary Section S1: On the effective permittivity for the (100) and (010) planes

As within our work the near-field signal on a LAO(001) surface is investigated, but the permittivity tensor is aligned with the optical axes along the crystallographic [111] direction and its equivalent rotations, here the effective permittivity on the LAO(001) surface for two exemplary domains is calculated. For a uniaxial crystal whose optical axis lies along the direction $\vec{n}$, the permittivity is given by the following relationship:

$$\hat{\varepsilon}=\varepsilon_{\perp}\hat{I}+(\varepsilon_{\parallel}-\varepsilon_{\perp})\vec{n}\otimes\vec{n}^{T} \quad (1)$$

With $\otimes$ the dyadic product.

In the case of domain I, $\vec{n}$ is given by:

$$\vec{n}_{\mathrm{I}}=\frac{1}{\sqrt{3}}\begin{pmatrix}1\\-1\\1\end{pmatrix} \quad (2)$$

And, as a consequence,

$$\vec{n}_{\mathrm{I}}\otimes\vec{n}_{\mathrm{I}}^{T}=\frac{1}{3}\begin{pmatrix}1&-1&1\\-1&1&-1\\1&-1&1\end{pmatrix} \quad (3)$$

So:

$$\hat{\varepsilon}_{\mathrm{I}}=\varepsilon_{\perp}\begin{pmatrix}1&0&0\\0&1&0\\0&0&1\end{pmatrix}+\frac{(\varepsilon_{\parallel}\text{-}\varepsilon_{\perp})}{3}\begin{pmatrix}1&-1&1\\-1&1&-1\\1&-1&1\end{pmatrix}=\frac{1}{3}\begin{pmatrix}\varepsilon_{\parallel}+2\varepsilon_{\perp}&-(\varepsilon_{\parallel}\text{-}\varepsilon_{\perp})&\varepsilon_{\parallel}\text{-}\varepsilon_{\perp}\\-(\varepsilon_{\parallel}\text{-}\varepsilon_{\perp})&\varepsilon_{\parallel}+2\varepsilon_{\perp}&-(\varepsilon_{\parallel}\text{-}\varepsilon_{\perp})\\\varepsilon_{\parallel}\text{-}\varepsilon_{\perp}&-(\varepsilon_{\parallel}\text{-}\varepsilon_{\perp})&\varepsilon_{\parallel}+2\varepsilon_{\perp}\end{pmatrix} \tag{4}$$

Which is the shape the dielectric tensor takes when written in the laboratory frame given by the orthonormal vectors [100], [010], and [001]. Therefore, the (001) plane projection will be given by:

$$\hat{\varepsilon}_{\mathrm{I}(001)}=\begin{pmatrix}\varepsilon_{xx}&\varepsilon_{xy}\\\varepsilon_{yx}&\varepsilon_{yy}\end{pmatrix}=\frac{1}{3}\begin{pmatrix}\varepsilon_{\parallel}+2\varepsilon_{\perp}&-(\varepsilon_{\parallel}\text{-}\varepsilon_{\perp})\\-(\varepsilon_{\parallel}\text{-}\varepsilon_{\perp})&\varepsilon_{\parallel}+2\varepsilon_{\perp}\end{pmatrix}. \tag{5}$$

Note that, for domain III we find the exact same result, but with the tensor elements ε_{xz}, $\varepsilon_{yz,}$ ε_{zx}, and ε_{zy} multiplied by -1.

In the case of domain II, $\vec{n}$ is given by:

$$\vec{n}_{\mathrm{II}}=\frac{1}{\sqrt{3}}\begin{pmatrix}1\\1\\1\end{pmatrix} \tag{6}$$

And, as a consequence,

$$\overrightarrow{n_{\mathrm{II}}}\otimes\overrightarrow{n_{\mathrm{II}}}^{T}=\frac{1}{3}\begin{pmatrix}1&1&1\\1&1&1\\1&1&1\end{pmatrix} \tag{7}$$

So:

$$\hat{\varepsilon}_{\mathrm{II}}=\varepsilon_{\perp}\begin{pmatrix}1&0&0\\0&1&0\\0&0&1\end{pmatrix}+\frac{(\varepsilon_{\parallel}\text{-}\varepsilon_{\perp})}{3}\begin{pmatrix}1&1&1\\1&1&1\\1&1&1\end{pmatrix}=\frac{1}{3}\begin{pmatrix}\varepsilon_{\parallel}+2\varepsilon_{\perp}&\varepsilon_{\parallel}\text{-}\varepsilon_{\perp}&\varepsilon_{\parallel}\text{-}\varepsilon_{\perp}\\\varepsilon_{\parallel}\text{-}\varepsilon_{\perp}&\varepsilon_{\parallel}+2\varepsilon_{\perp}&\varepsilon_{\parallel}\text{-}\varepsilon_{\perp}\\\varepsilon_{\parallel}\text{-}\varepsilon_{\perp}&\varepsilon_{\parallel}\text{-}\varepsilon_{\perp}&\varepsilon_{\parallel}+2\varepsilon_{\perp}\end{pmatrix}. \tag{8}$$

Here, the (001) plane projection is given by:

$$\hat{\varepsilon}_{\mathrm{II}(001)}=\begin{pmatrix}\varepsilon_{xx}&\varepsilon_{xy}\\\varepsilon_{yx}&\varepsilon_{yy}\end{pmatrix}=\frac{1}{3}\begin{pmatrix}\varepsilon_{\parallel}+2\varepsilon_{\perp}&\varepsilon_{\parallel}\text{-}\varepsilon_{\perp}\\\varepsilon_{\parallel}\text{-}\varepsilon_{\perp}&\varepsilon_{\parallel}+2\varepsilon_{\perp}\end{pmatrix}. \tag{9}$$

Similar to domains I and III, again domain IV shows the same result as domain II, just with the tensor elements ε_{xz}, $\varepsilon_{yz,}$ ε_{zx}, and ε_{zy} multiplied by -1.

The (001)-plane projection $\hat{\varepsilon}_{(001)}$ in both cases describes an ellipse (see SI S7 Fig. S17) with its principal axes aligned along 45° in the surface grid, and its orientation in the case of domains I and III rotated by 90° compared to the ellipse of domain II and IV, shown by the flipped sign of the off-diagonal elements of $\hat{\varepsilon}_{(001)}$. However, this in-plane projection ignores all elements with a z-component, which are necessary to fully explain the observed contrast behaviour at the TWs.

Supplementary Section S2: Extraction of the IR permittivity of $LaAlO_3$(001)

To extract the infrared (IR) permittivity of $LaAlO_3$ (LAO), we employed a Bruker Vertex v70 Fourier Transform Infrared Spectrometer (FTIR), coupled to a Bruker Hyperion 2000 microscope that allows us to exchange between different objectives. We used a 15x objective with a numerical aperture (NA) of 0.4, corresponding to an average incidence angle of $\approx 13°$, and measured in reflection mode. In our system, light from a broadband IR source (globar) is modulated by a Michelson interferometer and then focused on the sample by means of a Cassegrain objective. Cassegrain objectives consist of a primary concave parabolic mirror and a secondary convex hyperbolic mirror which make them a versatile tool for broad wavelength applications (such as FTIR) as they are based on reflection of light instead of refraction, avoiding chromatic aberration of light and ensuring a constant focal length of the objective across all IR wavelengths. Then, the reflected light is directed to the detector by the Cassegrain objective again. Furthermore, the microscope allows us to control the polarisation of light both before and after illuminating the sample. In our measurements, a MCT detector for mid-infrared (MIR) frequencies (580 to 8000 cm^{-1}) and a bolometer for far-infrared (FIR) frequencies (120 to 700 cm^{-1}) were employed. Figure S1 shows a sketch of the whole spectroscopic system, labelling all optical components along the light beam path and highlighting the interferometer, Cassegrain objective and light sources and detector.

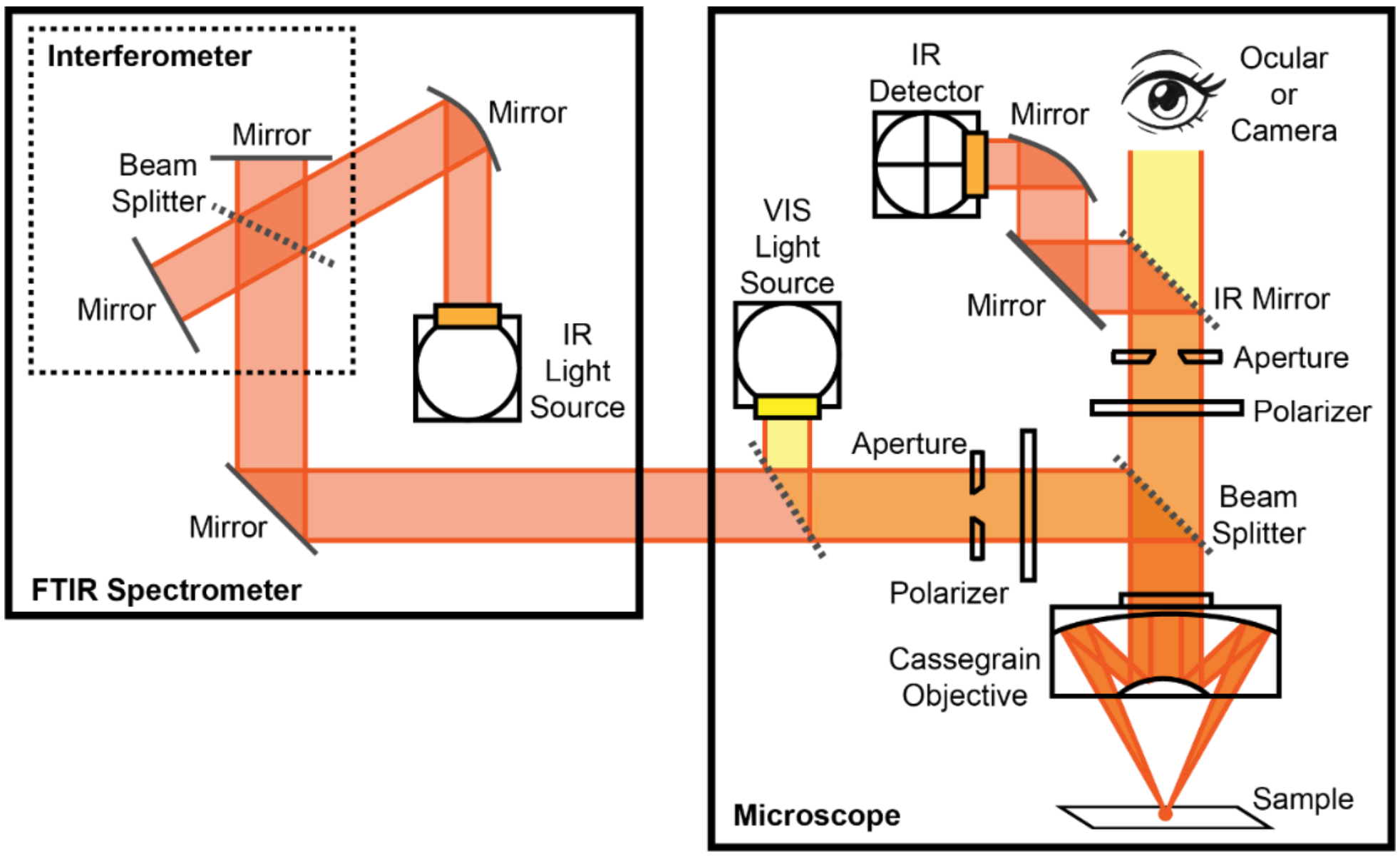

Figure S1. Schematics of the FTIR system coupled to a microscope. Light from an IR source (typically a globar) is modulated by means of a Michelson interferometer and then focused onto sample via a Cassegrain objective. The reflected light is collected again by the same Cassegrain objective and directed to the detector (MCT or bolometer) thanks to a set of mirrors. Furthermore, the setup provides us with precise control of light polarisation and incidence angle.

$LaAlO_3$ (LAO) is a distorted perovskite oxide, with R-3c space group, which in its rhombohedral phase, has cell parameters are given by $a=b=c=5.38552$Å and $\alpha=\beta=\gamma=60.0109°$. LAO is a wide-bandgap insulator ($E_g \approx 5.6eV$), and therefore, in the IR regime, contributions to the optical conductivity are expected only from phonon excitations. In particular, LAO is a uniaxial optical material, which means that its permittivity matrix is given by the following tensor:

$$\hat{\varepsilon}=\begin{pmatrix}\varepsilon_{\perp} & 0 & 0\\ 0 & \varepsilon_{\perp} & 0\\ 0 & 0 & \varepsilon_{\parallel}\end{pmatrix}, \tag{10}$$

where $\varepsilon_{\perp}$ is the permittivity component perpendicular to the optical axis and $\varepsilon_{\parallel}$ the dielectric component parallel to the optical axis. In the case of LAO, the optical axis is oriented with the [111] crystallographic direction for the rhombohedral phase (in the case of the hexagonal representation it would be aligned with the [001] direction). Consequently, the extraction of the full dielectric constant tensor of the material will require to perform polarisation-resolved reflectivity measurements. Ideally, a crystal grown along the [111] direction would simplify measurements and data analysis, but such crystals exhibit twinned domains with random sizes typically ranging from 30 to 60 μm in length, which means that FIR measurements will have undesired contributions to the overall reflectivity coming from adjacent domains, even for the smallest spot sizes achievable with our microscope, which are around 100x100 μm^2 (limited by diffraction effects in FIR frequencies). Instead, we used a monodomain LAO sample grown along the [110] direction with a polished surface. Nevertheless, in this sample, the optical axis is tilted relative to the surface, resulting in a permittivity tensor given by:

$$\hat{\varepsilon}=\begin{pmatrix}\varepsilon_{\perp}cos^2\alpha_{oa}+\varepsilon_{\parallel}sin^2\alpha_{oa} & 0 & (\varepsilon_{\perp}\text{-}\varepsilon_{\parallel})cos\alpha_{oa}sin\alpha_{oa}\\ 0 & \varepsilon_{\perp} & 0\\ (\varepsilon_{\perp}\text{-}\varepsilon_{\parallel})cos\alpha_{oa}sin\alpha_{oa} & 0 & \varepsilon_{\parallel}cos^2\alpha_{oa}+\varepsilon_{\perp}sin^2\alpha_{oa}\end{pmatrix}, \tag{11}$$

with α_{oa}=35.1548° the angle that forms the direction of the optical axis ([111] direction) with the surface normal ([110] direction), determined from the crystal structure. This tilt complicates permittivity extraction, as the optical axis projection on the XY plane (sample surface) introduces an azimuthal angle γ_{oa} which is initially unknown. Figure S2 sketches the geometry of our system, showing the incidence and polarisation angles for the incident light (θ_i and ϕ_i, respectively) and the zenith and azimuthal angles for the optical axis (α_{oa} and γ_{oa}, respectively).

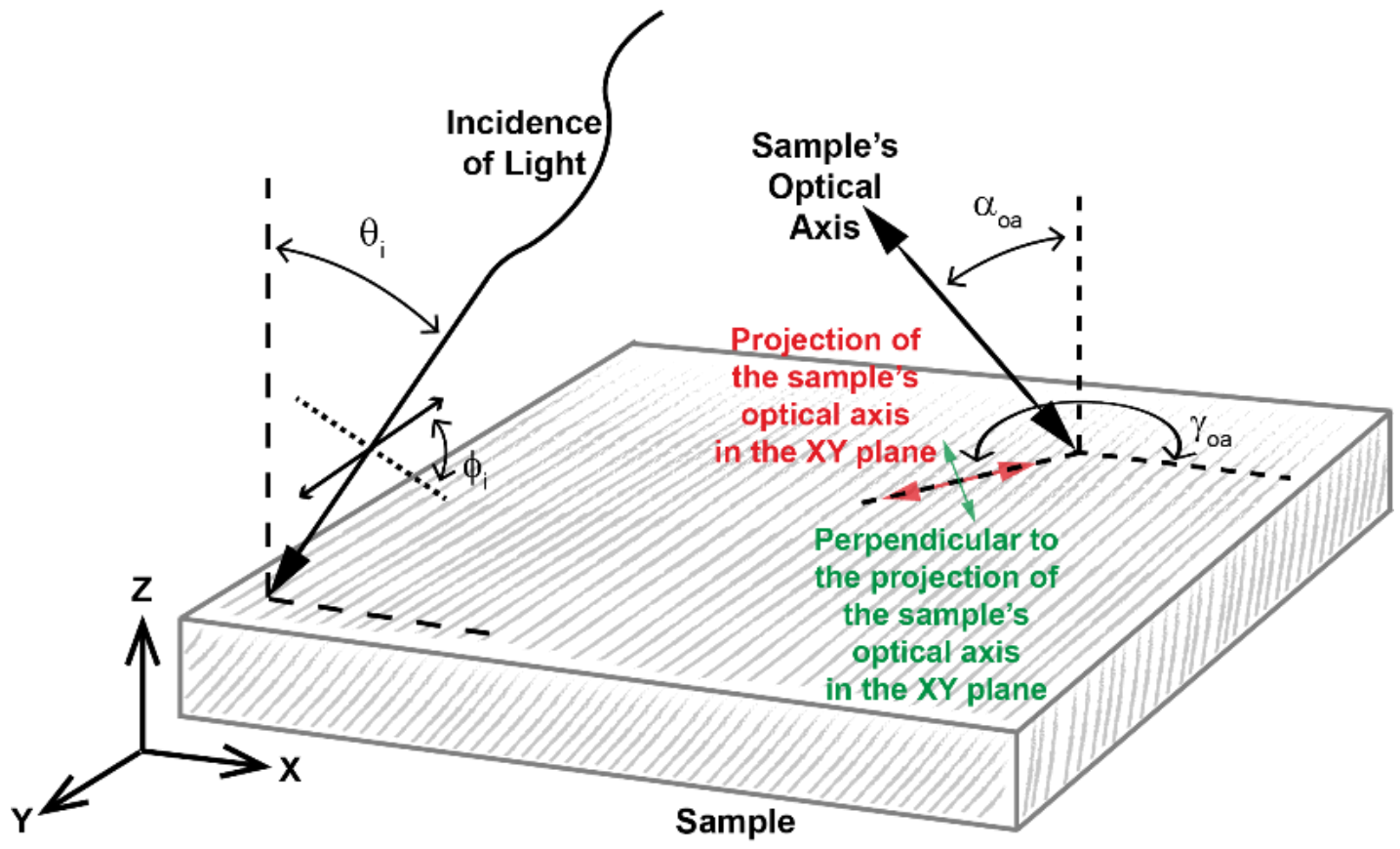

Figure S2. Schematics of the incidence of light on our sample. Incident light with angle θ_i and polarisation angle ϕ_i illuminates the sample. The sample is characterized by its tilted uniaxial permittivity tensor, $\hat{\varepsilon}$. The optical axis, tilted at angle α_{oa}=35.1548° relative to the surface normal, has an azimuthal angle, γ_{oa}, in the XY plane. The red and green arrows indicate the directions parallel and perpendicular to the optical axis projection, respectively.

This situation, although inconvenient, is not unmanageable. Our first goal is to measure reflectivity along the optical axis projection on the XY plane and its perpendicular direction (stressed by the red and green arrows in the sketch of Figure S2, respectively). Reflectivity measured with the electric field oriented parallel to the optical axis projection on the XY plane

(red arrow in Figure S2) will give us a reflectivity that is a function of both $\varepsilon_\perp$ and $\varepsilon_\parallel$, $R_{pr}=R_{pr}(\varepsilon_\perp,\varepsilon_\parallel)=R_{pr}(\varepsilon_{eff})$, due to the tilting of the optical axis, whereas reflectivity measured for the electric field oriented along the direction perpendicular to that projection will give us directly a reflectively which is purely coming from the $\varepsilon_\perp$ component, $R_\perp=R_\perp(\varepsilon_\perp)$. Since the azimuthal angle of the optical axis γ_{oa} is unknown and we cannot align the polarisation of light with the optical axis projection, we measured reflectivity at four random polarisation angles ϕ_i we called $0°$, $45°$, $90°$ and -$45°$, as the reflectivity for any polarisation angle ϕ_0 can be calculated from any set of 3 independent reflectivity measurements [1]:

$$R(\phi_0,\omega)=aR(\phi_1,\omega)+bR(\phi_2,\omega)+cR(\phi_3,\omega) \quad (12)$$

With ϕ_1, ϕ_2 and ϕ_3 the polarisation angles for each measurement, γ the polarisation angle at which we want to extract the reflectivity spectrum and a, b and c given by:

$$a=\frac{sin(\phi\text{-}\phi_2)sin(\phi\text{-}\phi_3)}{sin(\phi_1\text{-}\phi_2)sin(\phi_1\text{-}\phi_3)}$$
$$b=\frac{sin(\phi\text{-}\phi_1)sin(\phi\text{-}\phi_3)}{sin(\phi_2\text{-}\phi_1)sin(\phi_2\text{-}\phi_3)} \quad (13)$$
$$c=\frac{sin(\phi\text{-}\phi_1)sin(\phi\text{-}\phi_2)}{sin(\phi_3\text{-}\phi_1)sin(\phi_3\text{-}\phi_2)},$$

Figure S3 shows the reflectivity spectra at these four polarisations, revealing three high-reflectivity bands associated with phonon modes (the so-called Reststrahlen bands, RBs) between approximately 150 and 280 cm^{-1}, 430 and 600 cm^{-1}, and 650 and 780 cm^{-1}. The largest differences occur near 592 cm^{-1} and in the third RB.

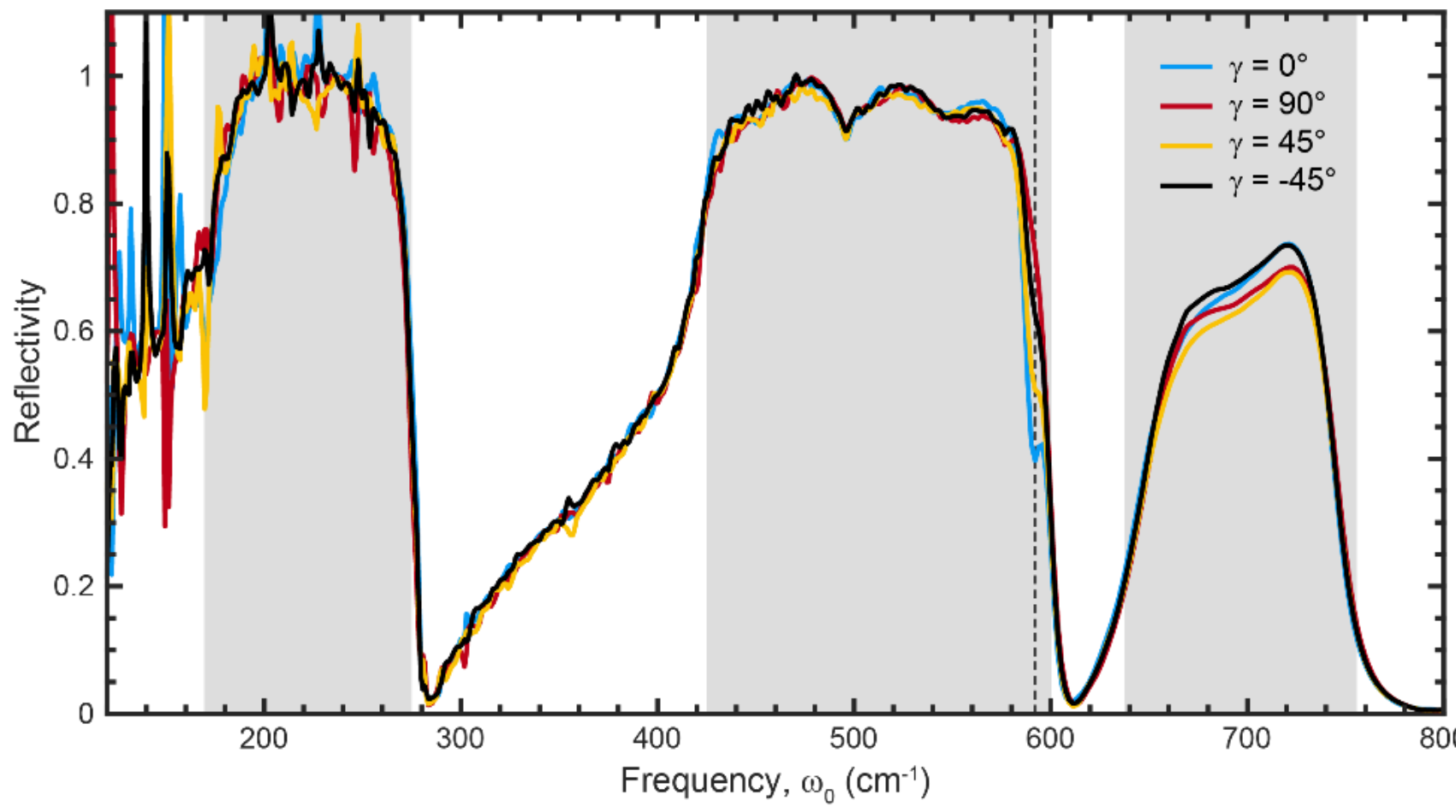

Figure S3. IR reflectivity spectra of LAO at four polarisation angles. Spectra at $0°$ (blue curve), $90°$ (red line), $45°$ (yellow curve) and -$45°$ (black line) identify 3 RBs, stressed by grey shaded regions, a first one between ≈ 150 and 280 cm^{-1}, a second one from ≈ 430 to 600 cm^{-1}, and a third one between ≈ 640 and 760 cm^{-1}. We observe the largest differences between all polarisations close to the ω_{LO} phonon at ≈ 592 cm^{-1} (vertical dashed line) and in the third RB.

To verify Equation 12, we have intentionally used the spectra at $\phi_i=0°$, $90°$ and $45°$ to recover the reflectivity spectrum at ϕ_i=-$45°$. Figure S4 compares the calculated and measured spectra, showing excellent agreement.

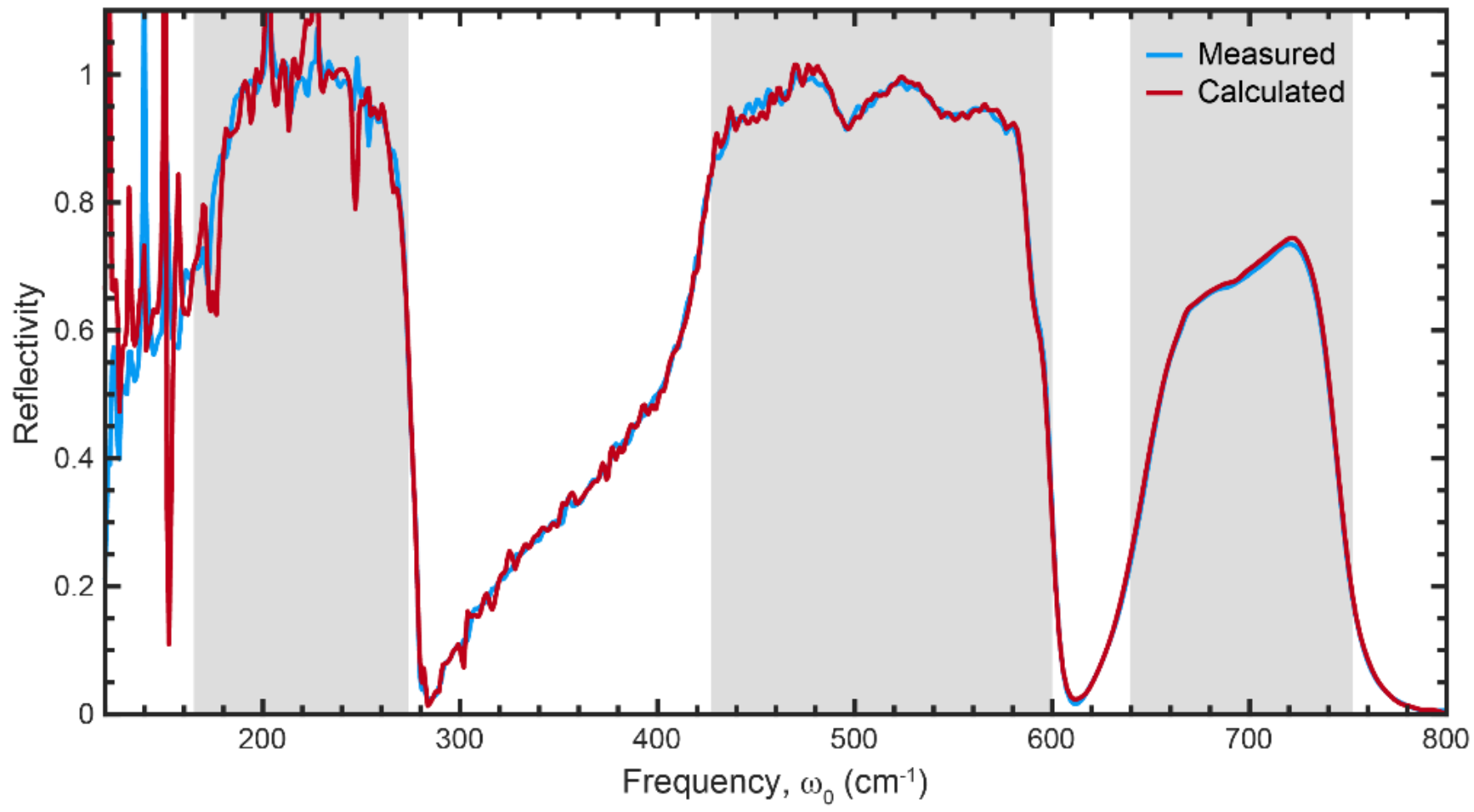

Figure S4. Validation of polarisation reconstruction. The LAO measured reflectivity spectrum at ϕ_i=-45° is compared with the spectrum calculated employing 12 and the spectra at ϕ_i=0°, 90° and 45°. The calculated spectrum (red line) is fully in line with the measured one (blue curve). RBs are stressed by grey shaded regions.

For a certain frequency, the largest reflectivity spectral contrast occurs between polarisations aligned along and across the optical axis projection and therefore, employing Equation 12 and scanning for all possible polarisation angles at a given frequency, we can identify at which polarisation angles the difference becomes maximum. We have selected ω_0=592 cm^{-1} (vertical dashed line in Figure S3) as the difference in reflectivity is maximum between all measured polarisations, see Figure S5.

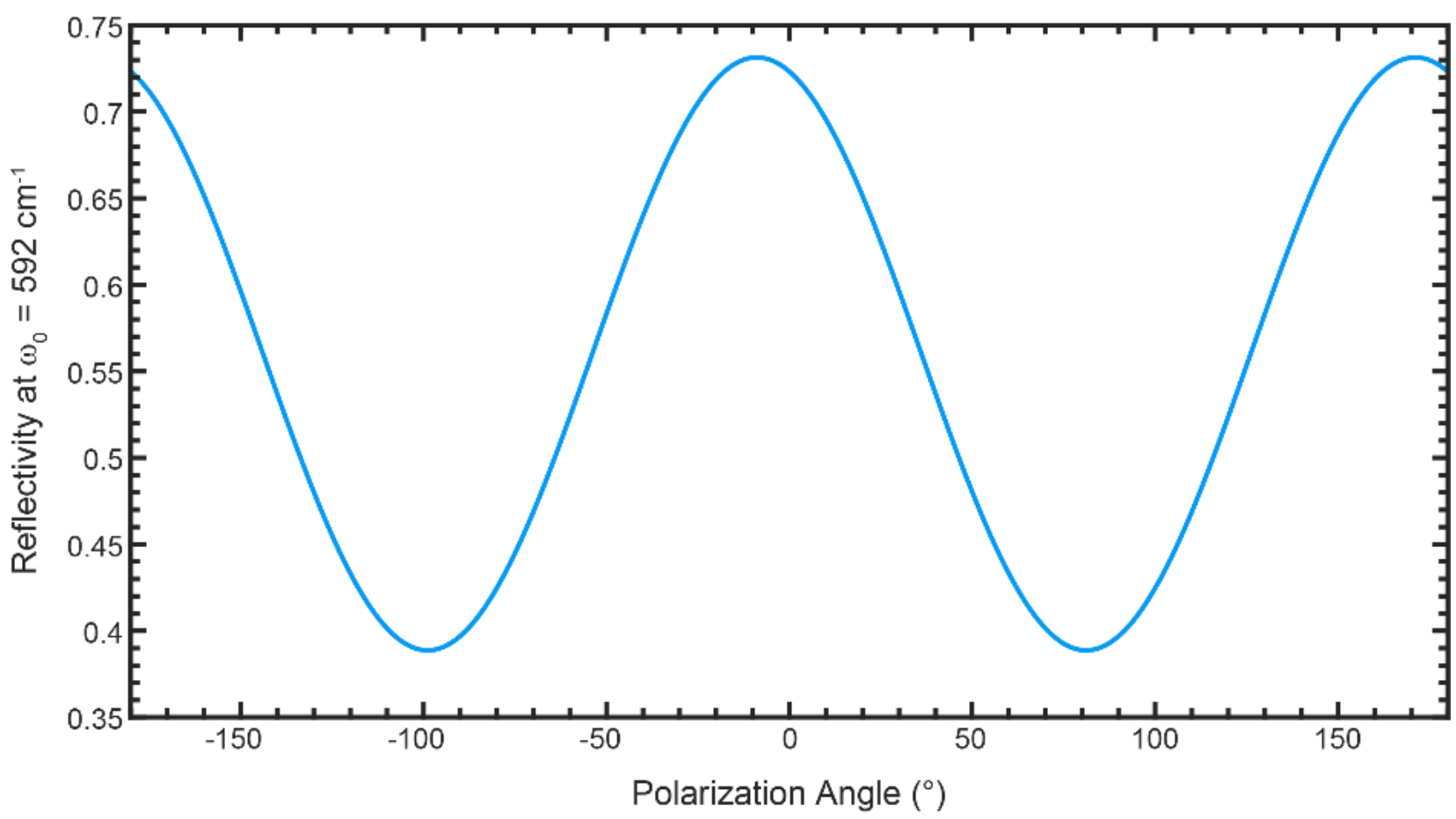

Figure S5. Reflectivity at ω_0=592 cm^{-1} as a function of polarisation angle, showing sinusoidal behaviour with a 180° periodicity. From the minimum (ϕ_i=81°) and maximum (ϕ_i=-9°) positions, we can calculate the reflectivity spectra for the directions parallel and perpendicular to the optical axis projection in the XY plane.

We find a sinusoidal curve with a periodicity equal to 180°, with a maximum at ϕ_i=-9° and a minimum at ϕ_i=81°. Consequently, we have calculated the whole IR reflectivity spectra employing Equation 12 for those 2 polarisation angles, shown in Figure S6. The spectrum at ϕ_i=81° (red curve) reflects a coupling between the two permittivity components $\varepsilon_\perp$ and $\varepsilon_\parallel$ (R_{min}=$R_{min}(\varepsilon_\perp, \varepsilon_\parallel)$=$R_{min}(\varepsilon_{eff})$,) that can be identified thanks to the kink which appears close to the edge of the second RB at around 590 cm^{-1}. If that reflectivity would have come from a pure dielectric component, it would not have shown any kink, and the spectra would have decayed

stepless as it is indeed shown in the other curve that corresponds to ϕ_i=-9° and that we can associate with $R_{max}=R_{\perp}(\varepsilon_{\perp})$.

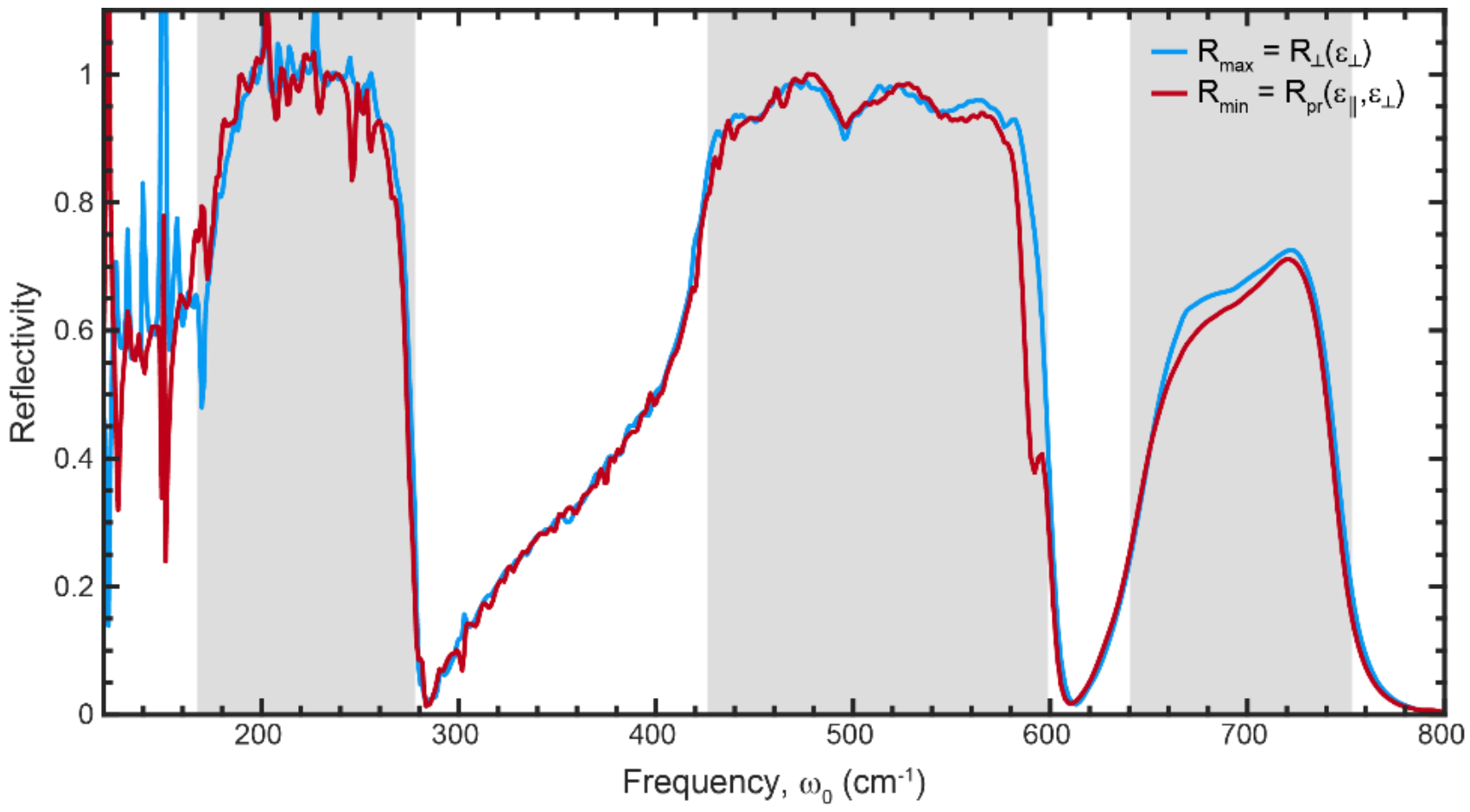

Figure S6. Reflectivity spectra at maximum and minimum polarisation angles obtained in Figure S5. The reflectivity spectrum at ϕ_i=81° (red curve, $R_{min}=R_{pr}(\varepsilon_{\parallel}, \varepsilon_{\perp})$) shows a mixed contribution between $\varepsilon_{\parallel}$ and $\varepsilon_{\perp}$, while the spectrum calculated at ϕ_i=-9° (blue curve, $R_{max}=R_{\perp}(\varepsilon_{\perp})$) shows a pure contribution from $\varepsilon_{\perp}$. RBs are depicted by grey shaded regions.

We can directly fit R_{max} to extract $\varepsilon_{\perp}$. $\varepsilon_{\parallel}$ on the other hand, can be extracted by supposing that the optical response of the $R_{min}=R_{min}(\varepsilon_{mix})$ curve is given by an effective permittivity that mixes both $\varepsilon_{\parallel}$ and $\varepsilon_{\perp}$ [2]:

$$\varepsilon_{mix}=\varepsilon_{\infty,mix}+\frac{(\varepsilon_{\perp}-\varepsilon_{\infty,\perp})cos^2\alpha_{oa}+(\varepsilon_{\parallel}-\varepsilon_{\infty,\parallel})sin^2\alpha_{oa}+(\varepsilon_{\parallel}-\varepsilon_{\infty,\parallel})(\varepsilon_{\perp}-\varepsilon_{\infty,\perp})}{1+(\varepsilon_{\perp}-\varepsilon_{\infty,\perp})sin^2\alpha_{oa}+(\varepsilon_{\parallel}-\varepsilon_{\infty,\parallel})cos^2\alpha_{oa}}, \quad (14)$$

With $\varepsilon_{\infty,mix}$, $\varepsilon_{\infty,\parallel}$, $\varepsilon_{\infty,\perp}$ the high-frequency permittivities for the "effective mixed" state, parallel and perpendicular components of the dielectric tensor. We fitted the reflectivity spectra employing RefFit, a software for fitting optical spectra with a wide variety of dielectric-function models [3], setting α_{oa}=35°, which is the crystallographic angle that the [110] and [111] directions form in LAO. Both $\varepsilon_{\perp}$ and $\varepsilon_{\parallel}$ have been modelled employing a Drude-Lorentz oscillators model:

$$\varepsilon_i(\omega)=\varepsilon_{\infty,i}+\sum_n \frac{\omega_{p,i,n}^2}{\omega_{,i,n}^2-\omega^2-i\gamma_{i,n}\omega}, \quad (15)$$

With i= ⊥ or ∥, $\varepsilon_{\infty,i}$ the high-frequency permittivity for the i component of the dielectric tensor and $\omega_{,i,n}$, $\omega_{p,i,n}$ and $\gamma_{i,n}$ the transversal optical frequency, "plasma" frequency and damping factor of the n-th mode, respectively. We have employed 5 modes in both cases. Figure S7 shows the fittings to the reflectivity R_{max} and R_{min} spectra. Table S1 summarizes the fitted parameters for both $\varepsilon_{\parallel}$ and $\varepsilon_{\perp}$. For simplicity, and because the pseudocubic nature of the material leads to only a small frequency shift between the $\varepsilon_{\parallel}$ and $\varepsilon_{\perp}$ dielectric components, we refer to the Reststrahlen bands as RB1, RB2, and RB3. Strictly speaking, however, one should further differentiate between the permittivity components.

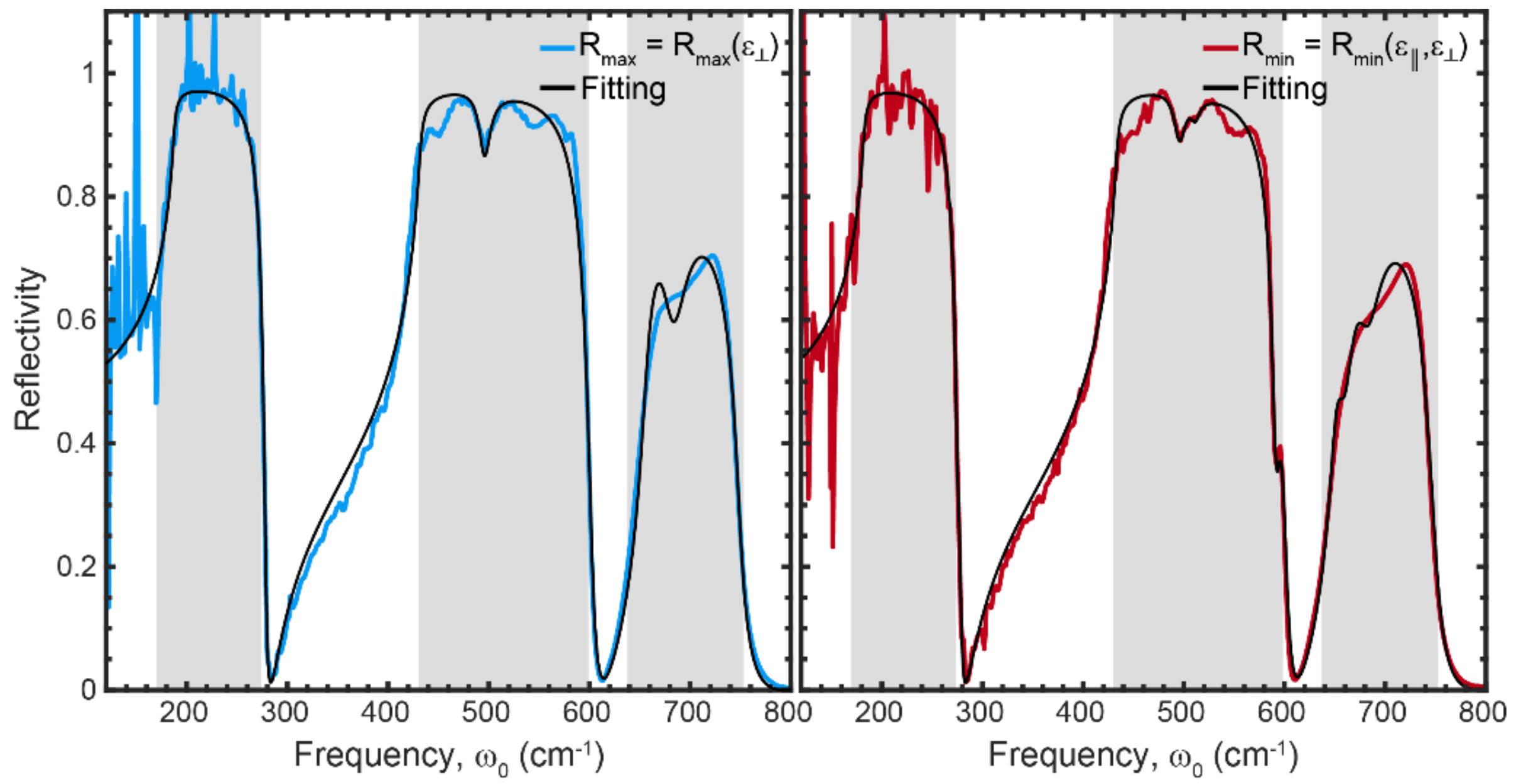

Figure S7. Fitting the reflectivity spectra. The left panel represents R_{max}=$R_\perp(\varepsilon_\perp)$, blue line, and the fitted curve, black line, supposing normal incidence of light. Right panel represents R_{min}=$R_{pr}(\varepsilon_\parallel, \varepsilon_\perp)$, red curve and the fitted curve, black line, employing Equation 14. R_{max} has been fitted employing a simple Drude-Lorentz oscillators model, with 5 phonon modes. R_{min} has been fitted with input from $\varepsilon_\perp$ fitted in R_{max}, employing in addition the 5 phonon modes of $\varepsilon_\parallel$.

Component	ε_∞	ω_{TO} (cm^{-1})	ω_p (cm^{-1})	γ (cm^{-1})
$\varepsilon_\perp$	5.07	187.58	779.49	4.0124
		431.76	972.87	5.9136
		496.79	134.33	13.602
		655.28	371.15	14.718
		684.9	160.93	29.64
$\varepsilon_\parallel$	4.42	165.1	737.78	5.6217
		438.27	857.88	3.4048
		512.35	94.214	10.956
		639.91	286.09	8.8241
		672.47	277.66	26.178

Table S1. Fitted Drude-Lorentz parameters for $\varepsilon_\parallel$ and $\varepsilon_\perp$.

Figure S8 plots the real part of the modelled permittivity in the three RBs employing the parameters of Table S1, with the red curve representing the real part of the $\varepsilon_\parallel$ permittivity and the blue curve the $\varepsilon_\perp$ component.

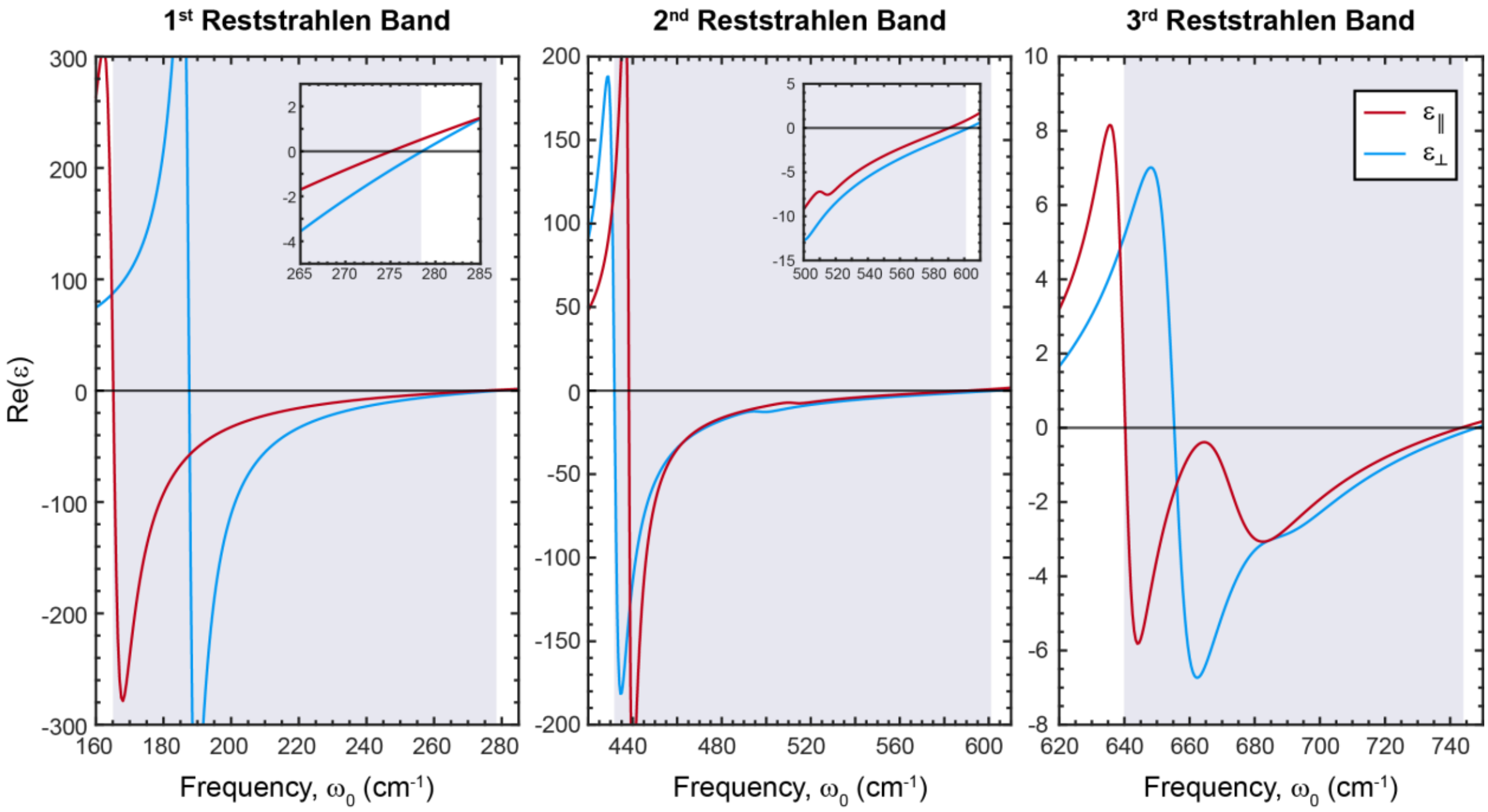

Figure S8. Infrared permittivity of LAO. For the sake of clarity, the image has been divided into three panels with different scales. Red curves represent $\varepsilon_{\parallel}$ while blue curves represent $\varepsilon_{\perp}$. Grey shaded regions represent the RBs. Inset panels in the 1st and 2nd RBs represent a zoom-in of $\mathrm{Re}(\varepsilon)$ at frequencies close to ω_{LO}, stressing the hyperbolic regimes in the material.

Supplementary Section S3: Formation of ferroelastic domains in LAO

A LAO(001) crystal can possess four different domains, each consisting of a pseudocubic unit-cell, with a compression axis along the [111] or its symmetrically equivalently directions, see Fig. S9 a and Fig. 1b). The unit cell of any given domain shares identical boundary faces with two other domains. Specifically, domain I (III) shares one matching face with domain II (IV) (Fig. S9 b) and its unit-cell cut diagonally fits perfectly to the diagonal cut of domain III (II) (Fig. S9 c); this pairwise structural compatibility is replicated across all four domains. This allows for the formation of four strain-free interfaces, so called twin walls (Fig S9 d) and the domains tesselate in a characteristic chevron pattern, seen in Fig S9 e, f.

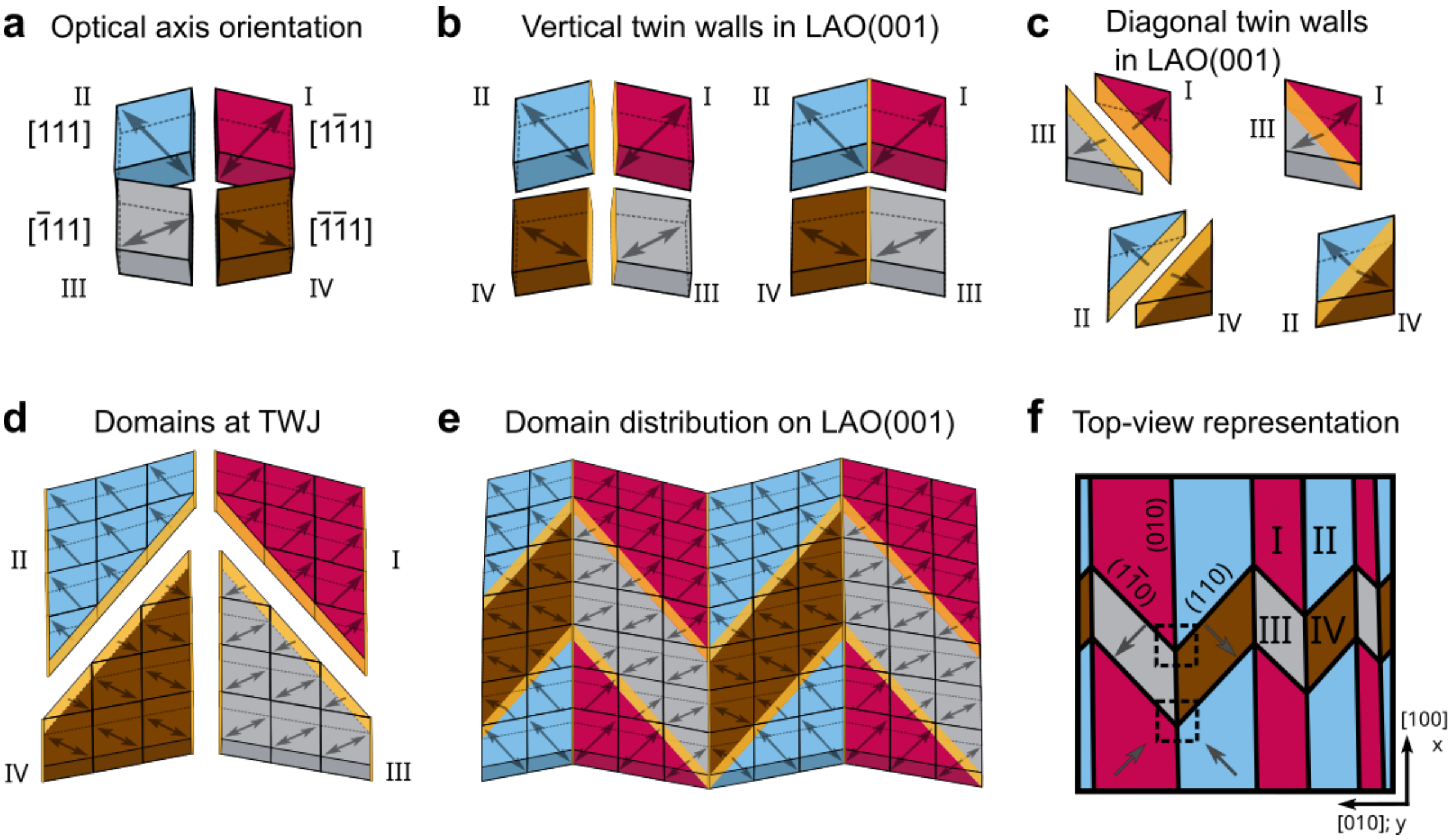

Figure S9: Schematic representation of the relation between pseudocubic unit-cells and domain tessellation. **a** Pseudocubic unit-cells and their optical axis orientation of the four possible domains. Each unit-cell is a cube distorted along their optical axes, which is aligned with the [111] axis for domain II (blue cube), and rotated by 90° three times for domains III, IV, and I. b), c) Highlighted faces of the unit-cells, which form the strain free **b** vertical TW between domains I and II as well as domains III and IV, and **c** 45° TWs between domains I and III, as well as domains II and IV. **d, e** Schematic representation of the four domains consisting of their unit-cells meeting at a TWJ (d), and tessellating the (001) plane (e), with **f** the top-view representation used in the main text Fig. 1c).

To further clarify the relation between TWs, domains and their optical axis, Fig. S10 a) shows a schematic representation of a LAO bulk crystal with all TWs and Fig10b) the orientation of the optical axis for each type of TW.

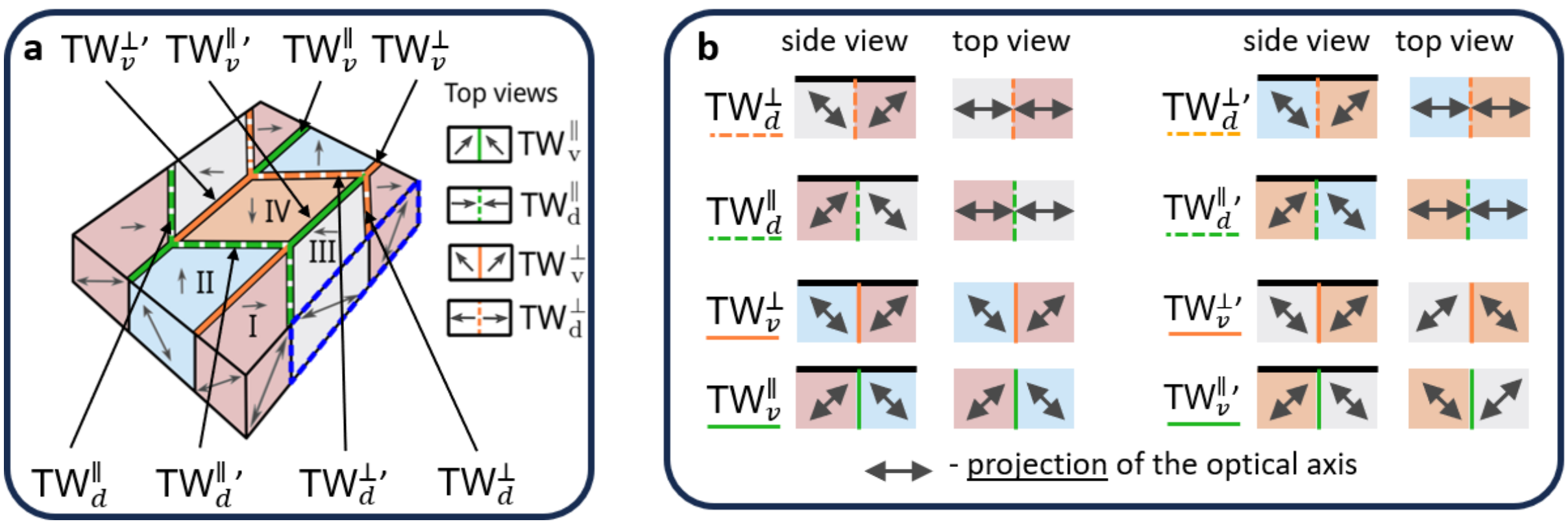

Figure S10: Orientation of optical axes for all possible TWs. Schematic representation of **a** a LAO bulk crystal with the four permitted domains, separated by TWs, forming the characteristic chevron pattern, and **b** top and side view of all possible TWs, with grey arrows indicating the projection of the optical axes.

Supplementary Section S4: Twin wall junctions in LAO and their near-field signal in all Reststrahlen bands measured by FEL-SNOM

In the main text the twin wall (TW) notation was introduced with subscripts v/d to differentiate between vertical and diagonal TWs, and a superscript of ∥ / ⊥ to indicate the frequency-dependent optical behaviour of each set of TWs. Since the designations *vertical* and *diagonal* are contingent on sample orientation and carry no crystallographic meaning, we propose a Miller index-based notation in which TW_v and TW_d are replaced by TW_{hkl}, where hkl is trivially the plane in which the TW exists, such as (010). Note that given that the TWs along $(1\overline{1}0)$ and (110) within one set of 45° walls are all equivalent in behaviour, for ease these TWs will be described as a set of planes $TW_{\{110\}}$, rather than with their individual Miller indices. The superscript ∥ $(\perp)$ will be the same as in the main text, leading to a total of 4 possible TWs with 2 distinguishable cases: i) $TW^{\parallel}_{(010)}$ and $TW^{\parallel}_{\{110\}}$, (or together simply just $TW^{\parallel}_{(010),\ \{110\}}$=$TW^{\parallel}$) where the optical axes point toward the TW surface junction, in a head-to-head configuration; and ii) $TW^{\perp}_{(010)}$ and $TW^{\perp}_{\{110\}}$, (and similarly together denoted as $TW^{\perp}_{(010),\ \{110\}}$=$TW^{\perp}$) forming a tail-to-tail configuration. With always four types of TWs meeting at any twin wall junction (TWJ), Tab. S2 presents the composition of the two possible TWJ:

	$TW^{\parallel}_{(010)}$	$TW^{\parallel}_{\{110\}}$	$TW^{\perp}_{(010)}$	$TW^{\perp}_{\{110\}}$
TWJ_1	1x	-	1x	2x
TWJ_2	1x	2x	1x	-

Table S2. Composition of the two possible TWJs on a LAO(001) surface. Each TWJ consists of in total 4 TWs, 2 oriented along the (010)-plane and 2 along the set of {110}-planes. The number shows whether and how often a TW is present in the respective TWJ.

As shown in Fig. S8, there are 3 Reststrahlen bands of LAO in the THz regime and FEL-s-SNOM measurements were carried out in all three of them, even though only the results of RB_1 are shown in the main text, due to the exceptional contrast displayed. The range in which one can visibly differentiate between the bulk material and the TWs in the first RB is from 7.79 THz to 8.16 THz, in the second RB between 16.19 THz and 17.69 THz and in the third RB from 18.59 THz to 20.98 THz. In Fig. S11 measurements of TWJ_1 at two specific frequencies are presented for each RB, showing a clear inversion of contrast for RB_1 and RB_2. At RB_3, the contrast is not as high, due to the intrinsic losses of the material in this band, as also found in the SINS data (see S5 and Fig. S13). Notably, qualitatively the same contrast is reproduced within all RBs, demonstrating the resonant character of the TW signal.

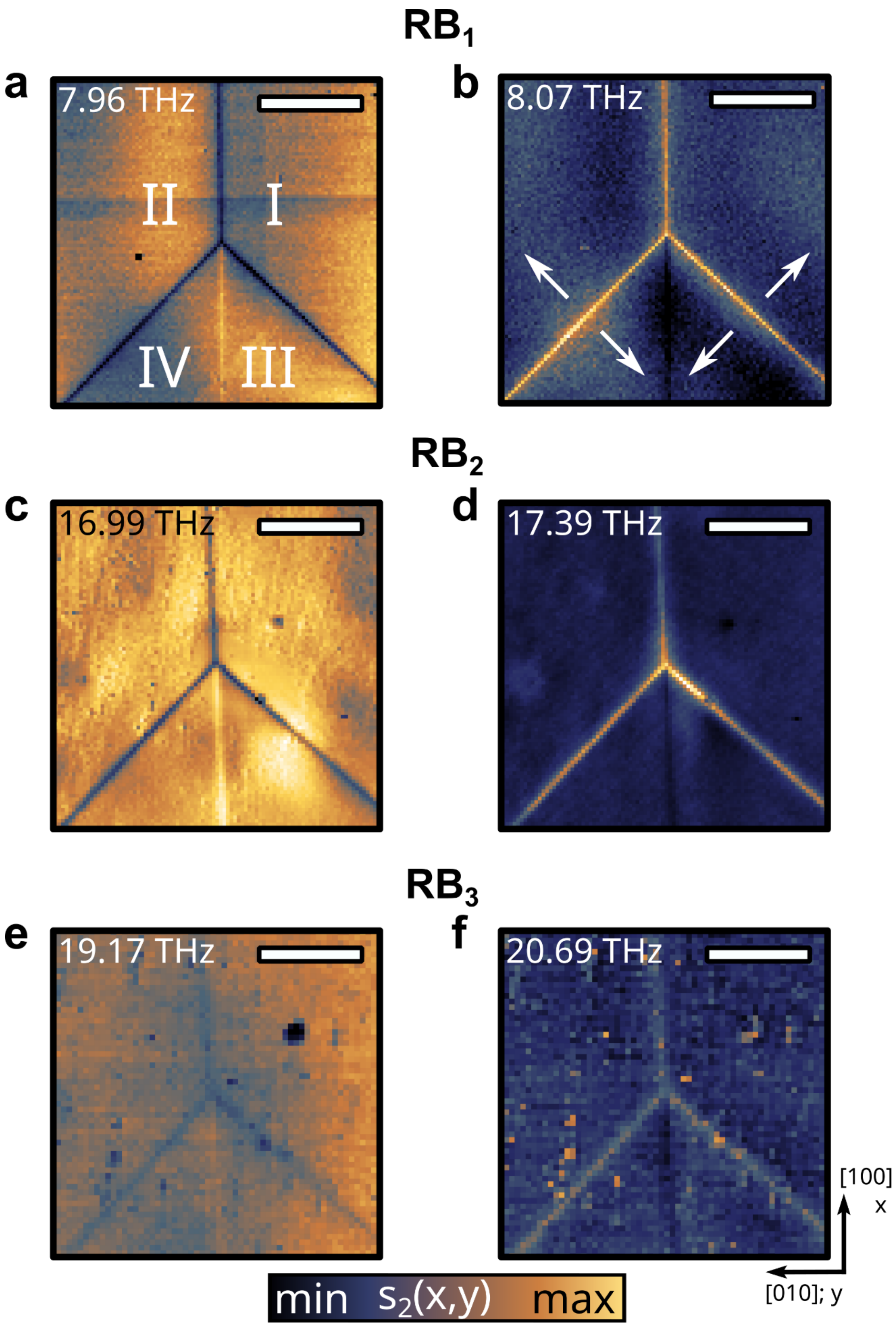

Figure S11. Spectral response in all 3 RBs at selected frequencies measured by FEL-SNOM on junction TWJ_1. Second-harmonic near-field signal $s_2(x,y)$ **a, b** in the first RB at 7.96 THz and 8.07 THz, **c, d** in the second RB at 16.99 THz and 17.39 THz, and **e, f** s2(x,y) in the third RB at 19.17 THz and 20.69 THz. All scale bars are 10 μm.

At 8.07 THz a high-resolution scan was performed in order to investigate the width of the TWs. The result of that can be seen in Fig. S12. With an excitation wavelength of 37.15 μm (8.07 THz) and a FWHM of 143 nm of $TW_{\{110\}}^{\perp}$, a lateral confinement of 260 is achieved. Note that the experimental confinement is a convolution of the TW width and the SNOM-tip radius. FEM simulations predict a confinement ration of over 1000 (see main text).

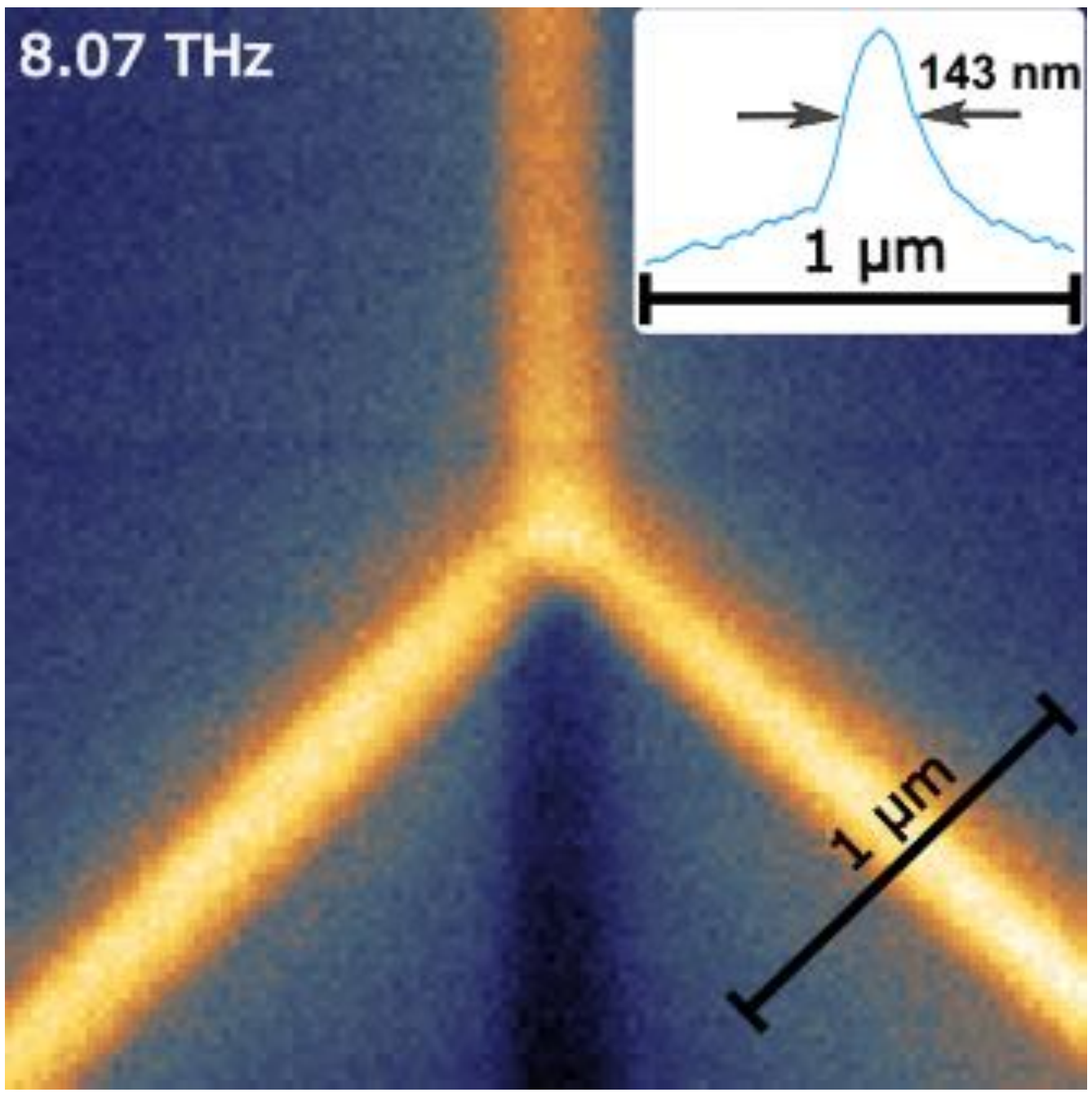

Figure S12. High-resolution optical image of TWJ_1 at 8.07 THz with the inset showing a line profile of a 1 μm length over $TW_{\{110\}}^{\perp}$ with a FWHM of 143nm, demonstrating a lateral confinement of 260.

Supplementary Section S5: LAO phonon-polariton excitation demonstrated by synchrotron infrared nano-spectroscopy (SINS)

Synchrotron infrared nano-spectroscopy (SINS) was performed in a (001)-grown LAO sample to demonstrate polaritonic launching and excitation. SINS is a spectroscopic technique which is based on a scattering-type scanning near-field optical microscope (s-SNOM), which allows nanoscale resolved absorption spectra of the samples to be obtained with wavelength-independent spatial resolution. Figure S13A shows an optical microscope image of the LAO sample employed for demonstrating the polaritonic launching via a gold bar fabricated through optical lithography. The picture has been taken with an optical microscope employing 2 crossed polarizers for the sake of visualization of the LAO twin domains, implying that light reflected from the Au bar is blocked, and thus its black colour. Figure S13B shows a SINS point spectrum (third harmonic of the tip oscillation frequency, normalised to Au, indicated by the yellow dot in Figure S13A) taken at the centre of a twin domain, far from any twin boundary. The spectrum shows an enhancement of the near-field optical signal at frequencies within the three RBs of LAO, indicating polaritonic excitation within these frequency bands. To demonstrate the polaritonic launching and measure the dispersion, we have performed SINS linescans at a direction perpendicular to the gold bar (along yellow dashed line in figure S13A, scaled to the total length of the SINS linescan). Figure S13C shows the third harmonic SINS linescan spectra normalised

to the Au reference, $s_3/s_{3,Au}$. We can clearly see maxima of the near-field signal at frequency regions coinciding with the LAO's RBs, in line with the spectrum shown in Figure S13B. Furthermore, we observe signal oscillations within the first and second RBs (the signal is too low in the 3rd RB of LAO as to observe any oscillation, due to the intrinsic losses of the material at these frequencies), with an oscillation period that decreases as the light's frequency increases. Figure S13D plots some of the SINS spectra at frequencies that clearly show the fringe oscillations. We have artificially shifted them along the vertical direction for the sake of clarity. We can assign these oscillations with the propagating surface phonon polaritons excited by the Au bar [4]. By fitting the spectral profiles to an exponentially decaying sinusoidal function, we can extract the polaritonic wavelength:

$$y=y_0+A \cdot \exp(-x/L_p) \cdot sin(2\pi x/\rho+\phi_0), \qquad (16)$$

with y_0 an offset to the near-field signal, A the amplitude of the polaritonic wave, L_p the propagation length, ρ the fringe spacing and ϕ_0 a phase offset. It should be noted that, as we are working with bulk LAO, polaritons are not highly confined, and so the periodicity of the measured near-field oscillations (the fringe spacing, ρ) does not correspond to the polaritonic wavelength, λ_p [4]. By geometrical considerations, it can be calculated that the fringe spacing ρ can be related to the polaritonic wavelength, λ_p, by means of the following equation [5, 6]:

$$\lambda_p=\frac{\rho}{1+\frac{\rho}{\lambda_0}cos\alpha}, \qquad (17)$$

with λ_0 the wavelength of the incident light, $\lambda_0=1/\omega_0$, and α the angle of the incident light, $\alpha \approx 45°$. It should be noted that, due to the transition from one twin domain to the adjacent one during the SINS linescan (see yellow dashed line in Figure S13a) polaritons are weakly reflected back at the domain wall. This is not an issue for extracting λ_p due to the very small difference between the permittivity components $\varepsilon_\perp$ and $\varepsilon_\parallel$, differing basically in the optical axis orientation. However, it clearly affects the damping term (the propagation length L_p), extremely sensitive to inhomogeneities at the surface of the polaritonic material. This very weak back-reflection also explains why only a weak polaritonic interference fringes are observed near the TWs in the s-SNOM scans shown in Fig. 2 of the main text: the SPhPs are predominantly transmitted across the ferroelastic domain boundaries, with only a small reflected component insufficient to generate a detectable standing-wave pattern. Therefore, no reliable experimental information about the polaritonic propagation length or lifetime, τ, can be extracted.

Employing the permittivity calculated in Supplementary Section S2, we have calculated the imaginary part of the Fresnel reflection coefficient as a function of momentum and frequency, $r_{pp}(q,\omega)$, through a transfer matrix method as the polaritonic dispersion is contained within the denominator of $r_{pp}(q,\omega)$ [7]. The calculation is shown in Figure S13e as a false colour plot, confirming a polaritonic branch in every RB. The experimental dispersion, calculated by fitting the SINS profiles with Equations 16 and 17, with the experimental polaritonic momentum given by $q_p=2\pi/\lambda_p$, is represented by the blue dots, obtaining excellent agreement between measurements and calculations.

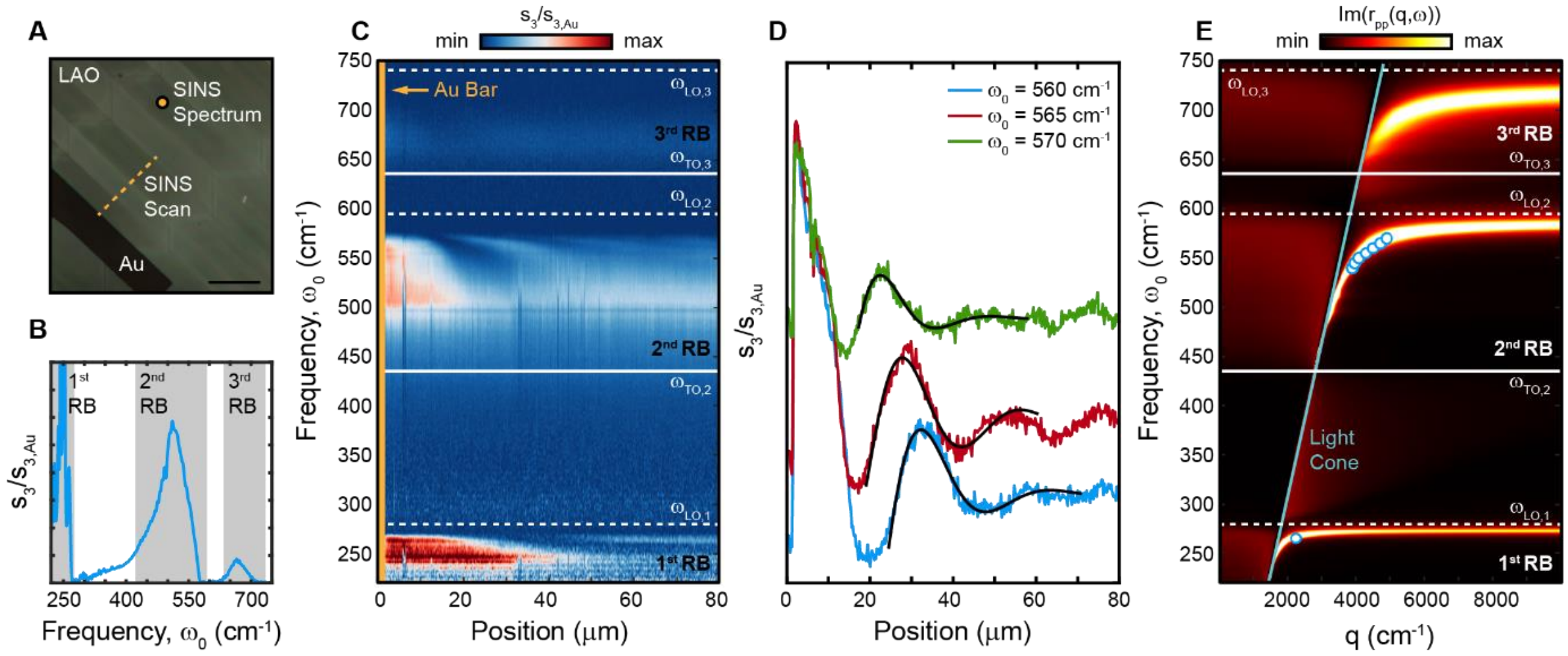

Figure S13. Polaritonic launching in LAO through a gold bar. **A** Optical image of a [100] LAO substrate presenting its typical chevron pattern. An Au rod was fabricated as polaritonic launcher by means of optical lithography. 2 crossed polarizers were employed for taking the picture, as it enhances the optical contrast between the different LAO domains. Scale bar is 50 microns. **B** LAO SINS spectrum (third harmonic, normalised to gold) taken at the yellow point position of **A**. We clearly observe an enhancement of the near-field signal at the frequency regions within the RBs. **C** LAO SINS linescan taken along the yellow dashed line in **A**. Polaritonic oscillations can be clearly observed within the first and second RBs. The Au bar position is marked with a yellow stripe at the very left of the image. **D** Near field $s_3/s_{3,Au}$ profiles extracted from **C** at selected frequencies (560, 565, and 570 cm^{-1} in blue, red, and green, respectively) showing the polaritonic oscillations. The profiles were fitted employing Equation 16 for extracting fringe periodicity. Black lines represent the fittings for every profile. **E** LAO polaritonic dispersion (false colour plot) calculated through the transfer matrix method. We can observe a polaritonic branch within every RB. The dispersion of light is depicted as a light blue solid line. Blue circumferences depict the polaritonic dispersion measured by extracting the polaritonic wavelength λ_p from the profiles in **D** employing Equations 16 and 17.

Supplementary Section S6: Analytical Point-Dipole Model for Twin-Domain Walls

In order to analytically describe the fundamental interaction of a s-SNOM probe with the LAO twin-domain walls (TW), we modify here the analytical point-dipole model as originally introduced by Knoll and Keilmann [8] and thereby extend it for interaction with an anisotropic sample that includes a domain wall, i.e. a boundary between sample areas with different permittivity tensors. Our model assumes an infinitely extended sample surface located at *z = 0*, with the probe in air above (z > 0), and the sample below (*z = 0*). The TW, located at *x = 0* separates the sample half-space into two quarter spaces (see Fig. S14A). The two adjacent sample domains are then represented by two laterally separated polarizable spheres, here placed at *z* = −*h* and *x* = ±*h,* with in general arbitrarily oriented anisotropic permittivity tensors. Similar to the conventional dipole-model, the tip is assumed to be represented by an isotropic sphere and is placed at a height *z* = *h*, directly above the TW-surface junction (*x* = *0*), i.e. symmetrically between the two sample spheres (see Fig. S14A).

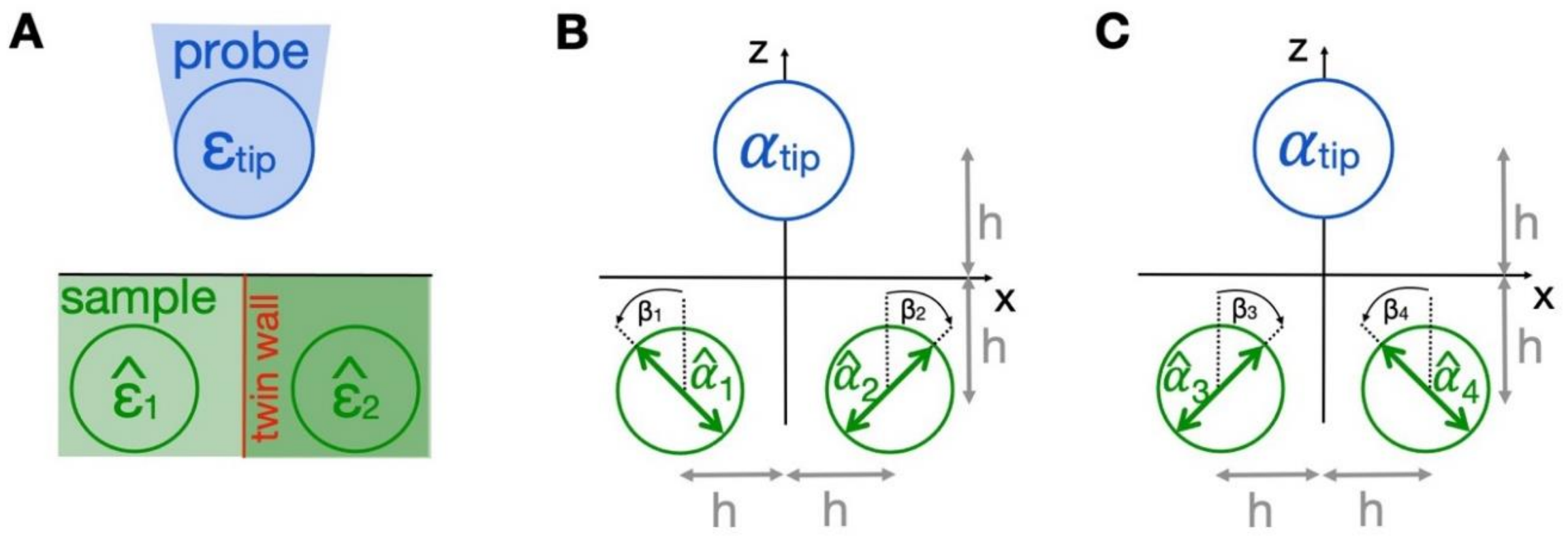

Figure S14. Model system representing a near-field probe next to a twin-domain wall (TW) separating two sample areas with different permittivity tensors. **A** General concept with probe and sample areas being represented by polarizable spheres of permittivites ε_{tip} (isotropic tip) and $\widehat{\varepsilon_1}$, $\widehat{\varepsilon_2}$ (anisotropic sample areas), respectively. **B,C** The two cases A, B with different orientations of the optical axis (green arrows) and corresponding polarizability tensors α_i at the TWs as discussed in detail in this supplementary (see description in text).

It shall be noted, that this model system acts as a very first approximation only, illustrating the coupling mechanism and the relevant system parameters. Even though it qualitatively reproduces the experimentally and numerically observed response surprisingly well (see Fig. 3 in main text), it is expected to be strongly limited in terms of giving quantitative numbers for e.g. distance dependence of the signal, domain/domain-wall contrast, and exact spectral position of the resonances. Moreover, it is suited only for tip positions directly above the sample junction as well as for planar domain walls orthogonal to the sample surface. For a more detailed theoretical analysis either numerical simulations (as carried out in this work) or a representation of the sample by a manifold of point dipoles (as applied e.g. in [9]) are needed.

Besides these limitations, the advantages of the here discussed simple extension of the dipole model for qualitative description of the LAO-TW signature include

- the possibility to introduce two sample areas with different optical response,
- direct implementability of arbitrary sample anisotropy (including in particular optical axis non-orthogonal to the sample surface, unlike e.g. in [10, 11]), and
- application to arbitrary excitation polarisation, which are not possible when e.g. using the finite dipole model [12].

The derived model results in a simple, analytical solution of the scattering cross sections in s-SNOM for different anisotropic domain configurations. Assuming twin-domain walls with mirrored symmetry, the model can be solved fully analytically resulting in formulas with distinct similarities compared to the conventional point-dipole model as shown below. In the following, without restriction, we exemplarily show the derivation of these formulas for excitation fields strictly orthogonal to the surface as well as for a birefringent material with optical axis orientation within the incident plane of the scattering process and under 45° with respect to the sample surface in two different possible cases (see Figs. S14B,C). However, for the results in the main

manuscript, the same modelling approach has been followed semi-analytically via a python script for all possible LAO twin-domain walls including canted optical axis orientation.

All **three polarizable spheres** (tip, left and right sample domain) in this model are assumed to be small compared to the wavelength, resulting in induced point-dipoles, which may be represented by their corresponding polarizability tensors. Assuming a metal-coated tip in the mid-IR spectral regime surrounded by air (radius a, $\varepsilon_{tip} \ll -1000$ and ε_m=1), the tip polarizability is given by [13]:

$$\alpha_{\text{tip}} = 4\pi a^3 \underbrace{\frac{\varepsilon_{\text{tip}} - \varepsilon_m}{\varepsilon_{\text{tip}} + 2\varepsilon_m}}_{\approx 1} \approx 4\pi a^3. \tag{18}$$

The two sample dipoles on the other hand are represented by uniaxial polarizability tensors $\widehat{\alpha}_{LAO}$:

$$\widehat{\alpha}_{LAO} = 4\pi b^3 \begin{pmatrix} \eta_\perp & 0 & 0 \\ 0 & \eta_\perp & 0 \\ 0 & 0 & \eta_\parallel \end{pmatrix}, \tag{19}$$

with *b* the sphere radius and

$$\eta_\perp = \frac{\varepsilon_\perp - 1}{\varepsilon_\perp + 2}, \quad \eta_\parallel = \frac{\varepsilon_\parallel - 1}{\varepsilon_\parallel + 2}, \tag{20}$$

including the permittivity component orthogonal ($\varepsilon_\perp$) and parallel ($\varepsilon_\parallel$) to LAO's optical axis, respectively.

For representation of the twin-domain wall in case A (compare Fig. S14B), the polarizabilities on the two sides of the junction are tilted symmetrically by the angles β_1=β and β_2=-β (in the calculated example assuming β=45°) yielding

$$\widehat{\alpha_1} = \hat{R}(\beta_1) \cdot \widehat{\alpha}_{LAO} \cdot \hat{R}^T(\beta_1) = 4\pi b^3 \begin{pmatrix} \frac{1}{2} \cdot (\eta_\perp + \eta_\parallel) & 0 & \frac{1}{2} \cdot (\eta_\perp - \eta_\parallel) \\ 0 & \eta_\perp & 0 \\ \frac{1}{2} \cdot (\eta_\perp - \eta_\parallel) & 0 & \frac{1}{2} \cdot (\eta_\perp + \eta_\parallel) \end{pmatrix}, \tag{21}$$

$$\widehat{\alpha_2} = \hat{R}(\beta_2) \cdot \widehat{\alpha}_{LAO} \cdot \hat{R}^T(\beta_2) = 4\pi b^3 \begin{pmatrix} \frac{1}{2} \cdot (\eta_\perp + \eta_\parallel) & 0 & \frac{1}{2} \cdot (-\eta_\perp + \eta_\parallel) \\ 0 & \eta_\perp & 0 \\ \frac{1}{2} \cdot (-\eta_\perp + \eta_\parallel) & 0 & \frac{1}{2} \cdot (\eta_\perp + \eta_\parallel) \end{pmatrix}, \tag{22}$$

with $\hat{R}(\beta)$ being the rotation matrix

$$\hat{R}(\beta) = \begin{pmatrix} \cos(\beta) & 0 & -\sin(\beta) \\ 0 & 1 & 0 \\ +\sin(\beta) & 0 & \cos(\beta) \end{pmatrix} \tag{23}$$

and $\hat{R}^T(\beta)$ its transposed matrix.

Following the derivation in [8], the **near-field coupling** is implemented by the following steps:

(1) the external field of the incident light induces an initial point dipole in the tip,

(2) the field of the tip dipole at the position of the sample excites virtual sample dipoles

(3) the fields of these virtual sample dipoles modify the field at the position of the tip. Note that these virtual dipoles are assumed not to interact with each other, but solely modify the field at the position of the tip dipole,

(4) the tip dipole itself is modified by the sample-dipole fields resulting in a retro-action that represents the near-field coupling of tip and sample, and

(5) the near-field coupled system is described by a total polarizability tensor $\hat{\alpha}_{tot}$ that includes both modified tip polarizability and sample polarizabilities.

Here, we discuss two different cases of TW, namely cases A and B, as illustrated in Figs. S14B,C. These configurations roughly resemble the 45°-domain walls for (001)-cut sample surfaces which are corresponding to the (110) and $(1\bar{1}0)$ planes of the crystal. However, more complex walls and varying angles/sample dipole positions may be addressed with the same model concept as e.g. done via a python script for simulating the responses of all possible LAO domain walls as shown in Fig. 3 of the main manuscript.

These steps towards near-field coupling for the two cases A and B here yield the following:

(1) The initially induced tip dipole is given by

$$\vec{P}_{\text{tip}} = \varepsilon_0 \cdot \alpha_{\text{tip}} \cdot \vec{E}_0 \approx \varepsilon_0 \cdot 4\pi a^3 \cdot E_0 \begin{pmatrix} 0 \\ 0 \\ 1 \end{pmatrix} \quad \text{at} \quad \vec{r}_0 = \begin{pmatrix} 0 \\ 0 \\ h \end{pmatrix} \tag{24}$$

for an external excitation field $\vec{E}_0$ orthogonal to the sample surface (*z*-direction), and a tip-sample separation of *h=0,785 a* used in the python code following values from literature [11, 14].

(2) Its field at the positions of the two polarizable sample spheres may be calculated using

$$\vec{E}(\vec{P}, \vec{r}) = \frac{1}{4\pi\varepsilon_0} \frac{3\vec{n}\cdot(\vec{P}\cdot\vec{n}) - \vec{P}}{|\vec{r} - \vec{r}'|^3} \tag{25}$$

resulting in

$$\vec{E}_{1,\text{tip}} = \vec{E}(\vec{P}_{\text{tip}}, \vec{r}_1) = \frac{a^3}{5^{5/2} h^3} E_0 \begin{pmatrix} 6 \\ 0 \\ 7 \end{pmatrix} \quad \text{at} \quad \vec{r}_1 = \begin{pmatrix} -h \\ 0 \\ -h \end{pmatrix}, \tag{26}$$

$$\vec{E}_{2,\text{tip}} = \vec{E}(\vec{P}_{\text{tip}}, \vec{r}_2) = \frac{a^3}{5^{5/2} h^3} E_0 \begin{pmatrix} -6 \\ 0 \\ 7 \end{pmatrix} \quad \text{at} \quad \vec{r}_2 = \begin{pmatrix} h \\ 0 \\ -h \end{pmatrix}. \tag{27}$$

For the two different cases, we assume the following sample configurations (compare Fig. S14B,C):

$$\underline{\text{Case A:}} \quad \text{Sphere 1:} \quad \vec{r}_1 = \begin{pmatrix} -h \\ 0 \\ -h \end{pmatrix}, \quad \hat{\alpha}_1 \tag{28}$$

$$\text{Sphere 2:} \quad \vec{r}_2 = \begin{pmatrix} +h \\ 0 \\ -h \end{pmatrix}, \quad \hat{\alpha}_2$$

$$\underline{\text{Case B:}} \quad \text{Sphere 3:} \quad \vec{r}_3 = \vec{r}_1, \quad \hat{\alpha}_3 = \hat{\alpha}_2 \tag{29}$$

$$\text{Sphere 4:} \quad \vec{r}_4 = \vec{r}_2, \quad \hat{\alpha}_4 = \hat{\alpha}_1$$

The induced virtual dipoles in the anisotropic spheres 1 to 4 are calculated via the formula

$$\vec{P}_i = \varepsilon_0 \cdot \hat{\alpha}_i \cdot \vec{E}\left(\vec{P}_{\text{tip}}, \vec{r}_i\right) \quad \text{with} \quad i = 1, 2, 3, 4 \tag{30}$$

with $\vec{E}\left(\vec{P}_{tip}, \vec{r}_i\right)$ taken from the above equations for the tip-dipole field at the positions of the sample spheres resulting in the following sample-dipole combinations for the two cases

$$\underline{\text{Case A:}} \quad \text{Sphere 1:} \quad \vec{P}_1 = \varepsilon_0 \cdot 4\pi a^3 \cdot \frac{b^3}{2 \cdot 5^{5/2} \cdot h^3} E_0 \begin{pmatrix} 13\eta_\perp - \eta_\parallel \\ 0 \\ 13\eta_\perp + \eta_\parallel \end{pmatrix} \quad \text{at} \quad \vec{r}_1 \tag{31}$$

$$\text{Sphere 2:} \quad \vec{P}_2 = \varepsilon_0 \cdot 4\pi a^3 \cdot \frac{b^3}{2 \cdot 5^{5/2} \cdot h^3} E_0 \begin{pmatrix} -13\eta_\perp + \eta_\parallel \\ 0 \\ 13\eta_\perp + \eta_\parallel \end{pmatrix} \quad \text{at} \quad \vec{r}_2$$

$$\underline{\text{Case B:}} \quad \text{Sphere 3:} \quad \vec{P}_3 = \varepsilon_0 \cdot 4\pi a^3 \cdot \frac{b^3}{2 \cdot 5^{5/2} \cdot h^3} E_0 \begin{pmatrix} -\eta_\perp + 13\eta_\parallel \\ 0 \\ \eta_\perp + 13\eta_\parallel \end{pmatrix} \quad \text{at} \quad \vec{r}_3 \tag{32}$$

$$\text{Sphere 4:} \quad \vec{P}_4 = \varepsilon_0 \cdot 4\pi a^3 \cdot \frac{b^3}{2 \cdot 5^{5/2} \cdot h^3} E_0 \begin{pmatrix} \eta_\perp - 13\eta_\parallel \\ 0 \\ \eta_\perp + 13\eta_\parallel \end{pmatrix} \quad \text{at} \quad \vec{r}_4$$

(3) The superposition of the fields of these dipoles at the position of the tip is then given by

$$\underline{\text{Case A:}} \quad \vec{E}_A = \vec{E}\left(\vec{P}_1, \vec{r}_0\right) + \vec{E}\left(\vec{P}_2, \vec{r}_0\right)$$

$$= \underbrace{\frac{4b^3}{5^5 h^3} \cdot \left(169\eta_\perp + \eta_\parallel\right)}_{=\beta''_A} \cdot \frac{1}{16\pi h^3} \cdot \underbrace{\varepsilon_0 \cdot 4\pi a^3 \cdot E_0 \begin{pmatrix} 0 \\ 0 \\ 1 \end{pmatrix}}_{=\vec{P}_{\text{tip}}} = \beta''_A \cdot \frac{\vec{P}_{\text{tip}}}{16\pi h^3} \tag{33}$$

$$\underline{\text{Case B:}} \quad \vec{E}_B = \vec{E}\left(\vec{P}_3, \vec{r}_0\right) + \vec{E}\left(\vec{P}_4, \vec{r}_0\right)$$

$$= \underbrace{\frac{4b^3}{5^5 h^3} \cdot \left(\eta_\perp + 169\eta_\parallel\right)}_{=\beta''_B} \cdot \frac{1}{16\pi h^3} \cdot \underbrace{\varepsilon_0 \cdot 4\pi a^3 \cdot E_0 \begin{pmatrix} 0 \\ 0 \\ 1 \end{pmatrix}}_{=\vec{P}_{\text{tip}}} = \beta''_B \cdot \frac{\vec{P}_{\text{tip}}}{16\pi h^3} \tag{34}$$

Note that due to symmetry at the twin-domain wall (with the tip dipole being placed laterally exactly at the position of the wall at *x*=*y*=*0*), the sum of the two sample dipole fields at the position of the tip is orthogonal to the surface and with that parallel to the initial tip dipole. Moreover, the factors β''_A and β''_B are utilized later to calculate the total polarizability.

(4) The induced tip dipole is modified by the fields $\vec{E}_{A/B}$ of the sample dipoles resulting in the near-field coupling via

$$\vec{P}_{\text{tip}} = \varepsilon_0 \alpha_{\text{tip}} \cdot \left(\vec{E}_0 + \vec{E}_{A/B}\right)$$

$$= \varepsilon_0 \alpha_{\text{tip}} \cdot \left(\vec{E}_0 + \beta''_{A/B} \cdot \frac{\vec{P}_{\text{tip}}}{16\pi h^3}\right) \tag{35}$$

Solving this equation for $\vec{P}_{tip}$ yields

$$\vec{P}_{\text{tip}} = \varepsilon_0 \cdot \frac{\alpha_{\text{tip}}}{1 - \frac{\alpha_{\text{tip}} \beta''_{A/B}}{16\pi h^3}} \cdot \vec{E}_0 \tag{36}$$

(5) The total dipole of the near-field-coupled tip-sample system is represented by the sum of all dipoles, which are assumed to be located at the same position when observed from the far-field ($r \gg h$). The sample dipoles for the two cases A and B therefore can be summarized by

$$\underline{\text{Case A:}} \quad \vec{P}_A = \vec{P}_1 + \vec{P}_2 = \underbrace{\frac{b^3}{5^{5/2} \cdot h^3} \cdot (13\eta_\perp + \eta_\parallel)}_{=\beta'_A} \cdot \underbrace{\varepsilon_0 \cdot 4\pi a^3 \cdot E_0 \begin{pmatrix} 0 \\ 0 \\ 1 \end{pmatrix}}_{=\vec{P}_{\text{tip}}} = \beta'_A \cdot \vec{P}_{\text{tip}} \quad (37)$$

$$\underline{\text{Case B:}} \quad \vec{P}_B = \vec{P}_3 + \vec{P}_4 = \underbrace{\frac{b^3}{5^{5/2} \cdot h^3} \cdot (\eta_\perp + 13\eta_\parallel)}_{=\beta'_B} \cdot \underbrace{\varepsilon_0 \cdot 4\pi a^3 \cdot E_0 \begin{pmatrix} 0 \\ 0 \\ 1 \end{pmatrix}}_{=\vec{P}_{\text{tip}}} = \beta'_B \cdot \vec{P}_{\text{tip}} \quad (38)$$

Note that due to the symmetry of the system, the total dipole in both cases is oriented orthogonal to the sample surface, i.e. in $\vec{P}_{tip}$ direction. The resulting total dipole of the near-field coupled tip-sample system then yields

$$\vec{P}_{\text{tot}} = \vec{P}_{\text{tip}} + \vec{P}_{A/B}$$
$$= \left(1 + \beta'_{A/B}\right) \cdot \vec{P}_{\text{tip}} = \varepsilon_0 \underbrace{\frac{\alpha_{\text{tip}}\left(1+\beta'_{A/B}\right)}{1 - \frac{\alpha_{\text{tip}}\beta''_{A/B}}{16\pi h^3}}}_{=\alpha_{\text{tot}}} \vec{E}_0 = \varepsilon_0 \alpha_{\text{tot}} \vec{E}_0 \quad (39)$$

In **summary**, the near-field-coupled tip-sample system, hence, is represented by its total polarizability tensor $\hat{\alpha}_{tot}$

$$\alpha_{\text{tot}} = \frac{\alpha_{\text{tip}}\left(1+\beta'_{A/B}\right)}{1 - \frac{\alpha_{\text{tip}} \cdot \beta''_{A/B}}{16\pi h^3}} \quad (40)$$

with α_{tip}=$4\pi a^3$ being the polarizability of the spherical metal tip of radius *a*, *h* being the separation between tip dipole and sample surface (typically set to *h=0.785a* [11, 14]) and the complex functions $\beta'_{A/B}$ as well as $\beta''_{A/B}$ being different for the two twin-domain wall types (Cases A and B as shown in Fig. S14B,C) and given by

Case A:

$$\beta'_A = \frac{b^3}{5^{5/2} h^3} \cdot (13\eta_\perp + \eta_\parallel)$$
$$\beta''_A = \frac{4b^3}{5^5 h^3} \cdot (169\eta_\perp + \eta_\parallel) \quad (41)$$

Case B:

$$\beta'_B = \frac{b^3}{5^{5/2} h^3} \cdot (\eta_\perp + 13\eta_\parallel)$$
$$\beta''_B = \frac{4b^3}{5^5 h^3} \cdot (\eta_\perp + 169\eta_\parallel) \quad (42)$$

Both $\beta'_{A/B}$ and $\beta''_{A/B}$ are functions of $\eta_\perp$ and $\eta_\parallel$, which depend on the complex optical sample permittivity components orthogonal ($\varepsilon_\perp$) and parallel ($\varepsilon_\parallel$) to its optical axis via

$$\eta_\perp = \frac{\varepsilon_\perp - 1}{\varepsilon_\perp + 2}, \quad \eta_\parallel = \frac{\varepsilon_\parallel - 1}{\varepsilon_\parallel + 2}. \quad (43)$$

The total polarizability tensor may be used to **calculate the different signals measured by s-SNOM** such as the complex scattered electric field $\vec{E}_{sca}$, scattering (C_{sca}) and absorption (C_{abs}) cross sections. The corresponding formulae are given by:

$$\vec{E}_{\text{sca}} \sim \alpha_{\text{tot}} \cdot \vec{E}_0$$
$$C_{\text{sca}} = \frac{k^4}{6\pi} \cdot |\alpha_{\text{tot}}|^2 \quad (44)$$
$$C_{\text{abs}} = k \cdot Im(\alpha_{\text{tot}})$$

Typically, in order to determine the resonances of a near-field coupled tip-sample system, C_{sca} is discussed as function of wavenumber. Figure S15 shows C_{sca} as function of wavenumber for the two different cases of twin domain walls in LAO. Enhanced signals in s-SNOM are typically observed around sample permittivities of $\varepsilon \approx$ -3…-1 with the exact spectral location being dependent on the tip-sample separation h. The corresponding enhancement is observed in all three Reststrahlen bands matching the experimental observation and numerical results. Due to the tilted optical axis within the domains (see Fig. S14), the two different twin-domain wall configurations (cases A and B) show resonances that are dominated either by the perpendicular or the parallel component of the dielectric tensor, depending on the axis alignment with respect to the tip-dipoles electric field at the location of the sample dipoles. This effect is illustrated in Fig. S16 by sketching the electric field lines of the tip dipole at the positions of the sample spheres.

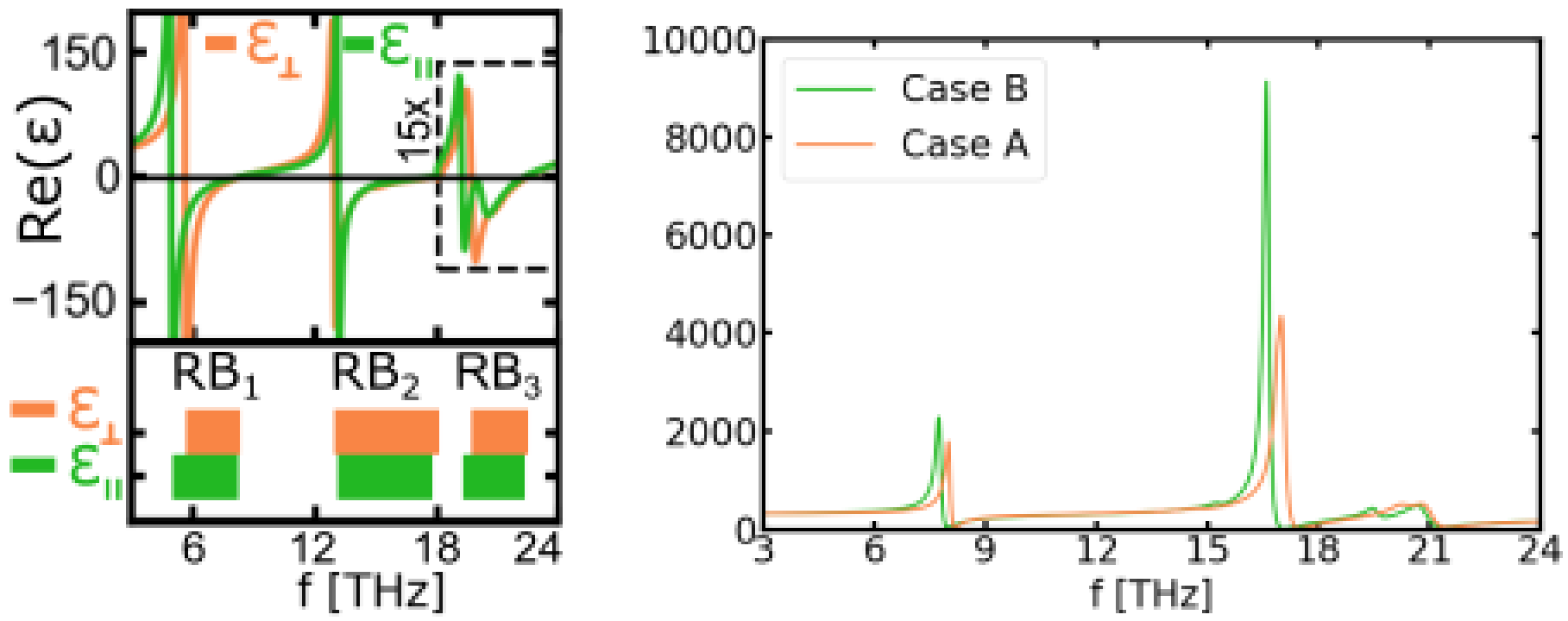

Figure S15. Permittivity data (left) and corresponding scattering cross sections (right) calculated via the here developed dipole model for twin domain walls for the two different cases as displayed in Fig. S14.

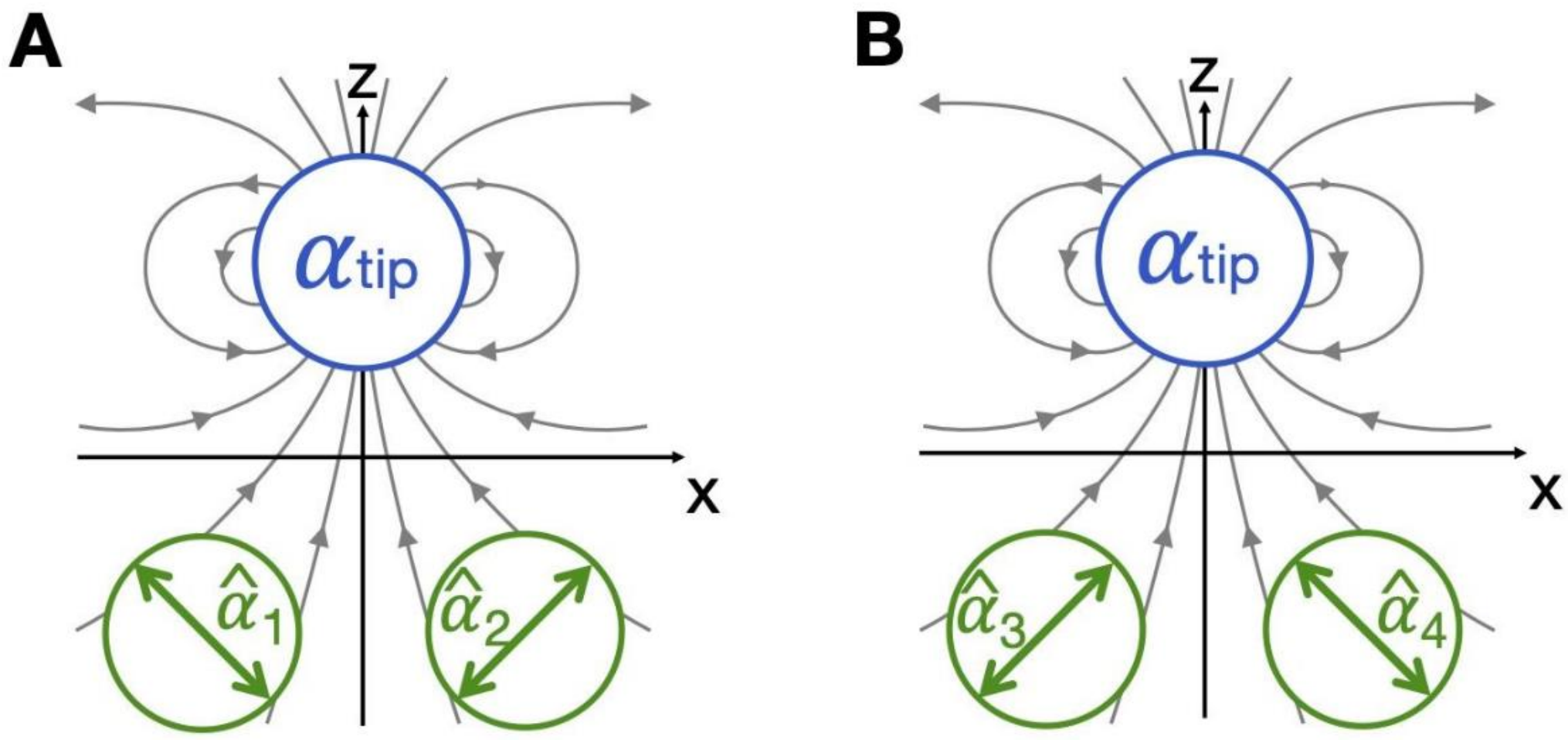

Figure S16. Illustration why the model predicts probing of the two different permittivity components of the sample for the two different cases. **A** In case A, the field lines of the tip dipole at the position of the sample dipoles are oriented mostly orthogonal to the optical axis in both domains, whereas **B** in case B, the field lines are oriented parallel to the optical axis.

In conclusion, we modified the analytical point dipole model for probing of domain walls between two sample areas with different permittivity tensors. Therefore, the sample responses of the two areas next to the tip, which is located exactly above the domain wall, are represented by anisotropic, polarizable spheres. The permittivity tensor of these spheres in principle can be chosen at will in the developed model, however here we focus on a symmetric configuration as being present for twin domain walls e.g. in (001)-cut LAO. Matching approximately the 45° twin-domain walls in (001)-cut LAO, we discuss two different cases of twin domain walls with different orientations of the optical axis and find resonances that are dominated by the permittivity component orthogonal and parallel to the optical axis for the two cases A and B, respectively. This effect is determined mathematically by the total polarizability of the near-field coupled system, which shows a resonant maximum when its denominator approaches zero.

This simple analytical model enables us to qualitatively describe the near-field signal and domain-wall contrast found experimentally on LAO within its Reststrahlen bands matching perfectly the rastering-dipole COMSOL simulations shown in Figure 2 of the Main. Whereas here, we limit ourself quite strongly in terms of polarisation of the excitation field and optical axis orientation in the domains, the model in general holds for arbitrary choices of both, which can be easily implemented e.g. in a python script which enables the calculation of the coupling without restrictions on these parameters.

Supplementary Section S7: Role of the crystal symmetry on the in-plane isofrequency contour and on the twin-wall contrast inversion.

Due to the tilting of the optical axis with respect to the (001) LAO surface, the material can potentially support ghost-polariton-like solutions [15, 16, 17]. In such modes, the wavevector can acquire simultaneous real and imaginary components even in the absence of material loss, leading to non-uniform polaritonic fields that both oscillate and decay in space. Therefore, the ghost character originates from the complex-wavevector structure imposed by the anisotropic dispersion, rather than from absorption alone. This situation should be distinguished from hyperbolic shear polaritons in low-symmetry monoclinic or triclinic crystals [18, 19, 20]. In those systems, the shear response arises from non-orthogonal phonon modes and from off-diagonal components of the dielectric tensor that cannot be fully removed by a frequency-independent rotation. As a result, the optical axes themselves disperse with frequency, producing a frequency-dependent response of the polariton propagation direction, tilted wavefronts, asymmetric energy flow, and, in some cases, topology changes between hyperbolic, canalised, and elliptical regimes. In LAO, by contrast, the material is uniaxial: the dielectric response is fully described by $\varepsilon_{\parallel}$ and $\varepsilon_{\perp}$, and the apparent in-plane anisotropy arises from rotating this tensor into the (001) laboratory frame. Consequently, ghost-polariton-like behaviour in LAO should not be interpreted as a shear-polariton response of a low-symmetry crystal. Depending on frequency and on the orientation of the tilted optical axis relative to the propagation direction, the projected in-plane dispersion may be elliptical or hyperbolic. Thus, ghost-polariton-like fields can exist in each of the RBs, whereas the classification as hyperbolic or elliptical refers to the topology of the in-plane isofrequency contour

On the other hand, the contrast inversion of the near-field signal at LAO twin walls occurs close to the transition between two polaritonic regimes. Below approximately $8.24\ \mathrm{THz}$ ($\omega_0 = 275\ \mathrm{cm}^{-1}$),

LAO exhibits an elliptical in-plane isofrequency contour, with both $\varepsilon_\perp$ and $\varepsilon_\parallel$ being negative but with different absolute value. Above this frequency, LAO enters a hyperbolic regime, where $\varepsilon_\parallel$ becomes positive while $\varepsilon_\perp$ remains negative. However, the experimentally observed twin-wall contrast inversion occurs between f_1=7.96 THz and f_2=8.07 THz, i.e. below the onset of the hyperbolic regime. Therefore, the inversion cannot be attributed to an elliptical-to-hyperbolic topological transition of the in-plane polariton dispersion. Instead, the inversion arises from the frequency-dependent local near-field response of the twin walls relative to the surrounding bulk LAO domains. Figure S17 shows the calculated in-plane isofrequency contours of LAO at f_1=7.96 THz (left panel) and f_2=8.07 THz (right panel). In both cases, the contours remain closed and elliptical, confirming that the observed contrast inversion takes place within the elliptical polaritonic regime.

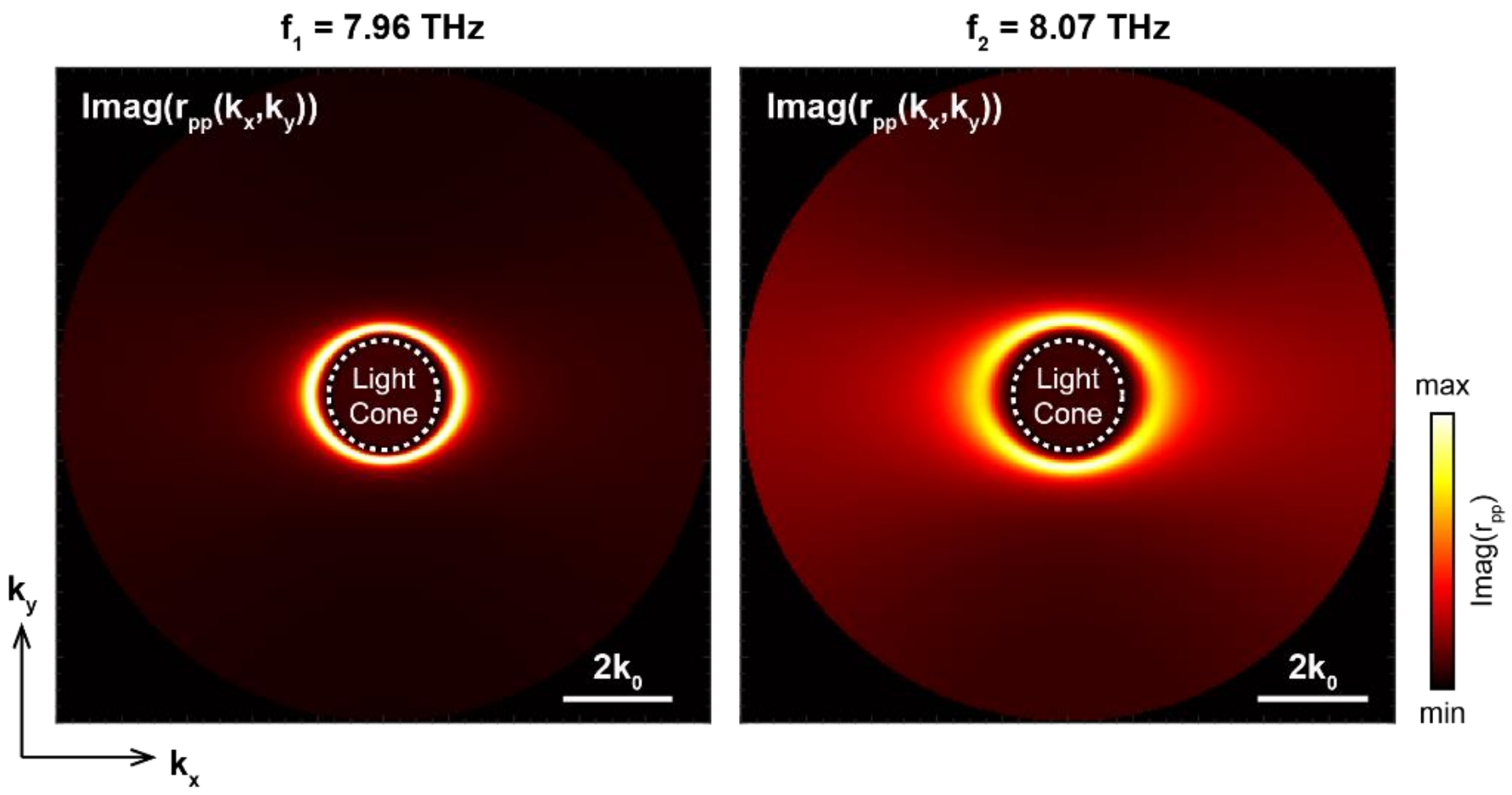

Figure S17. Calculated in-plane isofrequency contours of LAO at f_1=7.96 THz (left panel) and f_2=8.07 THz (right panel). Because the LAO optical axis is tilted with respect to the surface normal of the (001) plane, the projected in-plane polaritonic dispersion becomes anisotropic. Consequently, the in-plane isofrequency contours in the (001) plane deviate from the isotropic response expected for a uniaxial material whose optical axis is aligned with the surface normal. Both contours are closed and elliptical, demonstrating that the experimentally observed bright/dark contrast inversion at the twin walls occurs within the in-plane elliptical polaritonic regime and is therefore not caused by an elliptical-to-hyperbolic topological transition of the polariton dispersion.

Supplementary Section S8: Comparison between FEM simulations and experiment at f_1 and f_2

In the main text, the FEM simulations shown in Fig. 2f,g were performed at f_1'=8.06 THz and f_2'=8.24 THz. These frequencies were selected as representative cases because they reproduce the bright/dark twin-wall contrast observed in the experiments and predicted by the analytical model (see SI S6) and illustrate the contrast inversion most clearly within the point-dipole FEM framework. The point-dipole approximation is expected to reproduce the qualitative contrast mechanism, but not necessarily the exact experimental frequency at which the contrast inversion occurs. Therefore, the simulated inversion can appear shifted with respect to the experimentally observed inversion, typically in the order of 2% [12]. To directly address this point, we performed additional FEM simulations at the experimental frequencies f_1=7.96 THz and f_2=8.07 THz, shown in Fig. S18. The simulations at f_1 and f_2 display the same bright/dark twin-wall contrast as

the simulation at f_1'=8.06 THz. This confirms that the contrast calculated at f_1' is not an isolated feature of that particular frequency, but rather a robust response within this spectral window. The agreement between the simulations at f_1, f_2, and f_1', together with the analytical model, supports the interpretation that the TW contrast originates from the frequency-dependent effective polarizability of the wall region relative to the surrounding bulk domains. The fact that the experimental contrast inversion occurs between $f_1$and f_2, whereas the point-dipole FEM inversion is shifted toward f_1'–f_2', is consistent with the known limitations of the point-dipole approximation.

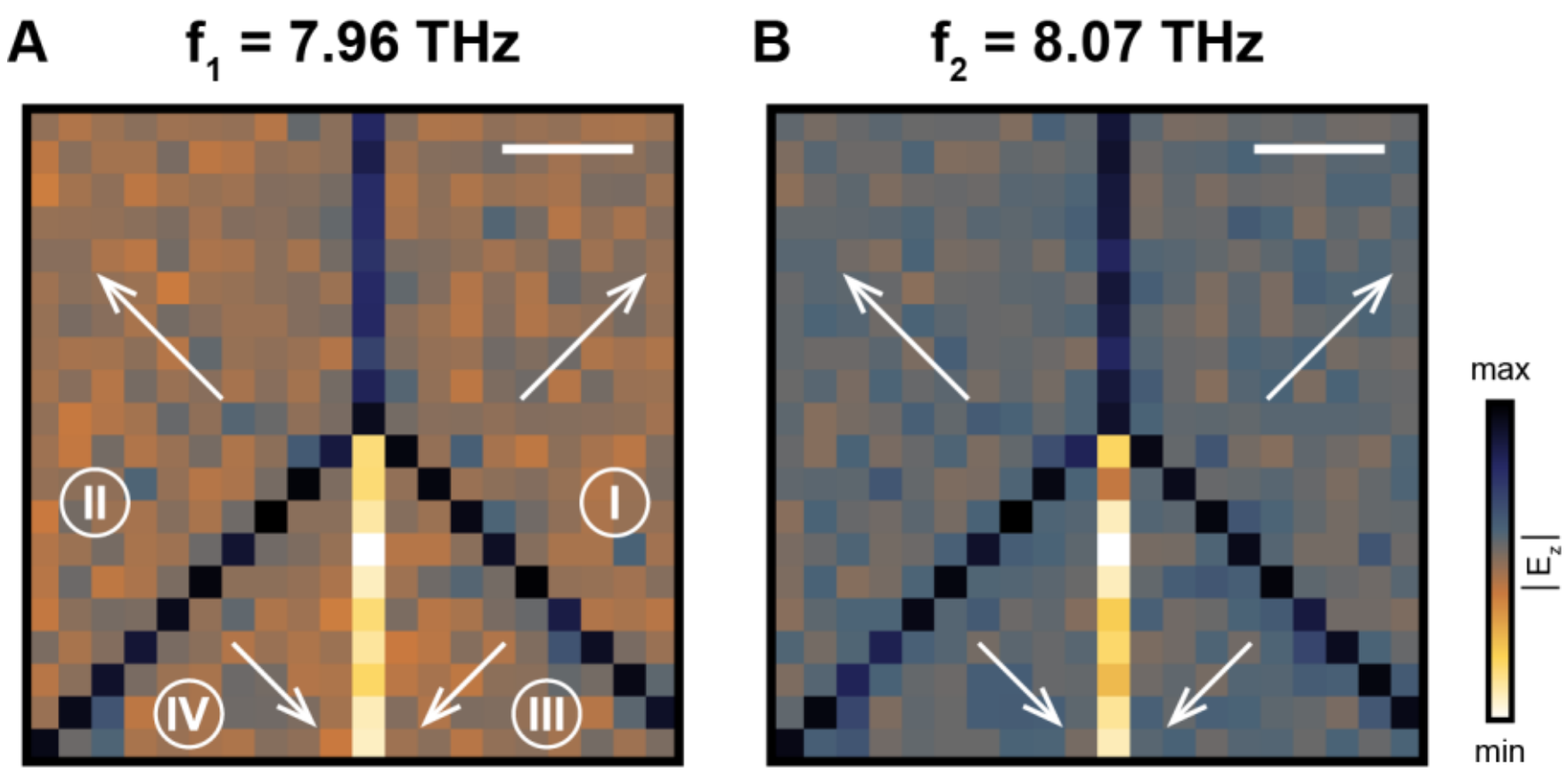

Figure S18. FEM simulations of the same TWJ as shown in Figs. 2d, e of the main text, performed at the experimental frequencies f_1=7.96 THz and f_2=8.07 THz. Panels A and B show the simulated near-field maps at $f_1$and f_2, respectively. White arrows indicate the in-plane projection of the optical axis for each domain. The domains are labelled I–IV following the notation used in the main text. Scale bars: 2 μm.

References

1 Kuzmenko, A. B.; Tishchenko, E. A.; Orlov, V. G., Transverse optic modes in monoclinic α-Bi_2O_3. *J Phys-Condens Mat* **8**, 6199 (1996).

2 Pimenov, A. et al., Anisotropic conductivity of $Nd_{1.85}Ce_{0.15}CuO_{4-\delta}$ films at submillimeter wavelengths. *Phys. Rev. B* **62**, 9822 (2000).

3 Kuzmenko, A. B., Kramers–Kronig constrained variational analysis of optical spectra. *Rev. Sci. Instrum.* **76**, 83108 (2005).

4 Huber, A.; Ocelic, N.; Kazantsev, D.; Hillenbrand, R., Near-field imaging of mid-infrared surface phonon polariton propagation. *Appl. Phys. Lett.* **87**, 81103 (2005).

5 Zhou, Y. et al., Thermal and electrostatic tuning of surface phonon-polaritons in $LaAlO_3/SrTiO_3$ heterostructures. *Nat. Commun.* **14**, 7686 (2023).

6 Hu, F. et al., Imaging exciton–polariton transport in $MoSe_2$ waveguides. *Nat. Photonics* **11**, 356 (2017).

7 Passler, N. C.; Paarmann, A., Generalized 4 x 4 matrix formalism for light propagation in anisotropic stratified media: study of surface phonon polaritons in polar dielectric heterostructures: erratum. *J. Opt. Soc. Am. B* **36**, 3246 (2019).

8 Knoll, B.; Fritz, K., Enhanced dielectric contrast in scattering-type scanning near-field optical microscopy. *Opt. Commun.* **182**, 321 (2000).

9 Feres, F. H. et al., Dipole modelling for a robust description of subdiffractional polariton waves. *Nanoscale* **11**, 21218 (2019).

10 Schneider, S. C.; Grafström, S.; Eng, L. M., Scattering near-field optical microscopy of optically anisotropic systems. *Phys. Rev. B* **71**, 115418 (2005).

11 Wehmeier, L. et al., Polarization-dependent near-field phonon nanoscopy of oxides: $SrTiO_3$, $LiNbO_3$, and $PbZr_{0.2}Ti_{0.8}O_3$. *Phys. Rev. B* **100**, 35444 (2019).

12 Cvitkovic, A.; Ocelic, N.; Hillenbrand, R., Analytical model for quantitative prediction of material contrasts in scattering-type near-field optical microscopy. *Opt. Express* **15**, 8550 (2007).

13 Bohren, C. F.; Huffman, D. R., *Absorption and Scattering of Light by Small Particles*. John Wiley & Sons, Ltd 1998.

14 Renger, J.; Grafström, S.; Eng, L. M.; Hillenbrand, R., Resonant light scattering by near-field-induced phonon polaritons. *Phys. Rev. B* **71**, 75410 (2005).

15 Narimanov, E. E., Ghost resonance in anisotropic materials: negative refractive index and evanescent field enhancement in lossless media. *Adv. Photonics* **1**, 46003 (2019).

16 Waseer, W. I.; Naqvi, Q. A.; Mughal, M. J., Non-uniform plane waves (ghost waves) in general anisotropic medium. *Opt. Commun.* **453**, 124334 (2019).

17 Ma, W. et al., Ghost hyperbolic surface polaritons in bulk anisotropic crystals. *Nature* **596**, 362 (2021).

18 Nikitin, A. Y. et al., Real-space mapping of tailored sheet and edge plasmons in graphene nanoresonators. *Nat. Photonics* **10**, 239 (2016).

19 Passler, N. C. et al., Hyperbolic shear polaritons in low-symmetry crystals. *Nature* **602**, 595 (2022).

20 Díaz-Núñez, P. et al., Visualization of topological shear polaritons in gypsum thin films. *Science Advances* **11**, eadw3452 .
