# Supplementary material for: Ultra-high THz-field-confinement at LaAlO3 twin walls
Source: Nat Commun. 2026 Sep 28;17:11212. doi: 10.1038/s41467-026-77689-5 (PMC13720113; doi:10.1038/s41467-026-77689-5)
Supplement: Supplementary file 2 [file 41467_2026_77689_MOESM2_ESM.pdf]

nature portfolio

Peer Review File

Ultra-high THz-field-confinement at $LaAlO_3$ twin walls

Corresponding Author: Dr Samuel Seddon

This file contains all reviewer reports in order by version, followed by all author rebuttals in order by version.

Version 0:

Reviewer comments:

Reviewer #1

(Remarks to the Author)
The manuscript reports on ultra-high electromagnetic field confinement at ferroelastic twin walls in $LaAlO_3$, combining scattering-type near-field optical microscopy, analytical modelling, and finite-element simulations to demonstrate frequency-dependent canalization of THz–MIR radiation without the need for nanofabrication. The study addresses a timely and important challenge in nanophotonics—namely, achieving deep-subwavelength confinement alongside long-range propagation—and proposes an intriguing paradigm based on naturally occurring domain structures in bulk crystals.
The experimental observations are compelling and are, in general, supported by theoretical analysis; however, several aspects of the work require further clarification. Provided that these issues are adequately addressed, the manuscript has the potential to make a meaningful contribution to the field. I therefore recommend publication after the authors have satisfactorily responded to the comments and questions raised below.

1. Due to spontaneous crystal symmetry breaking, the ferroelastic material LaAlO3 exhibits multiple crystallographic domains, leading to different orientations of optic axis coexisting within bulk material, even in the absence of nanostructuring. Temperature is a critical parameter governing domain formation and stability, impacting the polaritonic responses (10.1038/s41586-025-09288-1). However, the manuscript does not specify the temperature conditions under which the experiments were performed, either in the main text or in the Supporting Information. This omission is problematic, as the operational temperature directly impacts both the domain configuration and the practical applicability of the proposed confinement and canalization mechanism. If the effects reported here require elevated temperatures (e.g., on the order of several hundred °C), this would significantly limit their technological relevance. The authors should clearly state the experimental temperature conditions and discuss their implications.

2. The optical property of LAO depends on the orientation of the optic axis which is aligned with the pseudocubic crystallographic direction, as indicated in Fig.1b. Furthermore, the formations and geometry of TWs and TWJs are intrinsically linked to these orientations. Therefore, a precise and unambiguous description of the optic axis orientation is essential for understanding the reported phenomena. At present, the discussion in the section “Optical anisotropy and twin-wall pattern” remains difficult to follow, even with Fig. 1b and 1c. In particular, the spatial relationships between domain, optic axis and twin walls are not sufficiently clear. I strongly recommend that the authors provide a more detailed and explicit description, potentially supplemented by additional schematics or 3D representations in the Supporting Information, to improve clarity and accessibility.

3. The manuscript groups vertical and zig-zag TWs into two categories based on the orientation of the optic axis relation the surface, and attributes the observed bright/dark contrast to the dielectric component approaching the resonance condition($Re(\varepsilon) \approx -2$).While the modified dipole model qualitatively captures the frequency-dependent contrast inversion, the geometric descriptions remain ambiguous. Phrases such as “it points toward the wall but away from the surface” are not sufficiently precise and are difficult to interpret without a clear visual reference. The authors are encouraged to include a schematic explicitly illustrating the relative orientations of the optical axis, twin wall, and sample surface.
In addition, there appears to be a discrepancy between the experimental frequencies (f_1, f_2) and the simulation frequencies (f_3, f_4), which are not only different but also do not coincide with the condition $Re(\varepsilon) = -2$. The authors should clarify the rationale behind this frequency offset and explain how it affects the interpretation of the resonance condition and the validity of the comparison between experiment and simulation.

4. Some related works on MIR polaritons may support the background of this manuscript, which are suggested to be discussed, such as the importance of symmetry in polaritons (doi:10.1038/s41565-022-01264-4 and 10.1038/s41586-021-

04328-y), and a few others.

5. The primary novelty of this work lies in the reported ultra-high electromagnetic field confinement in the THz spectral regime. However, the manuscript currently lacks a quantitative comparison with prior studies in terms of confinement performance. Without such benchmarking, it is difficult to assess the relative advancement and significance of the reported results within the broader context of the field. I therefore encourage the authors to include a comparative table summarizing key metrics (e.g., confinement factor, propagation length, operating frequency range, and material platform) alongside representative state-of-the-art works. This would greatly enhance the clarity of the manuscript and more convincingly demonstrate the advantages of the present approach.

Reviewer #2

(Remarks to the Author)
In this manuscript, the authors show that ferroelastic twin walls - crystallographically perfect 2D-sheets that separate regions of differently oriented domains – in the distorted perovskite LaAlO3 provide a natural platform for lateral confinement and guiding of THz light at the nanoscale. Without fabrication processes, the electromagnetic fields localized at such walls exhibit lateral optical sizes up to 260 times smaller than the free-space wavelength. The results were experimentally demonstrated and numerically confirmed. This study is comprehensive. However, the following concerns should be addressed before the manuscript can be considered for publication:
(1)The physical mechanism should be explained more clearly: As it is well known, deep-subwavelength confinement of THz or infrared light by polar crystals can be realized due to negative permittivity (at the so-called Reststrahlen bands, RBs) and the excitation of phonon modes. Here the LaAlO3 crytal is anisotropic, and for the twin walls at the surface, they can guide phonon polaritons if the permittivity in one crystallographic domain is negative and the permittivity contrast at different domains of the twin walls is large enough, just like edges of metallic triangle waveguides guiding surface plasmons.
(2)The authors use the Mie resonance condition to explain the optical contrast mechanism at the walls (different walls brighten or darken at different frequencies). Mie resonance condition is used for localized resonances of sphere particles. And it is incorrectly used here to explain the excitation of propagating phonon polaritons. A detailed analysis based on the physical mechanism above may give the correct answer.

Reviewer #3

(Remarks to the Author)
This work proposed a ferroelastic material platform for confinement of MIR-THz light at ferroelastic twin walls (TWs). Under appropriate conditions, light can be highly confined along TWs, presenting canalization-like phenomena. The effect of adjacent domain orientation and operation frequency on the contrast inversion phenomenon is explored. The topic is of potential interest. But the quality does not reach the publication standard of Nature Communications. My major comments are as follows:

1. Canalization phenomenon has been widely reported within the PhP community via stacking hyperbolic slabs [Nature 582, 209 (2020)], metasurfaces [Nat. Commun. 11, 3663 (2020)], and natural crystals [Optica 12, 343 (2025); https://doi.org/10.48550/arXiv.2604.12174]. Based on these physical configurations, it can be effectively modulated via gate voltage [Science 379, 558–561 (2023)], twisted angle [Nature 582, 209 (2020)], and achieved in broadband [Nano Lett. 25(7), 2610–2617 (2025)]. The proposed highly directional light can only transport along the TWs, which are fixed in direction and hard to modulate. Given these, what are the advantages the LaAlO3 platform offers over well-established techniques?
2. Canalization happens in homogeneous media with highly directional propagation. The highly confined energy at TWs in this work is induced by the physical configuration between two crystallographic domains with different orientations. The optical contrast at TWs is likely to come from the discontinuity of charges between adjacent domains. It is not appropriate to use the term "canalization" to define energy confinement in this work.
3. In this work, the crystallographic directions are predefined and lack tunability. What is the novelty or practical applications of the proposed architecture? How does the proposed LaAlO3 platform integrate with other on-chip optical components?
4. The near-field optical scans are measured within the Reststrahlen bands, but no obvious polariton fringes are detected. What is the reason for this? Also, will the topology (either elliptical or hyperbolic) of the in-plane isofrequency contour affect the contrast inversion?
5. In this work, the crystallographic domains are represented as long vertical (010) TWs intersected by the zig-zag TW set. It is also noted that the width along the (100) direction of domains I and II varies. Are these patterns formed naturally and fixed for a specific device? Does the pattern vary from sample to sample? And what are the physical factors that affect the domain distribution?
6. In Fig.1 and 2, the authors present RBs and permittivity along and orthogonal to the optical axis. However, the PhP modes are investigated within the (001) surface. The effective permittivity along (100) and (010) should be considered rather than simply using the optical constants along and orthogonal to the optical axis.
7. The authors report on frequency-dependent contrast inversion at TWJs and claim it is related to the Mie resonance condition. How are the PhPs at TWs coupled with the Mie resonance? A more intuitive figure and elucidation should be added to clarify the theoretical framework.
8. In the practical near-field scanning microscopy, the AFM tip for near-field information collection oscillates vertically. What is the reason that the near-field probe is presented by a small metallic sphere with isotropic polarizability in the analytical model? Please explain.
9. The authors present simulated results of frequency-dependent contrast inversion at TWJs both in Fig. 2 and Fig. 4 at f3 =

8.06 THz and f4 = 8.24 THz, while the near-field experimental validation is performed at f1 = 7.96 THz and f2 = 9.07 THz. Please explain this mismatch, and also theoretically elaborate on the physics of the contrast at f3 = 8.06 THz and f4 = 8.24 THz. The simulation results at f1 = 7.96 THz and f2 = 9.07 THz should be added and discussed.

10. The authors claim “long-range, guided near-field transport”. Towards this, an energy profile and electrical field distribution along the propagation direction, and comparison to the propagation length of other materials or physical configurations, should be added.

Version 1:

Reviewer comments:

Reviewer #1

(Remarks to the Author)
The authors have done significant revisions according to the reviewers' comments. I now see that the paper has been improved, and thus recommend the publication of this work.

Reviewer #2

(Remarks to the Author)
All my comments have been sufficiently addressed in the revised manuscript. I have no further reservations and support publication.

Reviewer #3

(Remarks to the Author)
I thank the authors for their thorough and well-considered responses to the reviewers’ comments. The corresponding revisions have been appropriately incorporated into the manuscript. I therefore recommend the manuscript for publication.

Open Access This Peer Review File is licensed under a Creative Commons Attribution 4.0 International License, which permits use, sharing, adaptation, distribution and reproduction in any medium or format, as long as you give appropriate credit to the original author(s) and the source, provide a link to the Creative Commons license, and indicate if changes were made.
In cases where reviewers are anonymous, credit should be given to 'Anonymous Referee' and the source.
The images or other third party material in this Peer Review File are included in the article’s Creative Commons license, unless indicated otherwise in a credit line to the material. If material is not included in the article’s Creative Commons license and your intended use is not permitted by statutory regulation or exceeds the permitted use, you will need to obtain permission directly from the copyright holder.
To view a copy of this license, visit https://creativecommons.org/licenses/by/4.0/

Response to the Reviews of the paper:
Ultra-high THz-field-confinement at $LaAlO_3$ twin walls

Dear Editor,

we would like to thank you for considering our draft of the manuscript titled "**Ultra-high THz-field-confinement at $LaAlO_3$ twin walls"** for publication in your renowned journal. We appreciate the opportunity to respond to the important feedback by the reviewers, who's remarks we have answered below point-by-point. Based on the reviewers' responses, we incorporated all changes in our manuscript. We believe that their feedback helped to improve this paper significantly, and that our draft fits now all requirements as needed for publication.

With kind regards,
Jakob Wetzel, Javier Taboada-Gutiérrez and Sam Seddon, on behalf of all coauthors.

Summary Reviewer 1:

The manuscript reports on ultra-high electromagnetic field confinement at ferroelastic twin walls in $LaAlO_3$, combining scattering-type near-field optical microscopy, analytical modelling, and finite-element simulations to demonstrate frequency-dependent canalization of THz–MIR radiation without the need for nanofabrication. The study addresses a timely and important challenge in nanophotonics—namely, achieving deep-subwavelength confinement alongside long-range propagation—and proposes an intriguing paradigm based on naturally occurring domain structures in bulk crystals.
The experimental observations are compelling and are, in general, supported by theoretical analysis; however, several aspects of the work require further clarification. Provided that these issues are adequately addressed, the manuscript has the potential to make a meaningful contribution to the field. I therefore recommend publication after the authors have satisfactorily responded to the comments and questions raised below.

Response:
We value the Reviewer's summary, which captures the idea of our work very precisely. We appreciate the approbation of our study and the recommendation for publication. Below we answer the raised questions in a point-by-point fashion.

<u>Question 1.1:</u>

Due to spontaneous crystal symmetry breaking, the ferroelastic material LaAlO3 exhibits multiple crystallographic domains, leading to different orientations of optic axis coexisting within bulk material, even in the absence of nanostructuring. Temperature is a critical parameter governing domain formation and stability, impacting the polaritonic responses (10.1038/s41586-025-09288-1). However, the manuscript does not specify the temperature conditions under which the experiments were performed, either in the main text or in the Supporting Information. This omission is problematic, as the operational temperature directly impacts both the domain configuration and the practical applicability of the proposed confinement and canalisation mechanism. If the effects reported here require elevated temperatures (e.g., on the order of several hundred °C), this would significantly limit their technological relevance. The authors should clearly state the experimental temperature conditions and discuss their implications.

Answer 1.1: This is an essential point, and truly an oversight from us not to have included a little more discussion and clarity around these measurements. In particular, several investigations

into $LaAlO_3$, alongside a wide variety of other ferroelastic materials, have often been dominated by temperature investigations, so readers would likely be expecting something of the same. However, all measurements in the present work were conveniently performed at room temperature and in air, a fact we should indeed emphasise in the manuscript due to its significant relevance to applications.

Action 1.1: We have added during the introduction of the results section the phrase "at room temperature under ambient conditions" (on page 4, line 8) as well as "at room temperature" at the end of the introduction summary (page 2, line 29) and conclusion (page 9, line 16).

<u>Question 1.2:</u>

The optical property of LAO depends on the orientation of the optic axis which is aligned with the pseudocubic crystallographic direction, as indicated in Fig. 1b. Furthermore, the formations and geometry of TWs and TWJs are intrinsically linked to these orientations. Therefore, a precise and unambiguous description of the optic axis orientation is essential for understanding the reported phenomena. At present, the discussion in the section "Optical anisotropy and twin-wall pattern" remains difficult to follow, even with Fig. 1b and 1c. In particular, the spatial relationships between domain, optic axis and twin walls are not sufficiently clear. I strongly recommend that the authors provide a more detailed and explicit description, potentially supplemented by additional schematics or 3D representations in the Supporting Information, to improve clarity and accessibility.

Answer 1.2: We thank the reviewers for addressing the lack of clarity of domain geometry and optical axis orientation. We agree that these aspects are essential to understand the observed optical effects at the LAO twin walls. We have made the adjustments outlined below that will hopefully improve the initial introduction of the optical axis.

Action 1.2: The discussion of Figure 1b/1c in the main text has been rephrase to enhance clarity (page 2, line 52 to page 3, line 29), and two additional figures (Fig.S17, S18) have been added to the supplementary information (section S7) as reproduced below:

Changed section in main text:

"As all ferroelastic crystals, LAO forms multiple symmetry-related crystallographic domains. On the LAO(001) surface, four domain variants are allowed to form. These can be visualised by taking a cube distorted along its [111] direction (pink domain labelled "I" in Fig. 1b) and rotating it in-plane around the [001] axis three successive times by 90°. This generates domains II, III, and IV, with their respective optical axes oriented along $[\bar{1}11]$, $[\bar{1}\bar{1}1]$, and $[1\bar{1}1]$. These four domains are separated by four distinct TWs (Fig. 1c) [23, 34], that are determined by crystal symmetry and strain compatibility [38], forming exact mirror planes for the adjacent domains' crystal structures. The resulting domain tessellation forms the characteristic "chevron" tiling commonly observed in ferroelastic crystals [23, 39-41]. The TW planes are all oriented strictly orthogonal to the (001) surface and extend through the full sample thickness; on the surface, however, they follow several crystal directions, revealing two distinct types of TWs: long vertical TWs aligned with the [010] axis, and 45° zig-zag TWs running along the [110] / $[1\bar{1}0]$ axes, shown in Fig. 1c. Consequently, adjacent domains arrange themselves in alternating pairs, namely, I-III and II-IV in vertical directions. The TWs meet at twin-wall

junctions (TWJs), where all four domains and all four walls intersect (see dashed boxes in Fig. 1c).

This specific spatial configuration of domains in LAO(001) has two critical consequences for the 3D orientation of the optical axes: Firstly, the out-of-plane components of the optical axes are identical across all domains. In contrast, the in-plane projections of adjacent domains either orientate parallel or orthogonal to one another, depending on the TW type (zig-zag or vertical TWs, respectively). In Fig. 1c the projections of the optical axes onto the (001) surface are indicated as grey arrows, with arrowheads pointing out of the plane to illustrate their 3D orientations. Secondly, at each TW, the orientation of the optical axis of adjacent domains is mirrored at the TW-plane Consequently, when crossing a vertical [010] TW (such as those between I and II, or III and IV) the optical axis rotates 90 degrees, but when crossing a diagonal, zig-zag wall (like those between I and III or II and IV) the optical axis is rotated 180 degrees, so the plane the optical axis operates along is unchanged in this latter case. Note that independent of their in-plane orientation, the inclination direction of the optical axis towards the sample surface flips at each of the TWs due to the mirror symmetry, resulting in the "head-to-head" and "tail-to-tail" configurations as indicated by the grey arrows in Fig. 1c. A more detailed description of the domain formation and its optical axis orientation along with a depiction of all possible TW configurations is given in the supplementary section S7."

Supplementary Section S7: Formation of ferroelastic domains in LAO

A LAO(001) crystal can possess four different domains, each consisting of a pseudocubic unit-cell, with a compression axis along the [111] or its symmetrically equivalently directions, see Fig. S17 a). The unit cell of any given domain shares identical boundary faces with two other domains. Specifically, domain I shares one matching face with domain II (Fig. S17 b)) and its unit-cell cut diagonally fits perfectly to the diagonal cut of domain III (Fig. S17 c)); this pairwise structural compatibility is replicated across all four domains. This allows the formation of four strain-free interfaces, so called twin walls (Fig S17 d)) and the domains tesselate in a characteristic chevron pattern, seen in Fig S17 e, f).

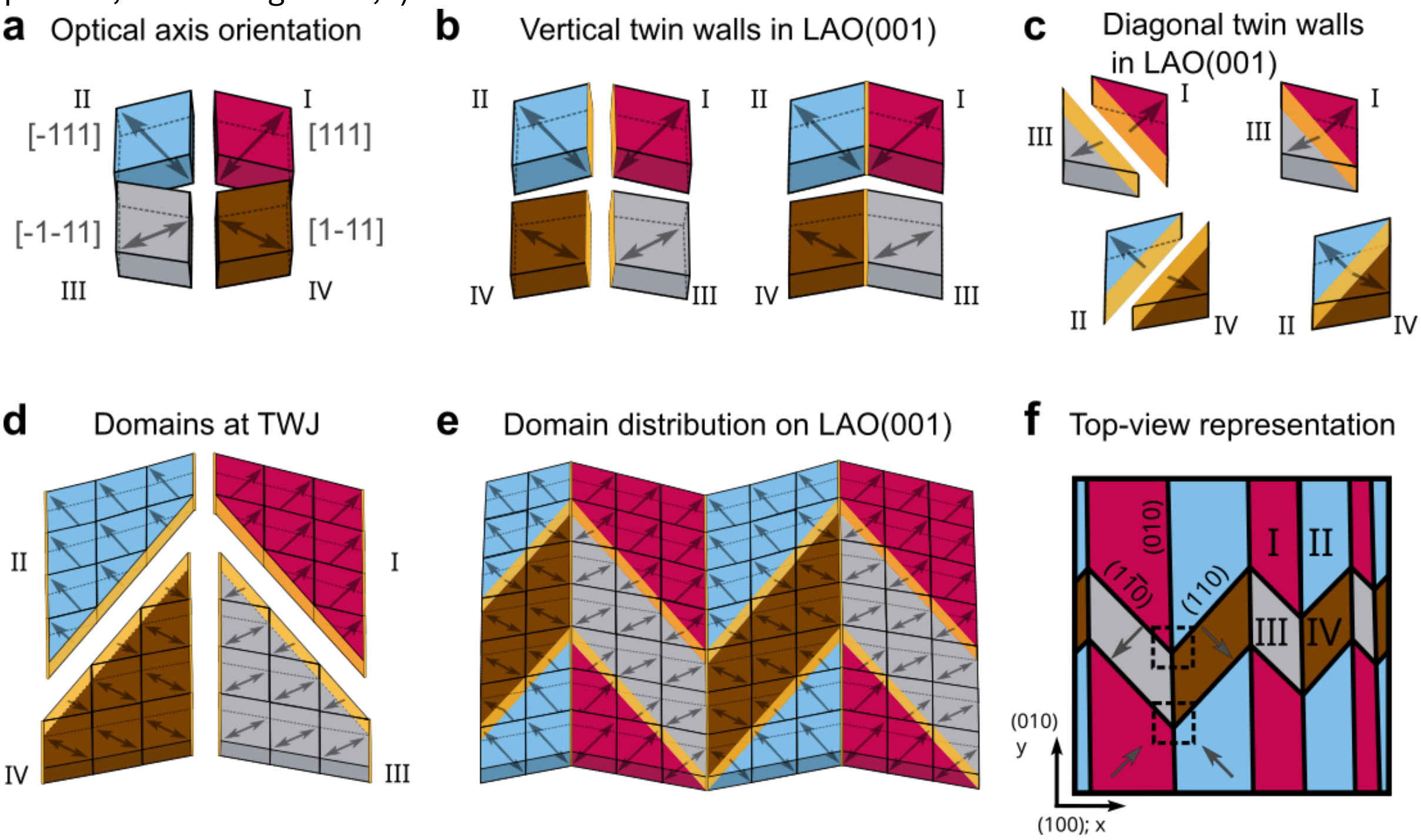

Figure S17: Schematic representation of the relation between pseudocubic unit-cells and domain tessellation. a) Pseudocubic unit-cells and their optical axis orientation of the four possible domians. Each

unit-cell is a cube distorted along their optical axes, which is aligned with the [111] axis for domain I (pink cube), and rotated by 90° three times for domains II-IV. b), c) Highlighted faces of the unit-cells, which form the strain free [b)] vertical TW between domains I and II as well as domains III and IV, and [c)] 45° TWs between domains I and III, as well as domains II and IV. d), e) Schematic representation of the four domains consisting of their unit-cells d) meeting at a TWJ, and e) tessellating the (001) plane, with f) the top-view representation used in the main text Fig. 1c).

To further clarify the relation between TWs, domains and their optical axis, Fig. S18 a) shows a schematic representation of a LAO bulk crystal with all TWs and Fig18b) the orientation of the optical axis for each type of TW.

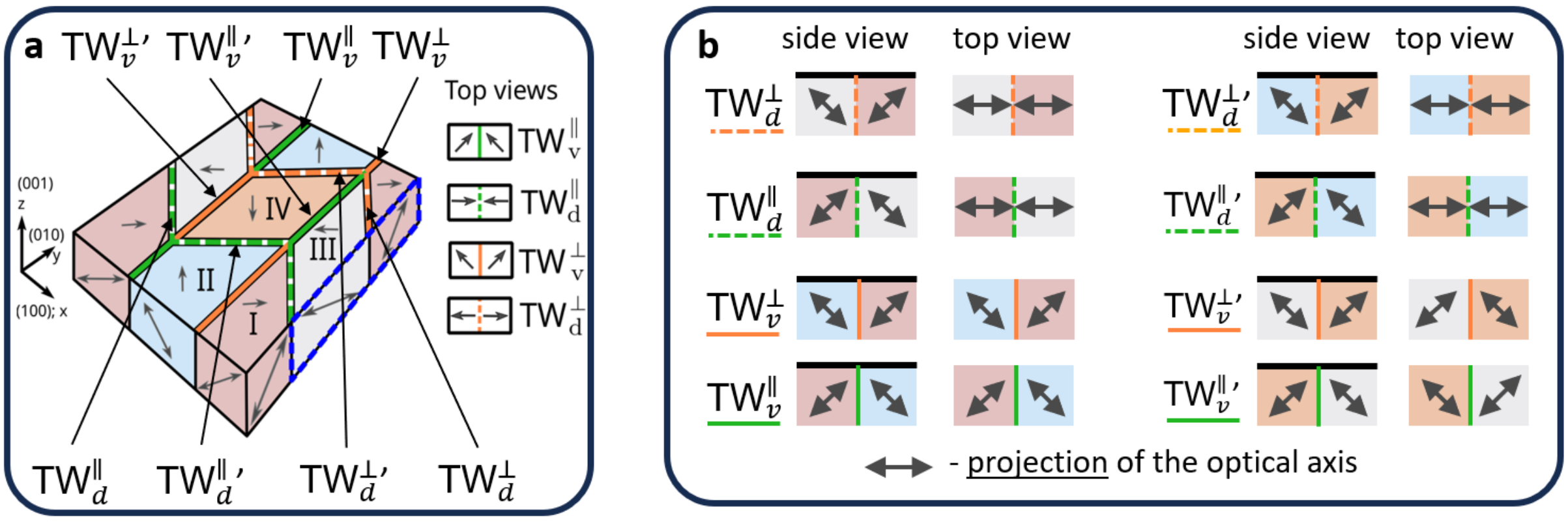

Figure S18: Orientation of optical axes for all possible TWs. Schematic representation of a) a LAO bulk crystal with the four permitted domains, separated by TWs, forming the characteristic chevron pattern, and b) top and side view of all possible TWs, with grey arrows indicating the projection of the optical axes.

Question 1.3:

The manuscript groups vertical and zig-zag TWs into two categories based on the orientation of the optic axis relation the surface, and attributes the observed bright/dark contrast to the dielectric component approaching the resonance condition (Re(ε) ≈ −2). While the modified dipole model qualitatively captures the frequency-dependent contrast inversion, the geometric descriptions remain ambiguous. Phrases such as "it points toward the wall but away from the surface" are not sufficiently precise and are difficult to interpret without a clear visual reference. The authors are encouraged to include a schematic explicitly illustrating the relative orientations of the optical axis, twin wall, and sample surface.
In addition, there appears to be a discrepancy between the experimental frequencies (f_1, f_2) and the simulation frequencies (f_3, f_4), which are not only different but also do not coincide with the condition Re(ε) = −2. The authors should clarify the rationale behind this frequency offset and explain how it affects the interpretation of the resonance condition and the validity of the comparison between experiment and simulation.

Answer 1.3: We thank the Reviewer for pointing out that the description of the geometric relation between the optical axis at TWs and the resultant contrast is hard to follow. Changes to the respective section have been added (see also answer to question 1.2), improving readability and providing a clear picture of the relative orientations of optical axes, TWs, and sample surfaces.

The discrepancy in Fig. 2c between the experimental frequencies f_1, f_2, the simulation frequencies f_3, f_4, and the frequencies where maximum contrast is expected according to the adapted dipole model (Re(ε) ≈ −2), result on one hand from the approximations made in the different theoretical models and on the other hand from technical experimental constrains. Different frequencies

were experimentally measured in the relevant spectral regime. However, due to limited available measuring time during the FEL-beamtime and the fact that within the range of 7.8 to 8.3 THz multiple lasing gaps exist, hence not every frequency could be measured. The displayed frequencies f_1, f_2 are those where the highest contrast could be observed experimentally when measurement limitations were taken into account.

Similarly, the response at different frequencies have been studied using FEM-simulations and the frequencies with the maximal contrast selected, resulting in shifted frequencies f_3 and f_4. This general shift between experiment and simulations using the point dipole approximation is well known in literature [Cvitkovic et al, Optics Express 2007, 10.1364/OE.15.008550]. The reported discrepancy is about 20 cm^{-1}, or roughly 2%, which is consistent with the observed shift in our work. We agree with the reviewer, that this difference in experimental frequencies f_1, f_2 and those used for FEM-simulations (f_3, f_4) has to be emphasised more in the manuscript.

Furthermore, please note that the dipole model is only employed to demonstrate the expected optical response qualitatively and especially considering its simplicity, and major assumptions, the marked frequencies in Fig. 2c at $Re(\varepsilon) \approx -2$ are only indicators where the maximum of the contrast might be expected. The exact spectral position of the near-field resonance is depending on additional parameters such as the mean tip-sample distance, oscillation amplitude and spectral width of the excitation, and is typically found for Re(ε) -3 to -1. In addition, the permittivity data used for the calculations were determined via conventional FTIR measurements on another sample ([110]-cut, see section S1) and a red-shift between resonance frequencies of macroscopic and nanoscopic measurements due to coupling has previously been observed [Zuloaga and Nordlander, Nano Letters 2011, 10.1021/nl1043242]. Moreover, the near-field resonance is likely influenced by the asymmetry in ε, resultant from LAOs anisotropy. Nevertheless, these analytical predictions match the observed frequency behaviour surprisingly well, and therefore have been included in our work. In our initial manuscript, it seems that the marking of the Mie-resonance condition in Fig. 2c has unintentionally emphasised this type of resonance character. We therefore adapted these figures and the corresponding text in the revised manuscript to now highlight the resonance regime typically observed in SNOM for Re(ε) from -3 to -1 as reported in literature.

Action 1.3: We restructured the introduction of the nomenclature for the TWs (page 6, lines 27-30) and added a geometric description of the dipole model (section S4) and a figure clarifying the relation of the optical axis at the TWs and their optical response in section S7, see also the answer to the previous question. For the sake of clarity, the following text stresses the mentioned changes:

Changes introduced into the main text:

"The grey arrows in Fig. 3a (optical-axis projections) then motivate two geometrical classes as illustrated by the top view representations: the $TW^{\parallel}$ class (i.e. $TW_v^{\parallel}$ and $TW_d^{\parallel}$) with head-to-head configuration of the optical axes and the class of $TW^{\perp}$ (i.e. $TW_v^{\perp}$ and $TW_d^{\perp}$) with tail-to-tail orientation (for details including side views and a 3D representation see SI S7)."

Changes introduced intro Supplementary Section S4:

"Our model assumes an infinitely extended sample surface located at *z = 0*, with the probe in air above (z > 0), and the sample below (*z = 0*). The TW, located at *x = 0* separates the sample half-space into two quarter spaces (see Fig. S12A). The two adjacent sample domains are then represented by two laterally separated polarizable spheres, here placed at *z = −h* and *x = ±h,* with in general arbitrarily oriented anisotropic permittivity tensors. Similar to the conventional dipole-model, the tip is assumed to be represented by an isotropic sphere and is placed at a height *z = h*, directly above the TW-surface junction (*x = 0*), i.e. symmetrically between the two sample spheres (see Fig. S12A)."

Furthermore, we emphasised the discrepancy between experiment and simulations (Dipole model + FEM) and the limitations of the point dipole model (page 5, lines 25-27 and page 6, lines 13-15). We removed reference to Mie-like resonance from Fig. 2c, and highlighted instead the SNOM resonance regime from $\mathrm{Re}(\varepsilon) = -3$ to $\mathrm{Re}(\varepsilon) = -1$. These changes are reproduced here for the sake of clarity:

Changes in page 5:
"The spectral shift of the simulations towards slightly higher frequencies compared to the experimental results is consistent with the frequency shift previously reported for s-SNOM simulations utilising the point-dipole approximation [49]."

Changes in page 6:
"In order to qualitatively discuss why different walls brighten or darken at different frequencies, the analytical point-dipole model commonly used to describe s-SNOM contrast was adapted [42-44, 50]."

Question 1.4:
Some related works on MIR polaritons may support the background of this manuscript, which are suggested to be discussed, such as the importance of symmetry in polaritons (doi:10.1038/s41565-022-01264-4 and 10.1038/s41586-021-04328-y), and a few others.

Answer 1.4: We appreciate the recommendation to discuss the importance of symmetry. As pointed out in the cited papers, high confinement without fabrication can be achieved in natural crystals with reduced symmetry. Low symmetry crystals display frequency-dependent optical axes and asymmetric light propagation and dissipation of energy, due to the non-orthogonality of the phonon modes. LAO, however, is a uniaxial material with the optical axis aligned with the [111] direction, supporting optical phonons that are degenerate in the directions perpendicular to the optical axis, and therefore it does not display any of the above-commented phenomena. The tilted optical axis of LAO can support ghost-like polaritonic fields [10.1117/1.AP.1.4.046003; 10.1016/j.optcom.2019.124334], i.e. non-uniform polaritonic modes with simultaneous phase propagation and spatial decay even in the lossless limit. This ghost character originates from the anisotropic dielectric response and should be distinguished from ordinary absorption-induced damping. Furthermore, the tilting of the LAO optical axis is particularly relevant because it can make the polaritonic field distribution asymmetric and direction-dependent.
Indeed, in this work, we demonstrate the excitation of surface polaritons at the (001) surface of LAO (see Figure S9) launched by an Au bar by means of SINS spectroscopy. Through transfer matrix calculations (see the new Supplementary Section S5 and Figure Response 1), we demonstrate that the tilting of the optical axis induces an anisotropic response of the in-plane dispersion of those polaritons, in contrast with other materials whose surface is aligned with the optical axis, such as h-BN.

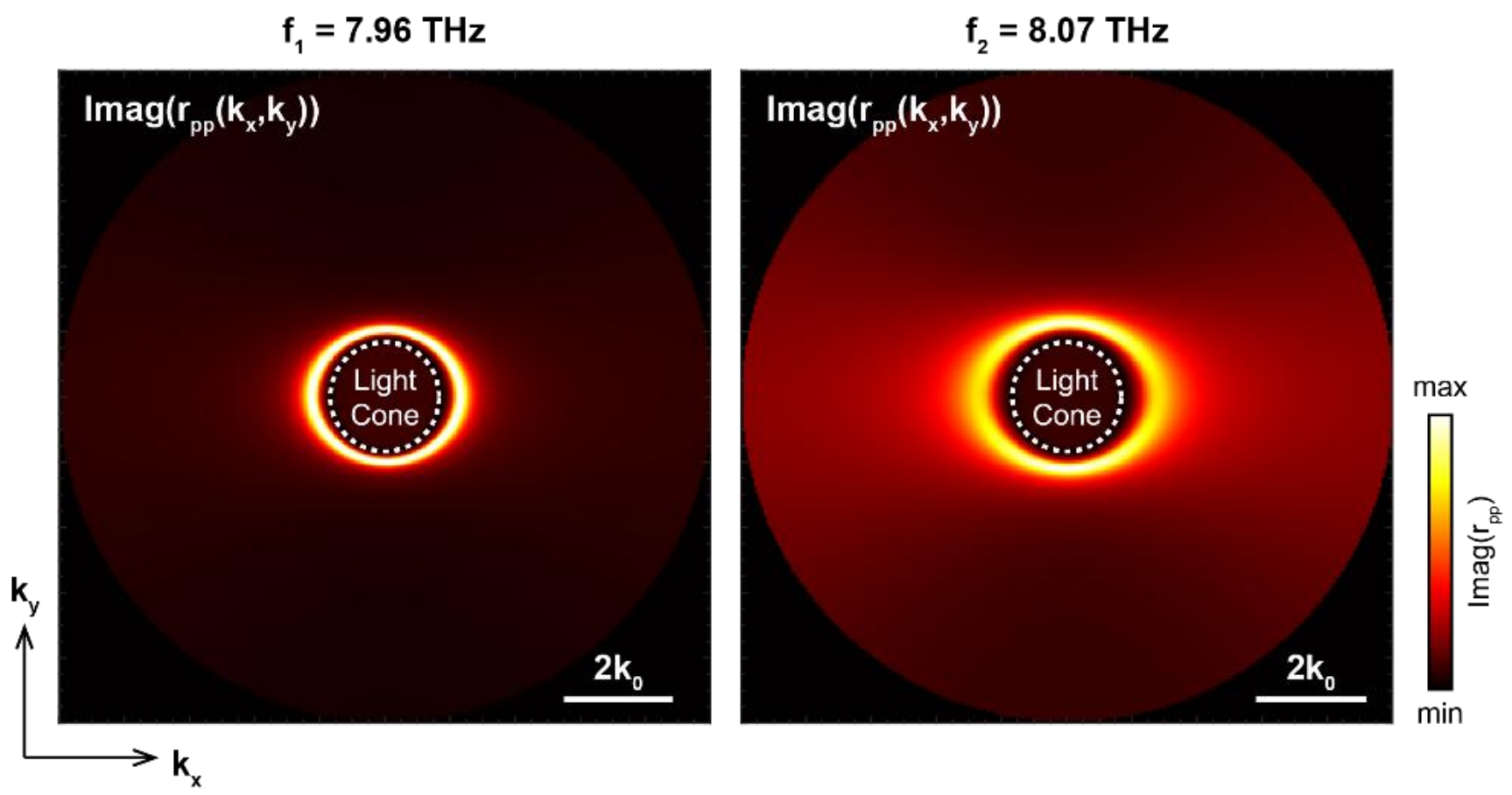

Figure Response 1. Calculated in-plane isofrequency contours of LAO at f_1=7.96 THz (left panel) and f_2=8.07 THz (right panel). Because the LAO optical axis is tilted with respect to the surface normal of the (001) plane, the projected in-plane polaritonic dispersion becomes anisotropic. Consequently, the in-plane isofrequency contours in the (001) plane deviate from the isotropic response expected for a uniaxial material whose optical axis is aligned with the surface normal.

Action 1.4: Supplementary Section S5 has been added commenting on the symmetry of the system and the in-plane dispersion relation of surface phonon polaritons at the LAO (001) surface and reproduced here for the sake of clarity:

Supplementary Section S5: Role of the crystal symmetry on the in-plane isofrequency contour and on the twin-wall contrast inversion.

Due to the tilting of the optical axis with respect to the (001) LAO surface, the material can potentially support ghost-polariton-like solutions [15, 16, 17]. In such modes, the wavevector can acquire simultaneous real and imaginary components even in the absence of material loss, leading to non-uniform polaritonic fields that both oscillate and decay in space. Therefore, the ghost character originates from the complex-wavevector structure imposed by the anisotropic dispersion, rather than from absorption alone. This situation should be distinguished from hyperbolic shear polaritons in low-symmetry monoclinic or triclinic crystals [18, 19, 20]. In those systems, the shear response arises from non-orthogonal phonon modes and from off-diagonal components of the dielectric tensor that cannot be fully removed by a frequency-independent rotation. As a result, the optical axes themselves disperse with frequency, producing a frequency-dependent response of the polariton propagation direction, tilted wavefronts, asymmetric energy flow, and, in some cases, topology changes between hyperbolic, canalised, and elliptical regimes. In LAO, by contrast, the material is uniaxial: the dielectric response is fully described by $\varepsilon_\parallel$ and $\varepsilon_\perp$, and the apparent in-plane anisotropy arises from rotating this tensor into the (001) laboratory frame. Consequently, ghost-polariton-like behavior in LAO should not be interpreted as a shear-polariton response of a low-symmetry crystal. Depending on frequency and on the orientation of the tilted optical axis relative to the propagation direction, the projected in-plane dispersion may be elliptical or hyperbolic. Thus, ghost-polariton-like fields can exist in each of the RBs, whereas the classification as hyperbolic or elliptical refers to the topology of the in-plane isofrequency contour

On the other hand, the contrast inversion of the near-field signal at LAO twin walls occurs close to the transition between two polaritonic regimes. Below approximately 8.24 THz (ω_0=275 cm^{-1}), LAO exhibits an elliptical in-plane isofrequency contour, with both $\varepsilon_\perp$ and $\varepsilon_\parallel$ being negative but with different absolute value. Above this frequency, LAO enters a hyperbolic regime, where $\varepsilon_\parallel$ becomes positive while $\varepsilon_\perp$ remains negative. However, the experimentally observed twin-wall contrast inversion occurs between f_1=7.96 THz and f_2=8.07 THz, i.e. below the onset of the

hyperbolic regime. Therefore, the inversion cannot be attributed to an elliptical-to-hyperbolic topological transition of the in-plane polariton dispersion. Instead, the inversion arises from the frequency-dependent local near-field response of the twin walls relative to the surrounding bulk LAO domains. Figure S15 shows the calculated in-plane isofrequency contours of LAO at f_1=7.96 THz (left panel) and f_2=8.07 THz (right panel). In both cases, the contours remain closed and elliptical, confirming that the observed contrast inversion takes place within the elliptical polaritonic regime.

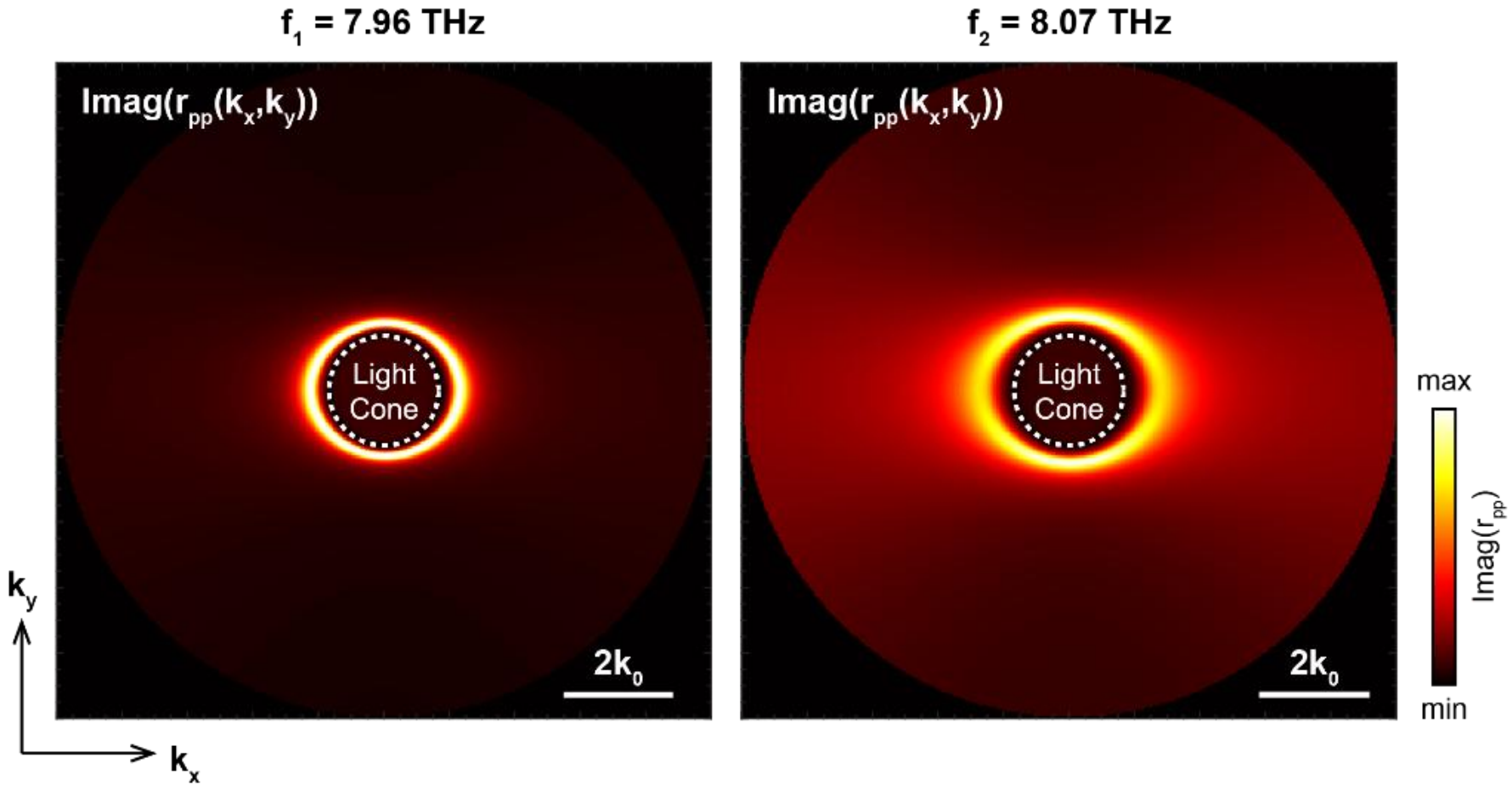

Figure S15. Calculated in-plane isofrequency contours of LAO at f_1=7.96 THz (left panel) and f_2=8.07 THz (right panel). Because the LAO optical axis is tilted with respect to the surface normal of the (001) plane, the projected in-plane polaritonic dispersion becomes anisotropic. Consequently, the in-plane isofrequency contours in the (001) plane deviate from the isotropic response expected for a uniaxial material whose optical axis is aligned with the surface normal. Both contours are closed and elliptical, demonstrating that the experimentally observed bright/dark contrast inversion at the twin walls occurs within the in-plane elliptical polaritonic regime and is therefore not caused by an elliptical-to-hyperbolic topological transition of the polariton dispersion.

Question 1.5:

The primary novelty of this work lies in the reported ultra-high electromagnetic field confinement in the THz spectral regime. However, the manuscript currently lacks a quantitative comparison with prior studies in terms of confinement performance. Without such benchmarking, it is difficult to assess the relative advancement and significance of the reported results within the broader context of the field. I therefore encourage the authors to include a comparative table summarizing key metrics (e.g., confinement factor, propagation length, operating frequency range, and material platform) alongside representative state-of-the-art works. This would greatly enhance the clarity of the manuscript and more convincingly demonstrate the advantages of the present approach.

Answer 1.5: We thank the reviewer for this important suggestion. We agree that benchmarking the reported confinement against representative polaritonic platforms helps clarify the significance of our results. We would first like to clarify the definition of confinement used in our work. In many polaritonic studies, the confinement factor is defined as $\beta=\lambda_0/\lambda_P$, where λ_0 is the free-space wavelength and λ_P is the polariton wavelength along the propagation direction. This quantity is naturally connected with the mentioned key figures of merit (FOM) such as propagation length and quality factor. In the present case, however, our work for THz-confinement at LAO TWs focuses on a lateral confinement, perpendicular to the propagation direction of the

SPhPs, quantified by λ_0/FWHM. Hence, a comparison to the above mentioned conventional key FOMs is not possible with the experimental data reported in the manuscript.
However, we agree with the Reviewer that such a comparison is useful. In twisted MoO_3 systems, where canalization of PhPs is found at certain rotation angles, thicknesses and frequencies, experimental lateral confinement factors are on the order of 40 [10.1038/s41586-020-2359-9; 10.1021/acs.nanolett.4c04633]. By contrast, in our LAO TW system, the experimentally extracted lateral confinement is approximately 260, while FEM simulations predict a fundamental lateral confinement of 1057. We also note that the nature of the lateral confinement in LAO TWs differs from that in twisted MoO_3 systems, where the canalized PhP exhibits lateral spreading [10.1038/s41586-020-2359-9; 10.1021/acsnano.3c06477] and depends on sample thickness, while the LAO TW response is bound to crystallographically defined walls in a bulk crystal and can be observed in three narrow spectral regimes over the broad range of 7.9 to 20.7 THz.

In addition, we have performed FEM simulations to compare propagation-related quantities for LAO twin-wall polaritons with representative MoO_3-based systems, including single-layer and twisted-layer configurations. Table Response 1 summarizes the polariton wavelength, propagation length, and quality factor (Q = $Re(k_p)/Im(k_p)$) for these cases. These simulations indicate that polaritons associated with LAO twin walls are not strongly confined in terms of (λ_0/λ_p), since their polariton wavelength remains relatively close to the free-space wavelength. However, this comparatively low wavelength compression is accompanied by a longer propagation length, which is larger (an order of magnitude) than that obtained for the simulated single-layer and twisted MoO_3 cases under comparable excitation conditions showing that the field localised at the LAO twin wall persists over mesoscopic distances (L_p=46.48 μm and 27.49 μm depending on the wall) while remaining laterally confined to the wall region. The extracted quality factors are of the same order of magnitude across the different systems, except for the ($TW_v^{\perp}$) LAO case, which is approximately (50%) smaller. Furthermore, we have added some reported experimental FOMs, which are also on the same order of magnitude than the theoretical values simulated through FEM.
A full modal analysis of launched twin-wall polaritons, including experimental extraction of propagation lengths of TW polaritons launched from Au structures is the subject of ongoing work and will be presented in a future dedicated publication. As a consequence, we would like to kindly ask the referee to keep the following information confidential and not to be published in the present manuscript.

Theoretical Calculations					
Case	f (THz)	λ_p (μm)	β	L_p (μm)	Q
Single MoO_3	27.25	0.93	11.83	1.56	10.54
Twisted MoO_3	27.25	1.22	9.02	2.19	11.30
Single MoO_3	8.67	3.56	9.71	5.64	9.96
Twisted MoO_3	8.67	4.14	8.35	6.30	9.56
LAO $TW^{\perp}$	8.06	29.50	1.26	46.48	9.90
LAO $TW^{\perp}$	8.06	27.70	1.34	27.49	6.24

Experimental Reports					
Case	f (THz)	λ_p (μm)	β	Lp (μm)	Q
Twisted MoO_3 [10.1038/s41586-020-2359-9]	27.10	-	-	2.77	-
Twisted MoO_3 [10.1021/acsnano.3c06477]	8.67	6.52	5.3	-	-

Table Response 1. Comparison of key polaritonic metrics across different material platforms. For the theoretical calculations, the single- and twisted-MoO_3 flakes simulated at 27.25 THz with a thickness of 200 nm, whereas the single- and twisted-MoO_3 flakes simulated at 8.67 THz with a thickness of 50 nm. The twisted-MoO_3 simulations were performed at the twist angle for which polariton canalization occurs, corresponding to flat bands in momentum space. For the experimental reports, Ref. [10.1038/s41586-020-2359-9] used a heterostructure composed of two MoO_3 flakes, each 117 nm thick, whereas Ref. [10.1021/acsnano.3c06477] used a twisted bilayer with a 125 nm-thick top flake and a 115 nm-thick bottom flake.

Action 1.5: A comparison with lateral confinements reported in previous works has been added to the main manuscript in the section "Frequency-dependent contrast inversion and experimental confinement" and reproduced here for the sake of clarity (page 4, lines 37-41):

"This value is approximately one order of magnitude larger than previously reported lateral confinement factors in highly directional polaritonic systems, which are typically around 40 [17, 21], while being realised in a bulk LAO crystal where the confinement is provided by naturally occurring ferroelastic twin walls rather than in twisted 2D materials."

Summary Reviewer 2:

In this manuscript, the authors show that ferroelastic twin walls - crystallographically perfect 2D-sheets that separate regions of differently oriented domains – in the distorted perovskite LaAlO3 provide a natural platform for lateral confinement and guiding of THz light at the nanoscale. Without fabrication processes, the electromagnetic fields localized at such walls exhibit lateral optical sizes up to 260 times smaller than the free-space wavelength. The results were experimentally demonstrated and numerically confirmed. This study is comprehensive. However, the following concerns should be addressed before the manuscript can be considered for publication:

Response:

We appreciate the summary, the valid comments and concerns, which we will consider in the following, hoping to justify the recommendation for publication.

<u>Question 2.1:</u>

The physical mechanism should be explained more clearly: As it is well known, deep-subwavelength confinement of THz or infrared light by polar crystals can be realised due to negative permittivity (at the so-called Reststrahlen bands, RBs) and the excitation of phonon modes. Here the LaAlO3 crystal is anisotropic, and for the twin walls at the surface, they can guide phonon polaritons if the permittivity in one crystallographic domain is negative and the permittivity contrast at different domains of the twin walls is large enough, just like edges of metallic triangle waveguides guiding surface plasmons.

Answer 2.1: We thank the reviewer for pointing out the similarities to guided plasmon polariton propagation along edges of metallic triangle waveguides. In these geometrical structures, the optical confinement and guiding is realised along the edges, where two interfaces between metal (with negative permittivity ($\varepsilon < 0$)) and air (positive permittivity) meet at a significant inner angle of typically 60°-30° [10.1117/12.803505, 10.1109/JLT.2010.2101582], preventing the surface plasmons to continue propagating along this interface.

Similarly to the triangle waveguides, in our case, surface polaritons (related to phonons SPhPs) are excited in the RBs of LAO (where $\varepsilon < 0$). However, in contrast to metallic triangle waveguides the LAO TWs show two most relevant differences: Firstly, at the LAO TWs the two adjacent domains <u>both</u> have negative permittivity. In fact, as the permittivity tensor is mirrored at the TW to align with the optical axis of these domains, in adjacent domains the single permittivity components are identical. Therefore, neither the mentioned positive to negative permittivity contrast nor a large enough permittivity contrast of the same sign is given. Secondly, the geometry of the system is clearly different: unlike for triangle waveguides the surface of LAO is almost flat (inner angle ~180°) with each domain surfaces being inclined with respect to the horizontal by only 0.075°. Furthermore, in our FEM-simulations we were able to reproduce the observed contrast without considering any surface tilt. Additionally, geometric confinement would not be able to explain the observed enhanced signal at ridges and a diminished signal in valleys, which can be switched when illuminating with a different frequency.

In line with this argument, we have prepared Figure Response 2 to further clarify the physical origin of the twin-wall contrast. Figure Response 2a shows the left-hand side of the simulations presented in Fig. 2f,g of the main text, which reproduce the s-SNOM contrast inversion at f_3 = 8.06 THz and f_4 = 8.24 THz. Figure Response 2b shows the corresponding electric-field distribution, ($Re(E_z)$), calculated for two different dipole positions at each frequency: one in the middle of a ferroelastic domain (upper dipole) and one directly above the twin wall (lower dipole). At both frequencies, the presence of the twin wall strongly modifies the polaritonic near field generated by the dipole. At f_3, the twin wall mainly redistributes the phase and amplitude of the polaritonic

field, resulting in a reduced near-field response along the wall. At f_4, by contrast, the field becomes strongly enhanced and spatially concentrated at the twin wall. This frequency-dependent redistribution is also reflected in the in-plane Poynting-vector maps shown in Figure Response 2c. The black solid contour lines indicate a fixed value of the Poynting-vector magnitude and are included to highlight the spatial redistribution of the in-plane energy flow for each frequency and dipole position. These calculations show that the twin wall does not behave as a simple geometrical ridge or as an interface with a large positive–negative permittivity contrast. Instead, it modifies the local polaritonic field through the rotated anisotropic dielectric response of the adjacent domains. At f_3, the in-plane energy flow is reduced along the twin wall, consistent with the dark contrast observed in the simulated s-SNOM image. At f_4, the energy-flow distribution becomes concentrated along the twin wall, consistent with the bright near-field contrast.

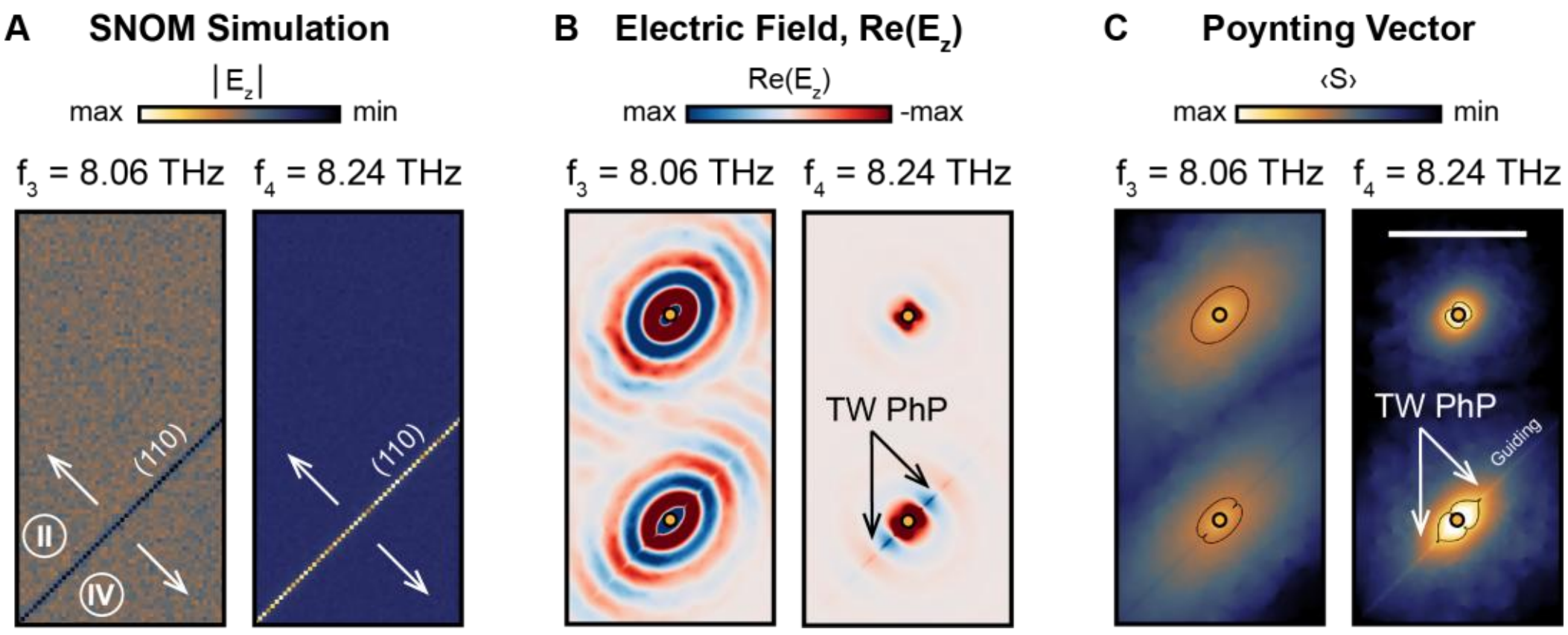

Figure Response 2. On the contrast of the TWs between f_3 and f_4. **A** FEM simulation mimicking the s-SNOM response by scanning laterally the dipole along the LAO surface and TWs. The images correspond to the left-hand side of the simulations presented in Fig. 2f,g of the main text. **B** Real part of the electric field distribution, ($Re(E_z)$), at both frequencies for two positions of the dipole: in the middle of a LAO domain (upper part of the panels) and on top of a TW (lower part of the panels). The effect of the TW on the electric field distribution is clearly visible. **C** In-plane time-averaged Poynting vector, $\langle S \rangle$, at both frequencies for the same two positions of the tip. The figures also show a contour of the distribution, demonstrating that the TW acts as a repelling channel for the lowest frequency and it guides the energy along it for the highest frequency.

Action 2.1: Added note to the manuscript in the section “FEM simulations and origin of the contrast”, on page 5, line 23, that the confinement is not geometrically-induced as observed in ridge-like modes, and reproduced here for the sake of clarity:

“Note that the simulation surface is perfectly flat, demonstrating that the contrast is driven by the local in-plane rotation of the optical axis between the neighbouring domains and not by any topography-related effect, such as observed in ridge-like modes [48].”

<u>Question 2.2:</u>

The authors use the Mie resonance condition to explain the optical contrast mechanism at the walls (different walls brighten or darken at different frequencies). Mie resonance condition is used for localized resonances of sphere particles. And it is incorrectly used here to explain the excitation of propagating phonon polaritons. A detailed analysis based on the physical mechanism above may give the correct answer.

Answer 2.2: The referee raises a legitimate point here. In the manuscript, we refer to a Mie-like resonances in the limited context of the simplistic adapted dipole model, which we developed solely to illustrate qualitatively the frequency-dependent contrast inversion via an analytical approach. The basis of this model is the quasi-electrostatic dipole model introduced by Knoll in 2000 [10.1016/S0030-4018(00)00826-9] and is therefore unable to describe propagating polaritons as pointed out by the reviewer. However, it is surprisingly well suited to describe polariton-based resonances in a near-field coupled system [Hillenbrand 2002 (doi.org/10.1038/nature00899), Taubner (10.1364/OE.20.013173), Wehmeier (10.1103/PhysRevB.100.035444)], which we explain in more detail in the following paragraphs. On the other hand, the FEM calculations shown in the manuscript are free from any assumption of the presence of Mie-resonances and thereby can include possible polariton propagation.

In the adapted dipole model developed during this work, we assumed that both the induced dipole of the tip, as well as the coupled sample dipoles are small compared to the exciting wavelength, hence being representable by point dipoles induced in small spherical particles showing indeed Mie-like resonances in the electrostatic limit. This approximation follows the classic analytical approach introduced by Knoll in 2000 [10.1016/S0030-4018(00)00826-9]. To introduce the sample in this model, the tip is typically placed above an infinitely extended sample. The tip dipole induces an image dipole within the sample and the dipole-dipole interaction is calculated. In our adapted dipole model, the sample half-space is split into two parts, each quarter-space representing a domain and the tip dipole is located exactly above the resulting interface. The discontinuity within the sample is represented by two anisotropic, induced dipoles, approximated as small spheres (leading again to Mie-like resonances). This approach has been chosen as the highly symmetric geometry of the sample spheres enables the analytical introduction of arbitrary anisotropy in the two domains. As in the standard dipole model, the tip excites polarisation in the small volume of the sample in close proximity to the tip, inducing local dipoles (now one in each domain instead of only one in the entire sample). This leads to near-field coupling of tip and samples, which can be analytically calculated and that shows Mie-like resonances in the near-field coupled system due to field localisation below the tip. Note that, similarly to the standard point-dipole model introduced by Knoll et al., this near-field resonance is strongly dependent on the tip-sample distance, typically covering spectral regimes where $Re(\varepsilon)$ = -3...-1 (Wehmeier (10.1103/PhysRevB.100.035444), Taubner (10.1021/acs.nanolett.3c03369), Kehr (10.1103/PhysRevLett.100.256403)) for extended samples, corresponding to a polariton-enhanced near-field signal even in this electrostatic model.

In conclusion, the Mie-like character of the resonance is rather a starting point to describe the localised fields and the near-field coupling in SNOM. In fact, the exact spectral position of the near-field resonance depends on additional parameters such as the tip-sample distance. In our initial manuscript, it seems that the marking of the Mie-resonance condition in Fig. 2C has been unintentionally emphasising this resonance character. We therefore adapted this figure in the revised manuscript to now highlight the resonance regime typically observed in SNOM for $Re(\varepsilon)$ = -3...-1 as reported in literature.

Action 2.2: We extended the discussion of the adapted dipole model and its limitations in the SI, emphasised in main text that the adapted dipole model is used to illustrate the contrast and is not a perfect model (page 6, lines 13-15), and refer for a point-to-point discussion of its limitations to the SI (page 7, lines 3-5). Moreover, we removed reference to Mie-resonance condition from Fig. 2c, and instead highlighted the known resonance regime in SNOM from $\text{Re}(\varepsilon) = -3$ to $\text{Re}(\varepsilon) = -1$. These changes are shown here, for the sake of clarity:

Changes in page 6:
"In order to qualitatively discuss why different walls brighten or darken at different frequencies, the analytical point-dipole model commonly used to describe s-SNOM contrast was adapted [42-44, 50]."

Changes introduced in page 7:
"... are required, such as the FEM-simulation carried out within this work. For more details on the adapted dipole model and its limitations, please see SI S4."

Summary Reviewer 3:

This work proposed a ferroelastic material platform for confinement of MIR-THz light at ferroelastic twin walls (TWs). Under appropriate conditions, light can be highly confined along TWs, presenting canalization-like phenomena. The effect of adjacent domain orientation and operation frequency on the contrast inversion phenomenon is explored. The topic is of potential interest. But the quality does not reach the publication standard of Nature Communications. My major comments are as follows:

Response:

We thank the reviewer for pointing out the potential interest of our work and for raising many important remarks. We will address those in the following and the revised manuscript, hence improving its quality significantly in order to meet the standards for publication in Nature Communications.

Question 3.1:

Canalisation phenomenon has been widely reported within the PhP community via stacking hyperbolic slabs [Nature 582, 209 (2020)], metasurfaces [Nat. Commun. 11, 3663 (2020)], and natural crystals [Optica 12, 343 (2025); https://doi.org/10.48550/ arXiv.2604.12174]. Based on these physical configurations, it can be effectively modulated via gate voltage [Science 379, 558–561 (2023)], twisted angle [Nature 582, 209 (2020)], and achieved in broadband [Nano Lett. 25(7), 2610–2617 (2025)]. The proposed highly directional light can only transport along the TWs, which are fixed in direction and hard to modulate. Given these, what are the advantages the LaAlO3 platform offers over well-established techniques?

Answer 3.1: We thank the reviewer for compiling several references that we overlooked in the preparation of the manuscript. In our answer to this question, we also address the 3rd question of the same referee. The answer comes in two parts, one addressing the novelty of the underlying physical mechanisms and another touching upon tunability.

Fundamental novelty within the community

The referee is correct that we did not emphasise in detail existence of papers exploring canalisation and localisation of PhPs. However, these papers (including the ones cited by the reviewer) are almost exclusively focused on 2D or ultra-thin layered structures and materials. More specifically:

- “Stacking hyperbolic slabs [Nature 582, 209 (2020)]” – Here the twist angle between bilayers of α-MoO_3 was investigated leading to hyperbolic, elliptical, and canalised topological polaritonic states
- “Metasurfaces [Nat. Commun. 11, 3663 (2020)]” – This work uses patterned 20-nm Hexagonal Boron nitride tuning a topological transition between hyperbolic and elliptical polaritons
- “Natural crystals [Optica12, 343 (2025); DOI: 10.48550/arXiv.2604.12174] – These two works explore phonon polariton canalisation in single layer α-MoO_3 and “a 200-nm thick flake” of α-V_2O_5, respectively.

Common amongst all these works are that they report effects present in ultra-thin or 2D materials systems and heterostructures. Our work stands distinctly apart from them as it deals with bulk crystals, utilising naturally occurring twin walls (present in a huge family of other material systems). These twin walls are not subject to fabrication, they remain perfect mirror-plane objects passing through the entirety of the sample, with the confinement we have observed due to the same mirror symmetries that stabilises the crystal structure. We are not aware of any other works which display optical effects such as these in bulk crystals.

Tunability

The mentioned lack of tunability and crystallographic pinning of the confinement are perfectly reasonable concerns that are, in part, difficult to answer or demonstrate in a single work. We would like to draw the attention of the referee to another recent work, currently submitted, and available to read on ArXiv [https://arxiv.org/abs/2604.28183]. In this work, we apply uniaxial compressive strain, and therein force the $LaAlO_3$ twin walls to (in the case of the zig-zag walls) continuously rotate their orientation or more dramatically (in the case of the vertical walls) to entirely disappear. This means that the confinement explored in the twin walls in this current manuscript can be either entirely removed, or the zig-zag twin wall angle deterministically tuned. This opens up a huge range of potential applications, such as nano-photonic logic gates, controlled via external strain.

Given the discussed novelty of such optical confinement present in bulk crystal structures, we feel that these newly reported mechanisms require their own individual work to properly establish the confinement, before the tunability and dynamics can be addressed in separate papers. In fact, experimental and theoretical confinement of this strain-tuneable field confinement are currently in preparation for publication.

Action 3.1: The references provided have been added to the main text, within the introduction section, as well as a reference to the ability to tune domain walls via strain shown in [https://arxiv.org/abs/2604.28183] (page 2, lines 24-26), and reproduced here for the sake of clarity:

"Furthermore, as part of the ferroelastic material class, it has been recently shown, that its crystal structure and domain arrangement can be controlled via applied strain [33]."

<u>Question 3.2:</u>

Canalization happens in homogeneous media with highly directional propagation. The highly confined energy at TWs in this work is induced by the physical configuration between two crystallographic domains with different orientations. The optical contrast at TWs is likely to come from the discontinuity of charges between adjacent domains. It is not appropriate to use the term "canalization" to define energy confinement in this work.

Answer 3.2: We thank the reviewer for pointing out that, within the PhP-community, the term canalisation is employed in a much narrower sense then how we initially applied it and agree that using it for the observed contrast at TWs is misleading. We removed all references of canalisation in connection with LAO TWs and focus on the phenomena of enhanced field-confinement at TWs. Furthermore, it is interesting that the referee raises the point of a likely optical contrast arising from TWs to come from a discontinuity of charges between adjacent domains, as that was one of original theories relating to the observed contrast, we had ourselves. Several experiments were performed to ultimately rule out this hypothesis, which are detailed below:

1) Piezo-response force microscopy (PFM) and Kelvin probe force microscopy (KPFM) were both used to measure the same exact twin walls and surrounding area measured for the S-SNOM data presented in the manuscript. Our research group has extensive experience with both techniques [https://arxiv.org/abs/2604.28183], in particular for the characterisation of conductively charged domain walls in other ferroic materials such as $LiNbO_3$. PFM is a contact mode atomic force microscopy technique that used an applied AC electric field to induce and locally probe piezoelectric oscillations present within the sample (most commonly arising due to ferroelectric polarisation within a probed

material). KPFM (perhaps more pertinent here) uses the contact potential difference between an atomic force microscopy tip and samples surface. This directly allows the local charge state of a system to be spatially examined.
No contrast was seen in either technique, confirming the electrostatic charge neutrality of the twin wall.

2) The observed frequency dependent inversion of the S-SNOM measured twin wall contrast indicates a phononic resonance rather than charge-related effects. Were the observed contrast to arise due to trapped charges in the twin wall, leading to different amounts and or types of charges collecting at particular twin walls and thus resulting in positive or negative (bright and dark) twin walls, no contrast inversion would be expected. However, the inversion of the effect seen as a function of frequency across all three RBs measured and ability to invert that contrast between two sets of TWs and TWJs implies, that the effect must arise from PhPs instead of from any trapped charges at the wall (which shouldn't invert in sign)
3) Charging can occur in oxide perovskites, in particular referencing the interesting recent work in oxide perovskite of Nicola Spaldin, referring to inversion of surface charges within perovskite materials [Figure 5 https://arxiv.org/abs/2102.08082], and how important they can be on the resulting $BiFeO_3$ ferroelectric overlayers. Whilst there is an argument the same polarity could exist at the pseudo-cubic surfaces or therefore indeed [010] twin walls, the (110) twin walls are by these models not charged, but rather charge neutral. Given the equivalence of effect between these two walls, indeed that the (110) zig zag walls are visible at all, implies that the potential charging that can arise at oxide perovskite interfaces is not at play here.

On the other hand, the reviewer's reference to a discontinuity of charges could also be interpreted as referring to a discontinuity of the optically induced dynamic polarization charges between adjacent domains at the twin wall. However, the two domains have the same dielectric components, ($\varepsilon_{\parallel}$) and ($\varepsilon_{\perp}$), but the dielectric tensor is rotated across the wall. Consequently, the local tip-induced optical field couples to different tensor projections on the two sides of the wall. This modifies the local tip–sample polarizability and leads to either enhanced or suppressed near-field response at the twin wall relative to the surrounding bulk domains. In this sense, any dynamic polarization charge appearing in the optical response should be understood as the optically induced polarization associated with the phonon-polaritonic excitation. It is already included in the frequency-dependent anisotropic permittivity tensor and should not be interpreted as a separate sheet of charges localized at the wall. The wall contrast is therefore a consequence of the rotated anisotropic dielectric response and the resulting boundary conditions, not of a pre-existing charge discontinuity between adjacent domains.

Action 3.2: We have changed all the references of canalisation of polaritons at TW in both main text and supplementary information to confinement of THz-fields and added a comment clarifying that TWs are uncharged interfaces (page 2, lines 9-12), and reproduced here for the sake of clarity:

"These regions are delineated by TWs, which are mirror planes, that form a well-defined pattern through the material, constituting uncharged, non-polar interfaces aligned along specific crystallographic angles, separating two domains of the same material with distinct orientations of their anisotropy axis."

Question 3.3:

In this work, the crystallographic directions are predefined and lack tunability. What is the novelty or practical applications of the proposed architecture? How does the proposed LaAlO3 platform integrate with other on-chip optical components?

Answer 3.3: We thank the reviewer for raising this important point. This question is closely related to Question 3.1, where we discussed the advantages of the LAO twin-wall platform compared with other canalized or highly directional polaritonic systems.
We agree that the crystallographic directions of the twin walls are not freely reconfigurable in the same way as the twist angle in van der Waals heterostructures or the geometry of patterned metasurfaces. However, this apparent limitation also constitutes one of the main advantages of the platform: the twin walls are naturally occurring, atomically sharp, crystallographically defined, and extend through the bulk crystal without the need for nanofabrication, lithographic patterning, or stacking of ultrathin flakes. As a result, LAO provides a robust, pattern-free platform for deeply subwavelength near-field confinement in a bulk material. Regarding tunability, we stress that LAO twin walls are crystallographically pinned in an unstrained crystal, but they are not necessarily immutable. As discussed in our response to Question 3.1, recent work shows that ferroelastic twin-wall patterns in LAO can be modified by external strain, including rotation of zig-zag walls and removal of selected vertical walls
The novelty of the proposed architecture is therefore not based on arbitrary post-fabrication reconfigurability, but on the use of intrinsic ferroelastic domain walls as functional polaritonic elements. In contrast to conventional approaches based on patterned resonators, metasurfaces, or twisted van der Waals crystals, the optical confinement in LAO is bound to structural mirror planes that are stabilized by the ferroelastic domain pattern itself. This enables spatially persistent, twin-wall-bound near-field localization along predefined crystallographic paths. Such deterministic and naturally protected channels could be useful for THz and mid-infrared nanophotonic routing, frequency-selective near-field confinement, polaritonic interconnects, and nanoscale sensing.
Regarding integration with on-chip optical components, LAO is particularly attractive because it is compatible with established oxide thin-film growth and heteroepitaxial integration routes. High-quality LAO can be grown on $SrTiO_3$ (STO), STO can be integrated on silicon (see [10.1021/acs.cgd.5c00103]), and a wide range of functional oxides and other materials can be subsequently grown on LAO ($PbTiO_3$, $BaTiO_3$, $LaNiO_3$, TiO_2, etc). This makes the LAO twin-wall platform compatible with multilayer oxide heterostructures and potentially with silicon-based photonic architectures. In this context, the twin walls would act as intrinsic, crystallographically defined near-field channels embedded within an epitaxial oxide platform, while additional on-chip elements, such as metallic antennas or functional overlayers, could be fabricated or grown on top to launch, detect, or modulate the confined polaritonic fields. Thus, although the twin-wall directions are crystallographically defined, LAO offers a realistic route toward integration with oxide electronics, hybrid photonics, and silicon-compatible nanophotonic devices.

Action 3.3: Please see action 3.1

Question 3.4:

The near-field optical scans are measured within the Reststrahlen bands, but no obvious polariton fringes are detected. What is the reason for this? Also, will the topology (either elliptical or hyperbolic) of the in-plane isofrequency contour affect the contrast inversion?

Answer 3.4: We thank the reviewer for this insightful question. Regarding the first part of the reviewer's question, about the observation of polariton fringes, we stress that Figure S9 demonstrates the excitation of surface phonon polaritons at the (001) interface of LAO launched by an Au bar through SINS spectroscopy, where the polaritonic fringes are found and fitted for the extraction of the polaritonic dispersion. Notice that, Figure S9a depicts through a yellow dashed line the real extension of the SINS spectrum shown in Figure S9c, where it can be appreciated that the polaritonic fringes cross the different LAO domains with very little effect in its propagation. However, we would like to stress that the presence of the TWs has still an appreciable effect on the PhP propagation, as it precluded us to extract a consistent propagation length of the surface phonon polaritons as a function of the frequency. As a consequence, we conclude that the lack of polaritonic fringes in the SNOM images taken next to the TWJs, such as the ones shown in Figure 3 of the main text, is due to a very inefficient backreflection of the phonon polaritons at the TWs. Note that, TWs do not correspond to an interface between two different materials. Rather, they separate regions of the same material, with the main difference being the orientation of the optical axes. Since LAO is a pseudocubic material, the dielectric contrast associated with this optical-axis rotation is relatively small. This situation is very different from the commonly studied case of an α-MoO_3 flake on SiO_2. In that case, polaritons propagating in the α-MoO_3 layer and reaching the physical edge of the flake encounter a strong dielectric discontinuity, namely the α-MoO_3/air edge. This produces an efficient polariton back-reflection and consequently, clear interference fringes can be observed near the flake edge.

Regarding the second part of the reviewer's question, the topology of the in-plane isofrequency contour can influence the propagation direction, wavelength, and damping of the polariton modes. Therefore, an elliptical or hyperbolic contour may affect where polariton interference fringes would appear and how strong or directional they would be, provided that a sufficiently strong reflector is present. However, the contrast inversion observed in our measurements occurs between 266 and 269 cm^{-1} (8.06 and 7.96 *THz*), i.e., within the frequency range where the in-plane isofrequency contour remains elliptical (see Figure Response 3). The hyperbolic regime only occurs at higher frequencies, between approximately 275 and 278.5 cm^{-1}. This indicates that the observed contrast inversion is not associated with an elliptical-to-hyperbolic topological transition of the polariton dispersion. Instead, the contrast inversion arises from the frequency-dependent local near-field response of differently oriented ferroelastic domains within the Reststrahlen band. The topology of the in-plane isofrequency contour may modify the propagation properties of polaritons, and thus could influence the appearance of interference fringes. In our case, however, the weak polariton back-reflection at the LAO twin walls prevents the formation of visible standing-wave fringes.

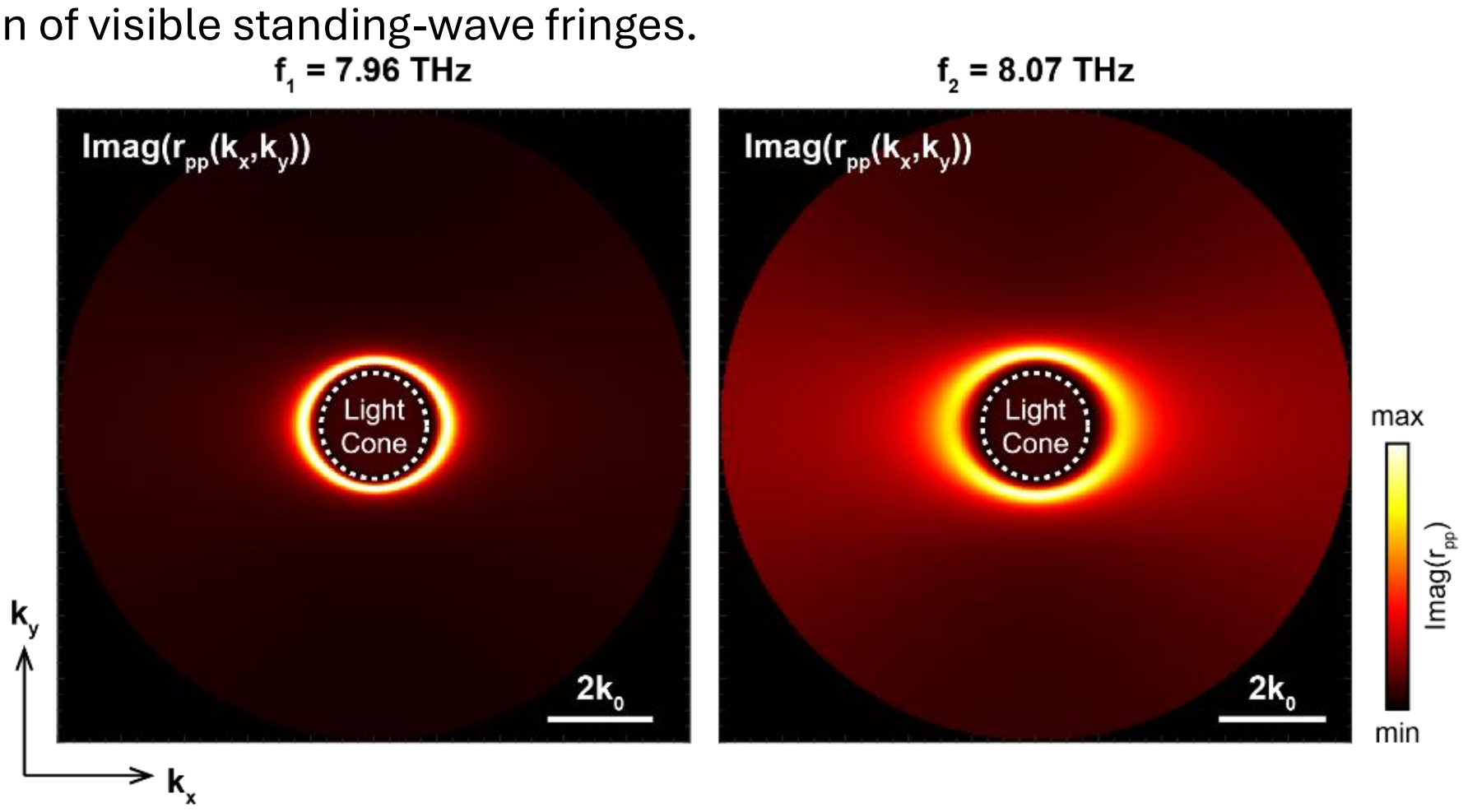

Figure Response 3. Calculated in-plane isofrequency contours of LAO at f_1=7.96 THz (left panel) and f_2=8.07 THz (right panel). Both contours are closed and elliptical, demonstrating that the experimentally observed bright/dark contrast inversion at the twin walls occurs within the elliptical polaritonic regime and is therefore not caused by an elliptical-to-hyperbolic topological transition of the polariton dispersion.

Action 3.4: We highlighted the observed fringe in SNOM and SINS measurements and the missing reproduction in FEM-simulations in the manuscript and we have added new sections of the Supplementary Information, for a better clarification of these points to the readers and reproduced below.

In the main text section "FEM simulations and origin of the contrast" we have added (page 6, lines 8 to 10):
"Merely the weak experimental intensity gradients next to some walls are not reproduced by the simulations. These gradients are attributed to the convolution of 2 effects: surface-PhPs launched by the tip and weakly reflected by the TW, and illumination-related effects. In fact, in complementary synchrotron infrared nano-spectroscopy (SINS) measurements, the excitation of non-strongly confined SPhPs at the surface of bulk LAO(001) through an Au structure has been confirmed in this spectral regime (see Methods and Supplementary Information S2), demonstrating that propagating SPhPs travel through the LAO(001) surface crossing the ferroelastic TWs with very little impact on their propagation."

And in Supplementary Information Section S2:

"... However, it clearly affects the damping term (the propagation length L_p), extremely sensitive to inhomogeneities at the surface of the polaritonic material. This very weak back-reflection also explains why only a weak polaritonic interference fringes are observed near the TWs in the s-SNOM scans shown in Fig. 2 of the main text: the SPhPs are predominantly transmitted across the ferroelastic domain boundaries, with only a small reflected component insufficient to generate a detectable standing-wave pattern. Therefore, ..."

Question 3.5:

In this work, the crystallographic domains are represented as long vertical (010) TWs intersected by the zig-zag TW set. It is also noted that the width along the (100) direction of domains I and II varies. Are these patterns formed naturally and fixed for a specific device? Does the pattern vary from sample to sample? And what are the physical factors that affect the domain distribution?

Answer 3.5: The observed patterns in the investigated sample formed naturally during the growing process and are fixed as long as strain or heat are not applied. As LAO is a ferroelastic material, it is susceptible to strain, and the TWs either move or even disappear under strain, as shown in a recent paper from our group [https://arxiv.org/abs/2604.28183]. The pattern can naturally be fully erased when heating the crystal above its critical temperature and the exact pattern, especially the size of the domains which form when the crystal is cooled down depends on the speed of the cooling process and dimension/aspect ratio of the crystal itself.

Action 3.5: We added a comment addressing the tunability of domain walls with a reference to the recent paper of our group [https://arxiv.org/abs/2604.28183] (page 2, lines 24-26), and reproduced here for the sake of clarity:

"Furthermore, as part of the ferroelastic material class, it has been recently shown, that its crystal structure and domain arrangement can be influenced via applied strain [33]"

Question 3.6:

In Fig.1 and 2, the authors present RBs and permittivity along and orthogonal to the optical axis. However, the PhP modes are investigated within the (001) surface. The effective permittivity along (100) and (010) should be considered rather than simply using the optical constants along and orthogonal to the optical axis.

Answer 3.6: We thank the reviewer for this comment. Indeed, this was something we considered ahead of the publication as it can often feel more intuitive to describe the dielectric permittivity components along the pseudo-cubic crystallographic directions. To achieve this, the tensor, defined with respect to the optical axis along [111], needs to be rotated into an orthonormal laboratory system. In the following, the tensors for the pseudo-cubic unit cell system and the laboratory system are given

$$\varepsilon_{[111]}=\begin{pmatrix}\varepsilon_{\perp} & 0 & 0\\ 0 & \varepsilon_{\perp} & 0\\ 0 & 0 & \varepsilon_{\parallel}\end{pmatrix},\ \varepsilon_{lab}=\begin{pmatrix}\frac{2\varepsilon_{\perp}+\varepsilon_{\parallel}}{3} & \frac{\varepsilon_{\parallel}-\varepsilon_{\perp}}{3} & \frac{\varepsilon_{\parallel}-\varepsilon_{\perp}}{3}\\ \frac{\varepsilon_{\parallel}-\varepsilon_{\perp}}{3} & \frac{2\varepsilon_{\perp}+\varepsilon_{\parallel}}{3} & \frac{\varepsilon_{\parallel}-\varepsilon_{\perp}}{3}\\ \frac{\varepsilon_{\parallel}-\varepsilon_{\perp}}{3} & \frac{\varepsilon_{\parallel}-\varepsilon_{\perp}}{3} & \frac{2\varepsilon_{\perp}+\varepsilon_{\parallel}}{3}\end{pmatrix}$$

Whereas the effective permittivity tensor ε_{lab} is more directly linked to the sample surface and, hence, represents e.g. the elliptic shape of the propagating polariton on the surface (see marked components), the tensor structure is clearly more complex as its representation aligned with the optical axis $\varepsilon_{[111]}$. Moreover, considering the four different domains in LAO, the effective permittivity tensor for each individual domain would need to be specified (given by a 90° rotation of the initial tensor). Finally, for the near-field signal it is not enough to only take the in-plane components of the permittivity into account as the out-of-plane component is essential to describe the near-field coupling and contrast behaviour.

Hence, we decided that it is clearer to the readers to introduce the permittivity tensor aligned with the optical axis in our manuscript. However, we agree with the reviewer that the effective permittivity tensor is of interest for the surface description and hence added its representation to the supplementary information.

Action 3.6: Added to the supplementary information is the decomposition of the optical tensor along each of the crystallographic axes (see new Supplementary Section S8, and reproduced below for the sake of clarity), and an appropriate sentence was added to the main text pointing towards this in the supplementary information for the interested reader (page 2, lines 44, 45).

Changes introduced in page 2 of the main text: "The full tensor and its decomposition along the crystallographic axes can be found in the SI, section S8"

Supplementary Section S8: On the effective permittivity for the (100) and (001) planes

As within our work the near-field signal on a LAO(001) surface is investigated, but the permittivity tensor is aligned with the optical axes along the crystallographic [111] direction and its equivalent rotations, here the effective permittivity on the LAO(001) surface for two exemplary domains is calculated. For a uniaxial crystal whose optical axis lies along the direction $\vec{n}$, the permittivity is given by the following relationship:

$$\hat{\varepsilon}=\varepsilon_{\perp}\hat{I}+(\varepsilon_{\parallel}-\varepsilon_{\perp})\vec{n}\otimes\vec{n}^{T}$$

With $\otimes$ the dyadic product.

In the case of domain I, $\vec{n}$ is given by:

$$\vec{n}_{\mathrm{I}}=\frac{1}{\sqrt{3}}\begin{pmatrix}1\\1\\1\end{pmatrix}$$

And, as a consequence,

$$\vec{n_{\mathrm{I}}}\otimes\vec{n_{\mathrm{I}}}^{T}=\frac{1}{3}\begin{pmatrix}1&1&1\\1&1&1\\1&1&1\end{pmatrix}$$

So:

$$\hat{\varepsilon}_{\mathrm{I}}=\varepsilon_{\perp}\begin{pmatrix}1&0&0\\0&1&0\\0&0&1\end{pmatrix}+\frac{(\varepsilon_{\parallel}\text{-}\varepsilon_{\perp})}{3}\begin{pmatrix}1&1&1\\1&1&1\\1&1&1\end{pmatrix}=\frac{1}{3}\begin{pmatrix}\varepsilon_{\parallel}+2\varepsilon_{\perp}&\varepsilon_{\parallel}\text{-}\varepsilon_{\perp}&\varepsilon_{\parallel}\text{-}\varepsilon_{\perp}\\\varepsilon_{\parallel}\text{-}\varepsilon_{\perp}&\varepsilon_{\parallel}+2\varepsilon_{\perp}&\varepsilon_{\parallel}\text{-}\varepsilon_{\perp}\\\varepsilon_{\parallel}\text{-}\varepsilon_{\perp}&\varepsilon_{\parallel}\text{-}\varepsilon_{\perp}&\varepsilon_{\parallel}+2\varepsilon_{\perp}\end{pmatrix}$$

Which is the shape the dielectric tensor takes when written in the laboratory frame given by the orthonormal vectors [100], [010], and [001]. Therefore, the (001) plane projection will be given by:

$$\hat{\varepsilon}_{\mathrm{I}(001)}=\begin{pmatrix}\varepsilon_{xx}&\varepsilon_{xy}\\\varepsilon_{yx}&\varepsilon_{yy}\end{pmatrix}=\frac{1}{3}\begin{pmatrix}\varepsilon_{\parallel}+2\varepsilon_{\perp}&\varepsilon_{\parallel}\text{-}\varepsilon_{\perp}\\\varepsilon_{\parallel}\text{-}\varepsilon_{\perp}&\varepsilon_{\parallel}+2\varepsilon_{\perp}\end{pmatrix}.$$

Note that, for domain III we find the exact same result, but with the tensor elements ε_{xz}, $\varepsilon_{yz,}$ ε_{zx}, and ε_{zy} being multiplied by -1.

In the case of domain II, $\vec{n}$ is given by:

$$\vec{n}_{\mathrm{II}}=\frac{1}{\sqrt{3}}\begin{pmatrix}-1\\1\\1\end{pmatrix}$$

And, as a consequence,

$$\vec{n_{\mathrm{II}}}\otimes\vec{n_{\mathrm{II}}}^{T}=\frac{1}{3}\begin{pmatrix}1&-1&-1\\-1&1&1\\-1&1&1\end{pmatrix}$$

So:

$$\hat{\varepsilon}_{\mathrm{II}}=\varepsilon_{\perp}\begin{pmatrix}1&0&0\\0&1&0\\0&0&1\end{pmatrix}+\frac{(\varepsilon_{\parallel}\text{-}\varepsilon_{\perp})}{3}\begin{pmatrix}1&-1&-1\\-1&1&1\\-1&1&1\end{pmatrix}=\frac{1}{3}\begin{pmatrix}\varepsilon_{\parallel}+2\varepsilon_{\perp}&-(\varepsilon_{\parallel}\text{-}\varepsilon_{\perp})&-(\varepsilon_{\parallel}\text{-}\varepsilon_{\perp})\\-(\varepsilon_{\parallel}\text{-}\varepsilon_{\perp})&\varepsilon_{\parallel}+2\varepsilon_{\perp}&\varepsilon_{\parallel}\text{-}\varepsilon_{\perp}\\-(\varepsilon_{\parallel}\text{-}\varepsilon_{\perp})&\varepsilon_{\parallel}\text{-}\varepsilon_{\perp}&\varepsilon_{\parallel}+2\varepsilon_{\perp}\end{pmatrix}$$

Here, the (001) plane projection is given by:

$$\hat{\varepsilon}_{\mathrm{II}(001)}=\begin{pmatrix}\varepsilon_{xx}&\varepsilon_{xy}\\\varepsilon_{yx}&\varepsilon_{yy}\end{pmatrix}=\frac{1}{3}\begin{pmatrix}\varepsilon_{\parallel}+2\varepsilon_{\perp}&-(\varepsilon_{\parallel}\text{-}\varepsilon_{\perp})\\-(\varepsilon_{\parallel}\text{-}\varepsilon_{\perp})&\varepsilon_{\parallel}+2\varepsilon_{\perp}\end{pmatrix}.$$

Similar to domains I and III, again domain IV shows the same result as domain II, just with the tensor elements ε_{xz}, $\varepsilon_{yz,}$ ε_{zx}, and ε_{zy} multiplied by -1.

The (001)-plane projection $\hat{\varepsilon}_{(001)}$ in both cases describes an ellipse (see SI S5 Fig. S15) with its principal axes aligned along 45° in the surface grid, and its orientation in the case of domains I and III rotated by 90° compared to the ellipse of domain II and IV, shown by the flipped sign of the off-diagonal elements of $\hat{\varepsilon}_{(001)}$. However, this in-plane projection ignores all elements with a z-component, which are necessary to fully explain the observed contrast behaviour at the TWs.

Question 3.7:

The authors report on frequency-dependent contrast inversion at TWJs and claim it is related to the Mie resonance condition. How are the PhPs at TWs coupled with the Mie resonance? A more intuitive figure and elucidation should be added to clarify the theoretical framework.

Answer 3.7: We thank the reviewer for pointing out the lack of clarity. In order to qualitatively discuss the contrast inversion, the standard quasi-electrostatic dipole model by Knoll et al. [10.1016/S0030-4018(00)00826-9] was adapted in order to include two different sample regions with rotated permittivities. In this model the tip is approximated as a point dipole induced in a small isotropic sphere. Moreover, the interaction of this tip with small local sample volumes of the two adjacent domains, equally represented by small spheres, is calculated. Note: The dipole model in its quasi-electrostatic character does not predict propagating phonon polaritons, only the scattering cross section (equivalent to the near-field response) at a localised point (0d). However, due to the near-field interaction between the tip and sample, resonance of the near-field coupled systems are found for Re(ε_{sample}) = -3...-1 (depending on tip-sample distance) reflecting an polariton-based enhancement even in this electrostatic model. Mie-scattering in the electrostatic limit can be used to calculate the scattering of light on small spherical particles and was therefore applied here. Whereas this is a common approach for the tip-scattering, the representation of relevant sample areas by additional spheres is new in this adapted model. This approach has been chosen in order to enable arbitrary orientations of the anisotropic sample permittivity tensor in each domain due to the rotated optical axis. However, this analytic model is strongly limited by these approximations and, hence, can only be applied to qualitatively discuss the contrast inversion and its general dependency on key parameters. For further details of the adapted dipole model and its limitations please see SI S4. There, the full theoretical derivation and more descriptive figures can be found. Note that the FEM calculations, showing a good agreement with experimental results, are free from any assumption of the presence of Mie-resonances. In conclusion, the Mie-like character of the resonance is rather a starting point to describe the localised fields and the near-field coupling in SNOM. In fact, the exact spectral position of the near-field resonance is depending on additional parameters such as the tip-sample distance. In our initial manuscript, it seems that the marking of the Mie-resonance condition in Fig. 2C has been unintentionally emphasised this type of resonance character (as also pointed out by other reviewers). We therefore adapted this figure in the revised manuscript to now highlight the resonance regime typically observed in SNOM for Re(ε)=-3...-1 as reported in literature [10.1103/PhysRevB.100.035444, 10.1021/acs.nanolett.3c03369, 10.1103/PhysRevLett.100.256403].

Action 3.7: We clarified that the adapted dipole model is only used to qualitatively discuss the contrast inversion (page 6, lines 12-15), adapted Fig 2c and point to the SI for more details about the dipole-model and its limitations (page 7, lines 3-5). All these changes are reproduced in the following for the sake of clarity:

Changes introduced in page 6:
"In order to qualitatively discuss why different walls brighten or darken at different frequencies, the analytical point-dipole model commonly used to describe s-SNOM contrast was adapted [42-44, 50]."

Changes introduced in page 7:
"... simulations are required, such as the FEM-simulation carried out within this work. For more details on the adapted dipole model and its limitations, please see SI S4."

Question 3.8:

In the practical near-field scanning microscopy, the AFM tip for near-field information collection oscillates vertically. What is the reason that the near-field probe is presented by a small metallic sphere with isotropic polarizability in the analytical model? Please explain.

Answer 3.8: It is correct and in fact crucial, that in a SNOM-experiment the tip oscillates vertically, as this is what allows for the separation of undesired far-field and near-field contributions to the measured signal. In order to include this oscillation in the theoretical model, the tip is represented by a dipole, with its height above the sample surface being modulated in time. As the near-field coupling between tip and sample depends strongly non-linearly on the distance, the probed near-field signal is modulated at higher harmonics of the tip oscillation frequency [10.1016/S0030-4018(00)00826-9]. When the measured signal (modulated by the tip frequency) is Fourier transformed, the higher harmonics yield the desired background-free near-field signal, suppressing the non-desired far-field contribution. In the analytical model as applied here, the scattering cross section is calculated, which is equivalent to the near-field signal.
Still, (even for the modulated version of the analytical model) the near-field probe may be represented by a small metallic sphere with isotropic polarisability, as long as the distance dependent coupling is included, as it is typically done in the standard dipole model [10.1016/S0030-4018(00)00826-9, 10.1002/9783527618156]. More complex models, such as the finite dipole model, do more accurately describe the elongated tip shape in z-direction, however, equally introduce a height modulation to describe the vertical AFM tip oscillation. This more accurate model, unfortunately is limited to purely out-of-plane dipoles, which reduces possible orientations of the tip polarisability [10.1002/9783527618156] and, moreover, implementation of anisotropic samples is more challenging. Given that in our case the rotation of the sample dipoles in three spacial dimensions was essential, such limitations out-weighted the benefits of more complex models. Hence, we represent our system with the simplest approximation of a spherical tip shape which is applied here solely to qualitatively describe the coupling mechanism. For more accurate theoretical descriptions, we apply FEM as shown in the manuscript.

Action 3.8: Added a reference to the SI with a detailed explanation of the adapted dipole model and its limitations (page 7, lines 3-5), and reproduced here for the sake of clarity:

Changes introduced into page 7:

"… simulations are required, such as the FEM-simulation carried out within this work. For more details on the adapted dipole model and its limitations, please see SI S4."

Changes introduced into Supplementary Information Section S4:

"**Supplementary Section S4:** Analytical Point-Dipole Model for Twin-Domain Walls

In order to analytically describe the fundamental interaction of a s-SNOM probe with the LAO twin-domain walls (TW), we modify here the analytical point-dipole model as originally introduced by Knoll and Keilmann [8] and thereby extend it for interaction with an anisotropic sample that includes a domain wall, i.e. a boundary between sample areas with different permittivity tensors. Our model assumes an infinitely extended sample surface located at $z = 0$, with the probe in air above ($z > 0$), and the sample below ($z = 0$). The TW, located at $x = 0$ separates the sample half-space into two quarter spaces (see Fig. S12A). The two adjacent sample domains are then represented by two laterally separated polarizable spheres, here placed at $z = -h$ and $x = \pm h$, with in general arbitrarily oriented anisotropic permittivity tensors. Similar to the conventional dipole-model, the tip is assumed to be represented by an isotropic sphere and is placed at a height $z = h$, directly above the TW-surface junction ($x = 0$), i.e. symmetrically between the two sample spheres (see Fig. S12A)."

Question 3.9:

The authors present simulated results of frequency-dependent contrast inversion at TWJs both in Fig. 2 and Fig. 4 at f3 = 8.06 THz and f4 = 8.24 THz, while the near-field experimental validation is performed at f1 = 7.96 THz and f2 = 9.07 THz. Please explain this mismatch, and also theoretically elaborate on the physics of the contrast at f3 = 8.06 THz and f4 = 8.24 THz. The simulation results at f1 = 7.96 THz and f2 = 9.07 THz should be added and discussed.

Answer 3.9: We thank the reviewer for raising this question. Regarding the first part of the reviewer's question, we note that in the simulation we have employed the point-dipole model approximation [10.1016/S0030-4018(00)00826-9] as it is widely known as a simple qualitative and computationally efficient description of the s-SNOM tip–sample interaction that can predict the existence of near-field resonances reasonably well [10.1002/adma.201908176; 10.1038/nphoton.2016.44], and in particular, the phonon polariton resonant near-field interaction [10.1038/nature00899]. However, it is known to have quantitative limitations due to the oversimplification of the tip-sample interactions, as it does not take into consideration the antenna effect of the tip, the distribution of the charges next to the very tip apex and retardation effects, for example [10.1364/OE.15.008550]. In other words, the point dipole model demonstrates that the experimentally measured resonance is not only the bare material resonance, but a hybrid tip-sample resonance. As a consequence, we used the point-dipole approximation to model the s-SNOM contrast because it provides a computationally efficient description of the tip–sample interaction while still capturing the qualitative near-field contrast mechanism at the LAO twin walls

Regarding the second part of the reviewer's question, about the physical origin of the contrast at f_3 and f_4, we have prepared Figure Response 4 to delve into the physical origin of the twin-wall contrast. Figure Response 4a shows the left-hand side of the simulations presented in Fig. 2f,g of the main text, which reproduce the s-SNOM contrast inversion at f_3 = 8.06 THz (left panel) and f_4 = 8.24 THz (right panel), scanning laterally the dipole along the LAO surface and the TW regions. In order to understand the effect of the TW on the electric field distribution, we have calculated the electric-field, ($Re(E_z)$), for two positions of the dipole (see Figure Response 4b): in the middle

of a ferroelastic LAO domain (upper-half of the panels) and at the TW position (lower-half of the panels). At both frequencies, the presence of the twin wall strongly modifies the polaritonic near field generated by the dipole. At f_3, the twin wall mainly redistributes the phase and amplitude of the polaritonic field. At f_4, by contrast, the field becomes strongly enhanced and spatially concentrated at the twin wall. This effect is further demonstrated by having a look at the distribution of the in-plane time-averaged Poynting vector ($\langle S \rangle$, see Figure Response 4c) at both frequencies. The black solid contour lines indicate a fixed value of the Poynting-vector magnitude and are included to highlight the spatial redistribution of the in-plane energy flow for each frequency and dipole position. These calculations show that the twin wall does not behave as a simple geometrical ridge or as an interface with a large positive–negative permittivity contrast. Instead, it modifies the local polaritonic field through the rotated anisotropic dielectric response of the adjacent domains. At f_3, the in-plane energy flow is reduced along the twin wall, consistent with the dark contrast observed in the simulated s-SNOM image. At f_4, the energy-flow distribution becomes concentrated along the twin wall, consistent with the bright near-field contrast.

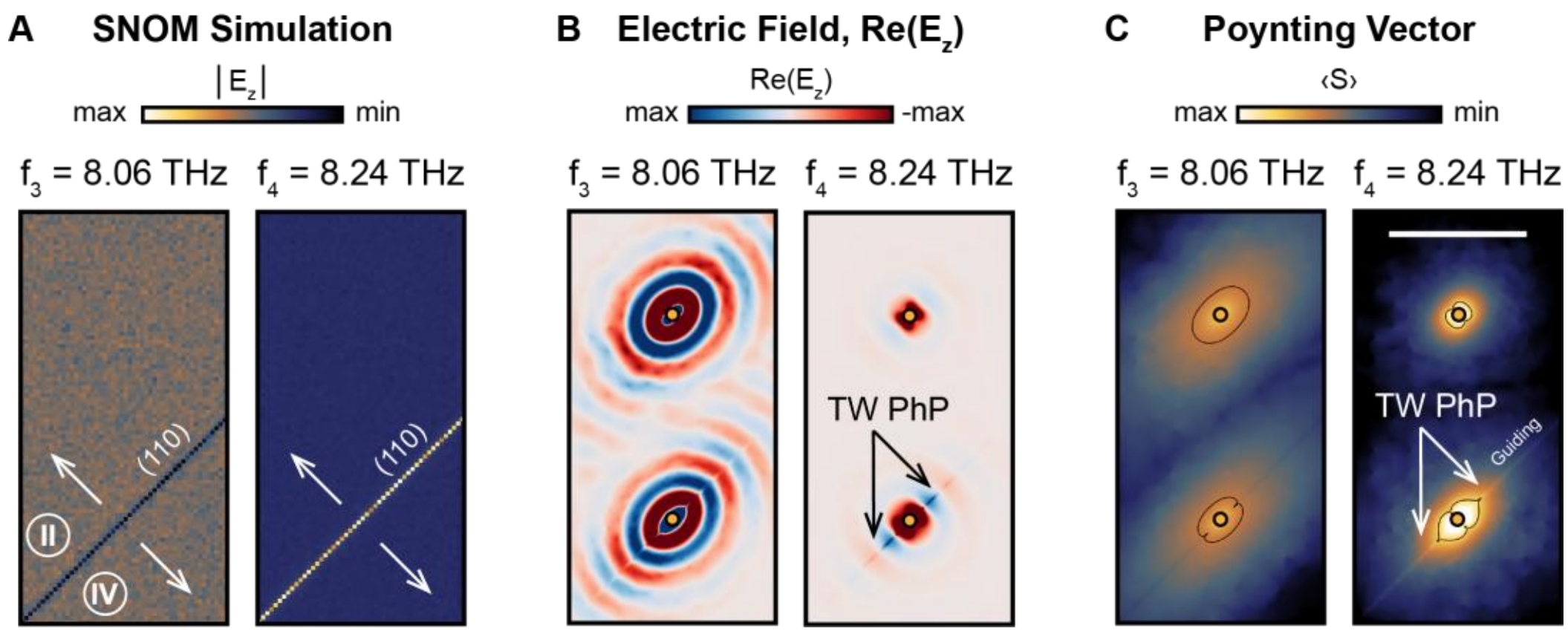

Figure Response 4. On the contrast of the TWs between f3 and f4. **A** FEM simulation mimicking the s-SNOM response by scanning laterally the dipole along the LAO surface and TWs. The images correspond to the left-hand side of the simulations presented in Fig. 2f,g of the main text. **B** Real part of the electric field distribution, ($Re(E_z)$), at both frequencies for two positions of the dipole: in the middle of a LAO domain (upper part of the panels) and on top of a TW (lower part of the panels). The effect of the TW on the electric field distribution is clearly visible. **C** In-plane time-averaged Poynting vector, $\langle S \rangle$, at both frequencies for the same two positions of the tip. The figures also show a contour of the distribution, demonstrating that the TW acts as a repelling channel for the lowest frequency and it guides the energy along it for the highest frequency.

We note that a complete modal description of phonon-polariton propagation bound to LAO twin walls, including dispersion relations, energy-flow profiles, and propagation lengths, requires a dedicated analysis beyond the scope of the present manuscript and that is being performed for a future and dedicated publication. The purpose of the additional simulations presented here is therefore not to claim a full modal propagation analysis, but to clarify the physical origin of the bright/dark contrast at $f_3$and f_4. These simulations show that the contrast inversion is caused by a frequency-dependent redistribution of the local near-field amplitude between the twin wall and the surrounding bulk domains.

On the other hand, following the last suggestion of the reviewer, about adding and discussing the simulation results at f_1=7.96 THz and f_2=8.07 THz, we would like to stress that we have selected f_3=8.06 THz and f_4=8.24 THz as representative cases of the bright / dark contrast inversion of

the walls and that they validate the qualitative TW contrast found in the experiment and in the analytical model. The simulated SNOM images are shown in Figure Response 5 at f_1=7.96 THz (A) and f_2=8.07 THz (B). The contrast of the images is fully-inline with our analytical model prediction, showing the same bright / dark contrast as in our simulation at f_3=8.06 THz, which demonstrates this effect as a consistent response in this frequency window, and not an isolated feature of that particular frequency. The agreement between the simulations at f_1, f_2, and the analytical model supports the interpretation of the near-field signal of the TWs being originated by the frequency-dependent effective polarizability of the wall region.

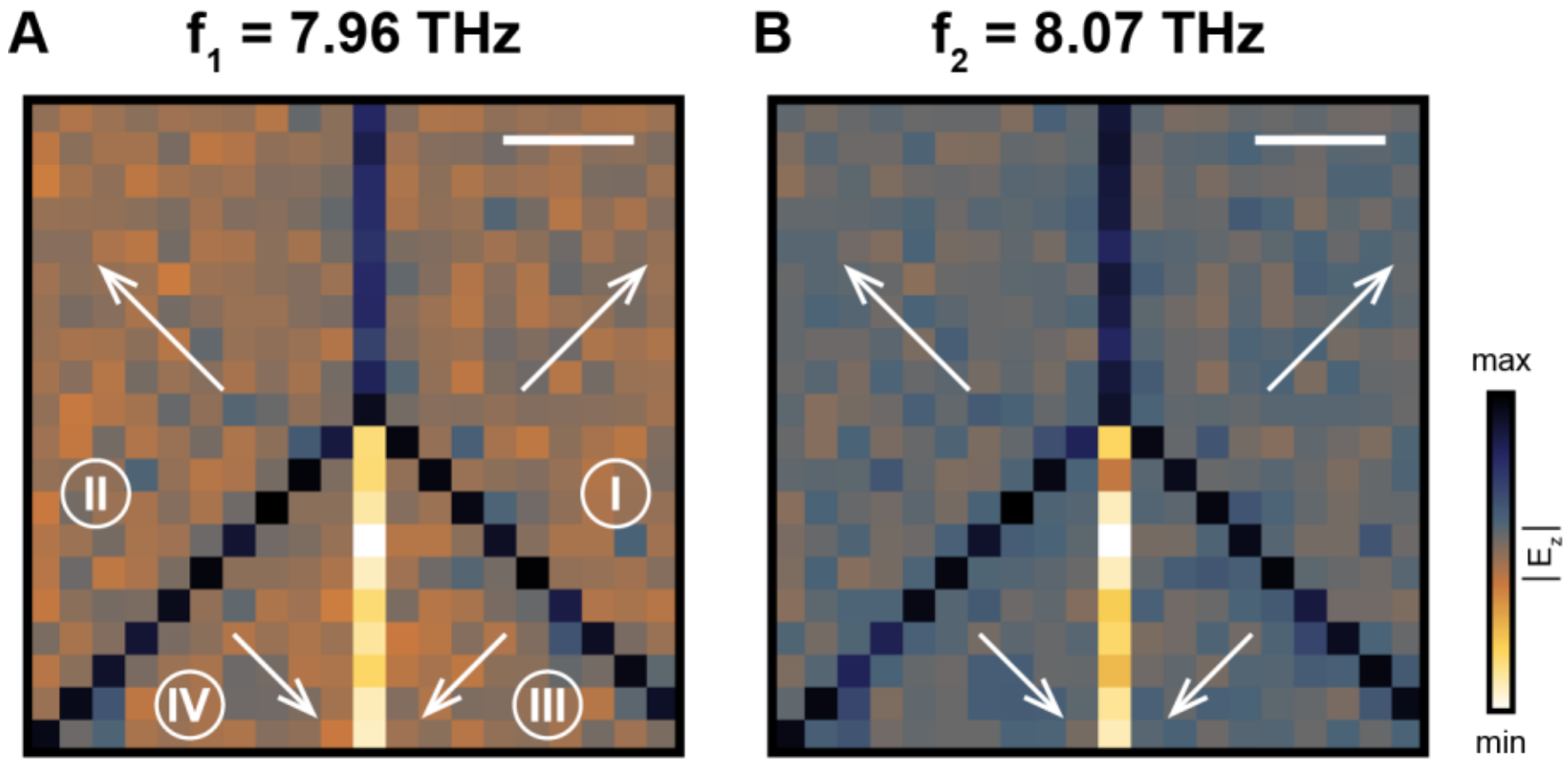

Figure Response 5. FEM simulations of the same TWJ as Figures 2d and 2e at f_1=7.96 THz (A) and f_2=8.07 THz (B), mimicking the SNOM scans. White arrows depict the XY projection of the optical axis for each domain. Each domain is labelled as I, II, III or IV in the same way as the main text. Scale bars: 2 μm.

Action 3.9: We have added a new sections S6 to the Supplementary Information, for a better clarification of these points to the readers and reproduced below:

Supplementary Section S6: Comparison between FEM simulations and experiment at f_1 and f_2

In the main text, the FEM simulations shown in Fig. 2f,g were performed at f_1'=8.06 THz and f_2'=8.24 THz. These frequencies were selected as representative cases because they reproduce the bright/dark twin-wall contrast observed in the experiments and predicted by the analytical model (see SI S4) and illustrate the contrast inversion most clearly within the point-dipole FEM framework. The point-dipole approximation is expected to reproduce the qualitative contrast mechanism, but not necessarily the exact experimental frequency at which the contrast inversion occurs. Therefore, the simulated inversion can appear shifted with respect to the experimentally observed inversion, typically in the order of 2% [12]. To directly address this point, we performed additional FEM simulations at the experimental frequencies f_1=7.96 THz and f_2=8.07 THz, shown in Fig. S17. The simulations at f_1 and f_2 display the same bright/dark twin-wall contrast as the simulation at f_1'=8.06 THz. This confirms that the contrast calculated at f_1' is not an isolated feature of that particular frequency, but rather a robust response within this spectral window. The agreement between the simulations at f_1, f_2, and f_1', together with the analytical model, supports the interpretation that the TW contrast originates from the frequency-dependent effective polarizability of the wall region relative to the surrounding bulk domains. The fact that the experimental contrast inversion occurs between $f_1$and f_2, whereas the point-dipole FEM inversion is shifted toward f_1'–f_2', is consistent with the known limitations of the point-dipole approximation.

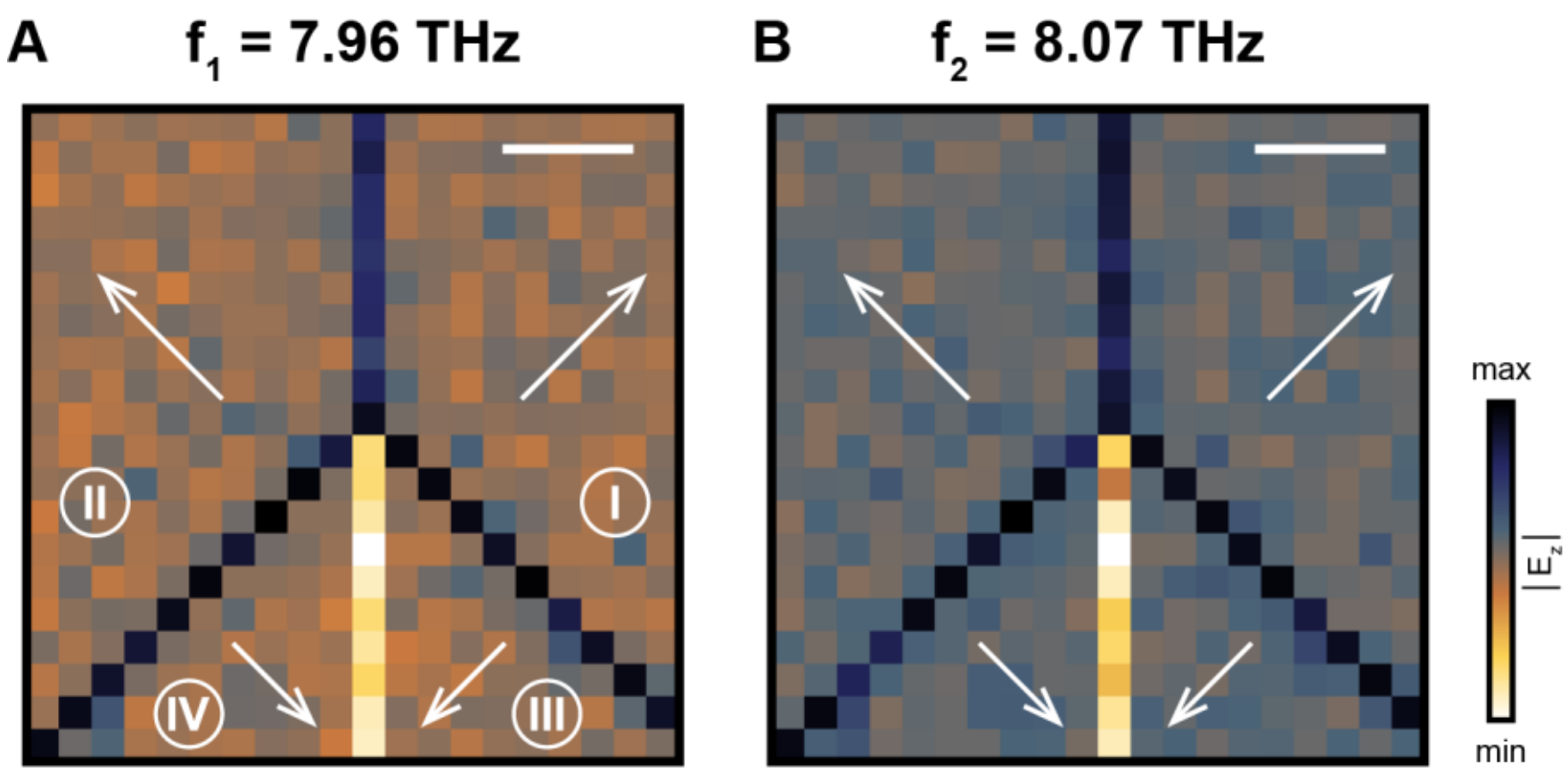

Figure S16. FEM simulations of the same TWJ as shown in Figs. 2d, e of the main text, performed at the experimental frequencies f_1=7.96 THz and f_2=8.07 THz. Panels A and B show the simulated near-field maps at $f_1$and f_2, respectively. White arrows indicate the in-plane projection of the optical axis for each domain. The domains are labelled I–IV following the notation used in the main text. Scale bars: 2 μm.

Question 3.10:

The authors claim "long-range, guided near-field transport". Towards this, an energy profile and electrical field distribution along the propagation direction, and comparison to the propagation length of other materials or physical configurations, should be added.

Answer 3.10: We thank the reviewer for this important comment. We agree that the phrase "long-range, guided near-field transport" could be interpreted as implying a full modal propagation analysis of phonon polaritons along the twin walls, including energy-flow profiles, propagation lengths, and benchmarking against other polaritonic platforms. This was not the intended scope of the original manuscript, which primarily focused on the experimentally observed bright/dark near-field contrast at LAO twin walls and twin-wall junctions, together with the strong lateral confinement of the electromagnetic near field at these walls.
To address the reviewer's request, we have added a quantitative comparison of the spatial decay of the TW-localized near-field response with representative polaritonic configurations based on α-MoO_3. Specifically, we performed additional FEM simulations in COMSOL Multiphysics for three types of systems: (i) a LAO twin-wall junction excited by a vertically oriented dipole, (ii) a single α-MoO_3 flake, and (iii) two rotated α-MoO_3 flakes at the magic angle, where canalised polariton propagation is expected. For the α-MoO_3-based systems, simulations were performed at 27.25 THz (λ_0=11 μm) and 8.67 THz, while the LAO calculation was performed at f_3=8.06 THz, see Figure Response 6
For each case, we extracted near-field line profiles 1 nm above the surface along the TWs (in the LAO case), along the [100] direction (for the single α-MoO_3 flake) and along the canalisation direction (for the two rotated a-MoO3 flakes). The profiles were fitted using a damped oscillatory function:

$$y(x)=y_0+A\cdot\exp\left(-\frac{x}{L_p}\right)\cdot sin\left(\frac{2\pi x}{\lambda_p}+\phi_0\right)\cdot\frac{1}{\sqrt{x}}$$

Where y_0 is an offset, A is the amplitude, L_p is the fitted polaritonic decay length, λ_p is the polaritonic wavelength, ϕ_0 is a phase offset, and the factor $1/\sqrt{x}$ accounts for the geometrical spreading of waves launched by a localized dipolar source. Figure Response 6 shows the calculated $\mathrm{Re}(E_z)$ distributions for all simulated cases. The left panels display the field distributions, while the right panels show the extracted line profiles and corresponding fits. The extracted values of λ_p, L_p and $\beta = \lambda_0/\lambda_p = k_p/k_0$ as well as the quality factor, Q, defined as

$Q=\mathrm{Re}(k_p)/\mathrm{Im}(k_p)=2\pi L_p/\lambda_p$ are summarized in Table Response 2. This comparison shows that the field localised at the LAO twin wall persists over mesoscopic distances (L_p=46.48 μm and 27.49 μm depending on the wall) while remaining laterally confined to the wall region. Importantly, the extracted decay length is one order of magnitude larger than those obtained in established α-MoO_3-based polaritonic systems under similar dipolar excitation conditions. This supports our interpretation that LAO twin walls provide a natural, pattern-free platform for spatially persistent near-field channeling with deep-subwavelength lateral confinement. Furthermore, depending on the wall, the polaritonic quality factor is comparable to those of well-established α-MoO_3-based systems.
However, a full modal analysis of launched twin-wall polaritons, including experimental extraction of propagation lengths of TW polaritons launched from Au structures is the subject of ongoing work and will be presented in a future dedicated publication. As a consequence, we would like to kindly ask the referee to keep the following information confidential and not to be published in the present manuscript.

Theoretical Calculations					
Case	f (THz)	λ_p (μm)	β	L_p (μm)	Q
Single MoO_3	27.25	0.93	11.83	1.56	10.54
Twisted MoO_3	27.25	1.22	9.02	2.19	11.30
Single MoO_3	8.67	3.56	9.71	5.64	9.96
Twisted MoO_3	8.67	4.14	8.35	6.30	9.56
LAO $TW^{\perp}$	8.06	29.50	1.26	46.48	9.90
LAO $TW^{\perp}$	8.06	27.70	1.34	27.49	6.24
Experimental Reports					
Case	f (THz)	λ_p (μm)	β	Lp (μm)	Q
Twisted MoO_3 [10.1038/s41586-020-2359-9]	27.10	-	-	2.77	-
Twisted MoO_3 [10.1021/acsnano.3c06477]	8.67	6.52	5.3	-	-

Table Response 2. Comparison of key polaritonic metrics across different material platforms. For the theoretical calculations, the single- and twisted-MoO_3 flakes simulated at 27.25 THz with a thickness of 200 nm, whereas the single- and twisted-MoO_3 flakes simulated at 8.67 THz with a thickness of 50 nm. The twisted-MoO_3 simulations were performed at the twist angle for which polariton canalization occurs, corresponding to flat bands in momentum space. For the experimental reports, Ref. [10.1038/s41586-020-2359-9] used a heterostructure composed of two MoO_3 flakes, each 117 nm thick, whereas Ref.

[10.1021/acsnano.3c06477] used a twisted bilayer with a 125 nm-thick top flake and a 115 nm-thick bottom flake.

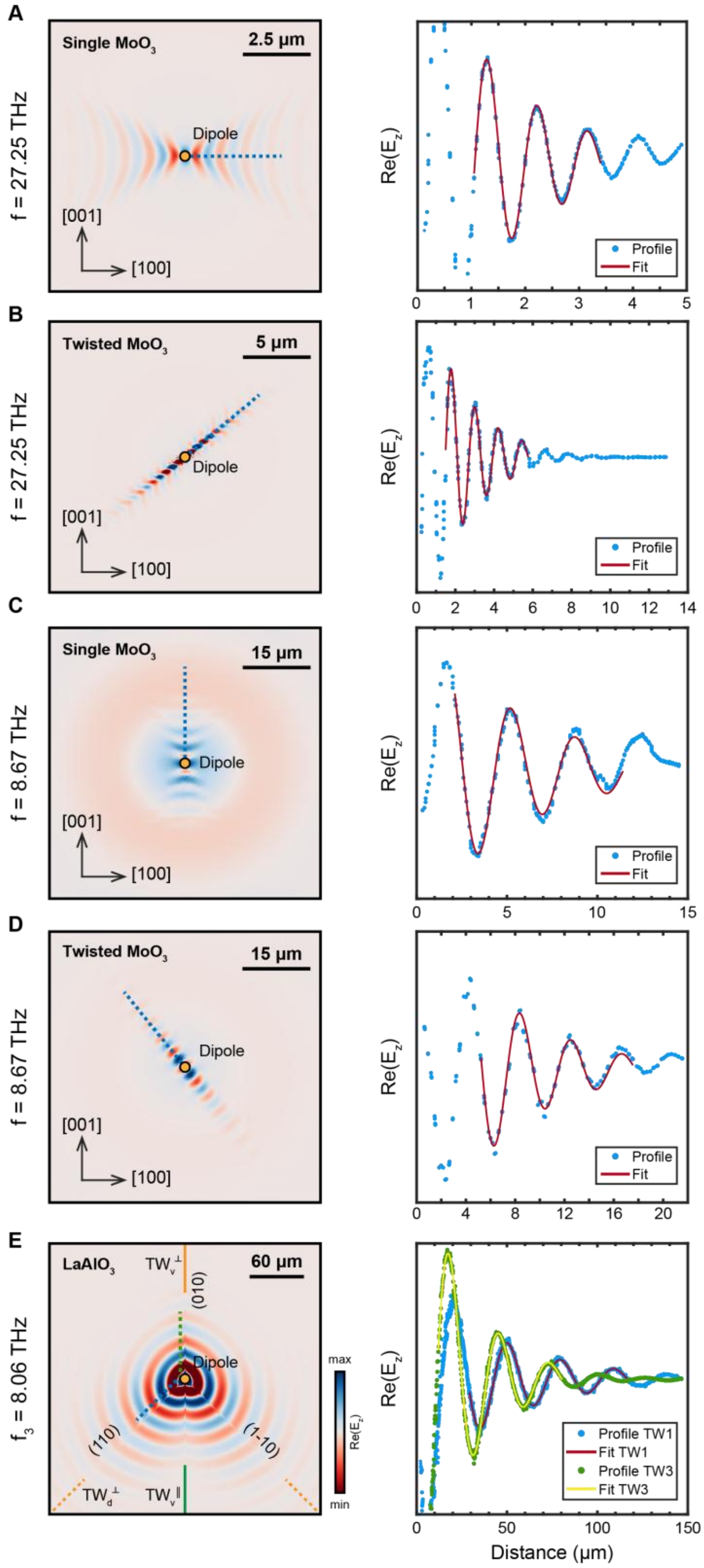

Figure Response 6. FEM simulations of α-MoO3-based systems and LAO TWJ at selected frequencies. A single a-MoO3 flake at f=27.25 THz (λ_0=11μm), B twisted α-MoO_3 flakes at f=27.25 THz, C single MoO_3 flake at f=8.67 THz, D twisted α-MoO_3 flakes at f=8.67 THz and E LAO TWJ at f_3=8.06 THz. Left panels depict the real part of the electric field distribution, $\mathrm{Re}(E_z)$, while right panels show the extracted

profiles along the dashed lines depicted in the left panels alongside its respective fittings to the equation shown above.

Action 3.10: A complete modal analysis of phonon-polariton modes bound to LAO twin walls, including Poynting-vector maps, modal dispersion, and propagation-length extraction from eigenmode calculations, will be the subject of a dedicated future study.

TECHNISCHE UNIVERSITÄT DRESDEN

DRESDEN concept

Bereich Mathematik und Naturwissenschaften Fakultät Physik

Institut für Angewandte Physik – Photophysik – Nanooptik

Technische Universität Dresden, 01062 Dresden

Dr.
Samuel D. Seddon

Telefon: +49-351-463-33740

E-Mail: samuel.seddon@tu-dresden.de

Dresden, 25.08.2026

To the reviewers

We thank you for accepting our manuscript, and for taking the time to help us improve its clarity and quality.

For posterity, the final reviewers comments:

REVIEWERS' COMMENTS

Reviewer #1 (Remarks to the Author):

The authors have done significant revisions according to the reviewers' comments. I now see that the paper has been improved, and thus recommend the publication of this work.

Reviewer #2 (Remarks to the Author):

All my comments have been sufficiently addressed in the revised manuscript. I have no further reservations and support publication.

Reviewer #3 (Remarks to the Author):

I thank the authors for their thorough and well-considered responses to the reviewers' comments. The corresponding revisions have been appropriately incorporated into the manuscript. I therefore recommend the manuscript for publication.

Many thanks again,
Sam Seddon, on behalf of all co-authors
